# Into Deep Water: Optimizing BCL6 Inhibitors by Growing into a Solvated Pocket

**DOI:** 10.1021/acs.jmedchem.1c00946

**Published:** 2021-11-30

**Authors:** Matthew G. Lloyd, Rosemary Huckvale, Kwai-Ming J. Cheung, Matthew J. Rodrigues, Gavin W. Collie, Olivier A. Pierrat, Mahad Gatti Iou, Michael Carter, Owen A. Davis, P. Craig McAndrew, Emma Gunnell, Yann-Vaï Le Bihan, Rachel Talbot, Alan T. Henley, Louise D. Johnson, Angela Hayes, Michael D. Bright, Florence I. Raynaud, Mirco Meniconi, Rosemary Burke, Rob L. M. van Montfort, Olivia W. Rossanese, Benjamin R. Bellenie, Swen Hoelder

**Affiliations:** ^†^Cancer Research UK Cancer Therapeutics Unit and ^‡^Division of Structural Biology, The Institute of Cancer Research, London SM2 5NG, U.K.

## Abstract

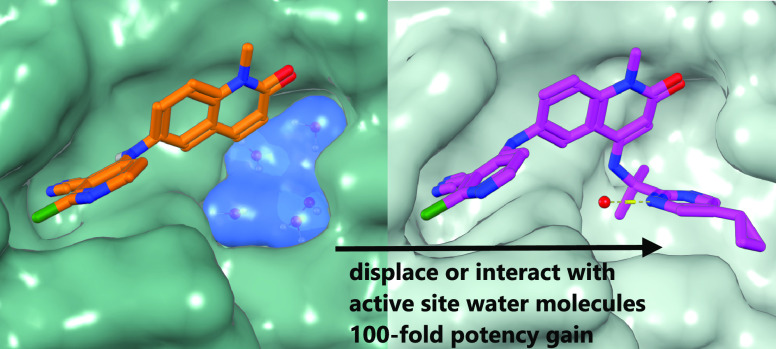

We describe the optimization of modestly active starting points to potent inhibitors of BCL6 by growing into a subpocket, which was occupied by a network of five stably bound water molecules. Identifying potent inhibitors required not only forming new interactions in the subpocket but also perturbing the water network in a productive, potency-increasing fashion while controlling the physicochemical properties. We achieved this goal in a sequential manner by systematically probing the pocket and the water network, ultimately achieving a 100-fold improvement of activity. The most potent compounds displaced three of the five initial water molecules and formed hydrogen bonds with the remaining two. Compound **25** showed a promising profile for a lead compound with submicromolar inhibition of BCL6 in cells and satisfactory pharmacokinetic (PK) properties. Our work highlights the importance of finding productive ways to perturb existing water networks when growing into solvent-filled protein pockets.

## Introduction

Diffuse large B-cell lymphomas (DLBCLs) are the most common subtype of non-Hodgkin lymphoma.^1^ During the immunological process of affinity maturation, B-cells are required to proliferate rapidly, evade growth checkpoint controls, and tolerate ongoing genomic instability. Germinal center B-cells are dependent on the expression of BCL6 (B-cell lymphoma 6 protein), which is a transcriptional factor that binds to corepressor proteins NCOR, SMRT, or BCOR via a peptide-binding surface on its BTB domain dimer.^2,3^ Through this interaction, BCL6 represses genes involved in cell cycle control, cell death, and differentiation.^4^ Most B-cell lymphomas arise from germinal center B-cells.^5−7^ Once oncogenic, the tumor relies on the sustained expression of BCL6.^8^ In the case of DLBCL, blocking the interaction between BCL6 and its corepressors enables the re-expression of repressed genes, which leads to the continuation of B-cell differentiation and cell death.^9^ This highlights potential therapeutic efficacy for inhibitors of this BCL6 protein–protein interaction.

Inhibitors of the BCL6-corepressor protein–protein interaction binding to the BCL6 BTB domain have been described. These include high-affinity peptides,^10^ macrocycles,^11^ as well as small molecule inhibitors.^12−17^ Molecules, which induce degradation of BCL6, have also been reported, including monovalent degraders and a PROTAC.^16−18^

We have previously reported the optimization of hit compound **CCT365386** (**1**) yielding potent BCL6 degraders, including cell-active **CCT369260**.^17^ However, in this benzimidazolone series, we were only able to obtain inhibitors of modest potency. We wished to explore alternative core scaffolds with alternative vectors, which would allow us to improve potency by growing more efficiently into subpockets on the protein. We hypothesized that replacing the benzimidazolone core with a quinolinone could maintain the important interactions for potent binding: a hydrogen-bond donation from the central nitrogen to Met51, with the acceptance of a hydrogen bond from the main chain N–H of Glu115 onto the quinolinone carbonyl, matching the hydrogen-bond pattern seen with the benzimidazolones. Additionally, a quinolinone scaffold would supply two new positions for vector exploration (C(3) and C(4)) that were distinct from the benzimidazolone.

**Figure 1:**
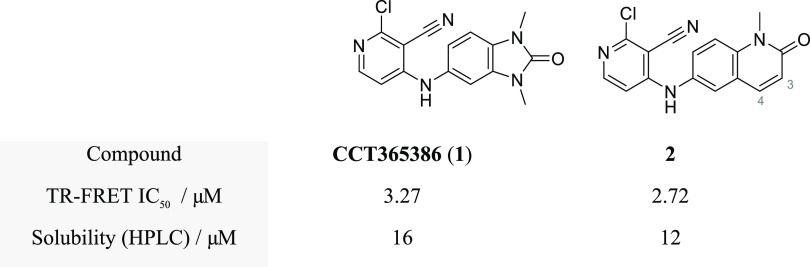
Rescaffolding of previous hit compound **1** gives the starting point for the quinolinone series, compound **2**.

We prepared quinolinone **2** as a proof-of-concept compound and found that it inhibited BCL6 with an IC_50_ of 2.7 μM in our biochemical time-resolved fluorescence energy transfer (TR-FRET) assay (Figure 1), representing a comparable starting point to its benzimidazolone counterpart **1**.

We report below the optimization of the quinolinone chemical series, which resulted in compound **25**, a BCL6 inhibitor with a submicromolar cellular activity.

## Chemistry

**Scheme 1:**
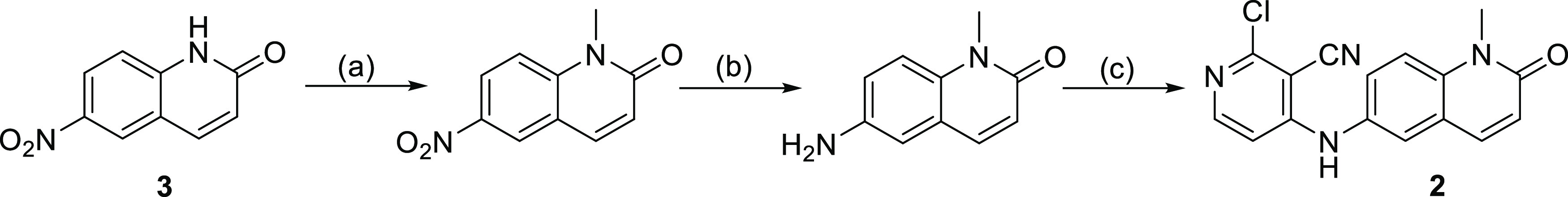
Synthesis of Compound **2** Reagents and conditions: (a) NaH, MeI, *N*,*N*-dimethylformamide (DMF), rt, 1 h; (b) SnCl_2_, ethanol, trifluoroethanol, 100 °C, 1 h; and (c) 2,4-dichloropyridine-3-carbonitrile, Et_3_N, DMF, 160 °C, 1 h.

The compounds described in this paper were generally constructed using sequences of well-known and characterized chemical reactions. The initial benzimidazolone hit **1** was prepared by a single-step nucleophilic aromatic substitution reaction from available building blocks,^17^ while its quinolinone counterpart **2** was synthesized in a three-step sequence from commercially available 6-nitroquinolin-2(1*H*)-one **3** (Scheme 1).

Substituted quinolin-2(1*H*)-one compounds were made from 4-chloro-1-methyl-6-nitroquinolin-2(1*H*)-one intermediate **5**, which was formed via acid-catalyzed hydrolysis and subsequent nitration of 2,4-dichloroquinoline **4**. Substitution in the 4-position was achieved through nucleophilic aromatic substitution, palladium-catalyzed Buchwald-type, or Suzuki couplings to give nitro-intermediates **6a–k**. Reduction of the nitro-intermediates **6a–k** was either achieved through reduction with tin chloride or transfer hydrogenation with Pd/C and ammonium formate to give aromatic amino compounds **7a–k**. The final step for this group of compounds is the nucleophilic substitution with 2,4-dichloropyridine-3-carbonitrile and triethylamine or diisopropylethylamine (DIPEA) as a base if needed to give compounds **8a–f** and **12a–e**.

**Scheme 2:**
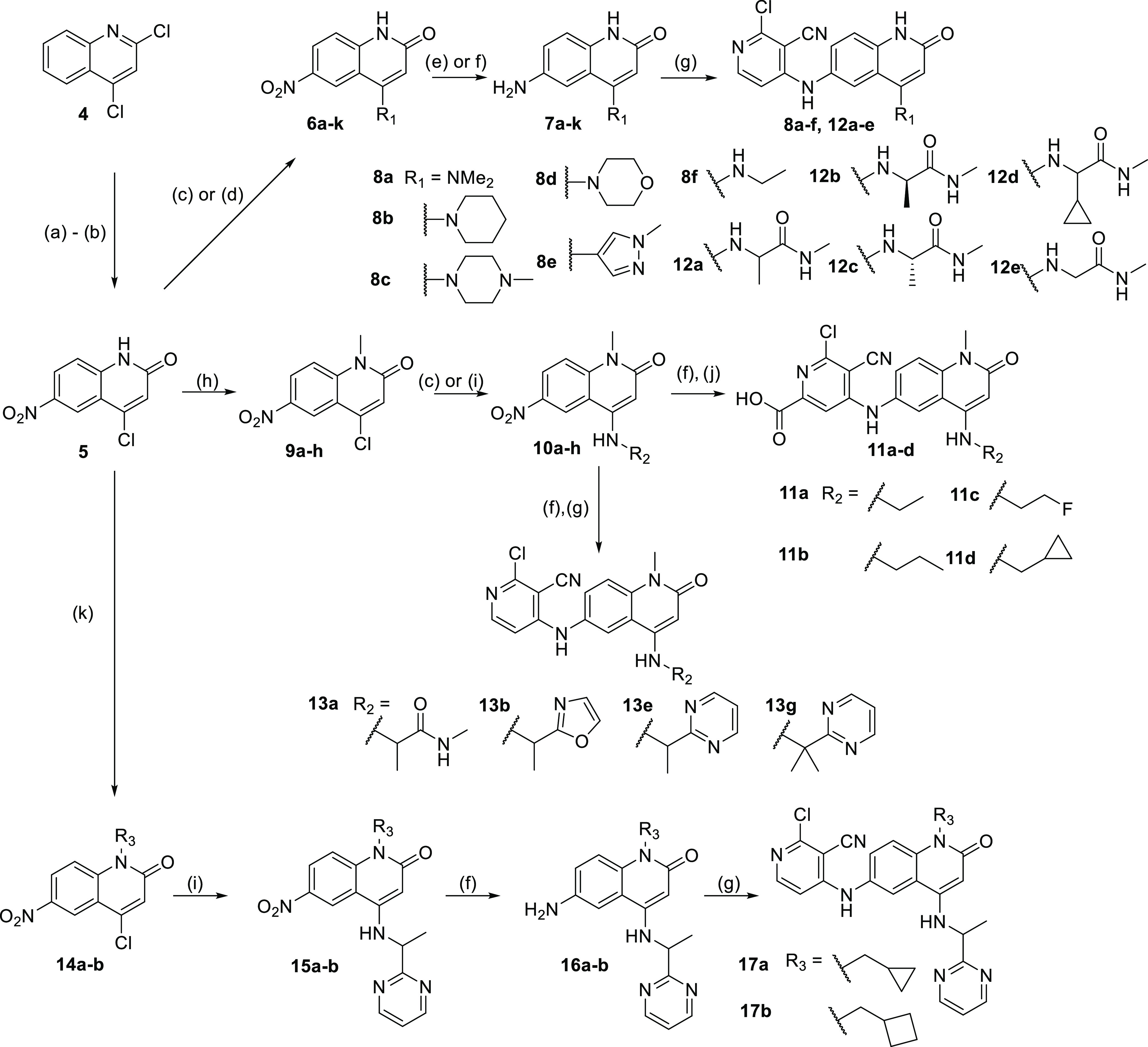
Synthesis of Quinolinone Compounds Shown in Tables 1–4 Reagents and conditions: a) 12M HCl, 1,4-dioxane, reflux, 18 h; b) H_2_SO_4_, HNO_3_, 0 °C, 1 h; c) requisite amine, NMP, 160 °C, up to 16 h; d) requisite boronate ester, Pd(PPh_3_)_4_, 2M aq Na_2_CO_3_ 140 °C, 1 h; e) SnCl_2_, ethanol, trifluoroethanol, 120 °C, 1 h; f) Pd/C (10% w/w), ammonium formate, ethanol or NMP, 60 °C, up to 4h; g) 2,4-dichloropyridine-3-carbonitrile, Et_3_N or DIPEA, 160 °C, 1 h; h) NaH (60% in mineral oil, DMF, MeI, rt, 90 min; i) requisite amine, Pd(OAc)_2_, Cs_2_CO_3_, rac-BINAP, PhMe, 120 °C, <4 h; j) 4,6-dichloro-5-cyano-pyridine-2-carboxylic acid, NMP, 100 °C, 2 h; k) Cs_2_CO_3_, bromomethylcyclopropane or bromomethylcyclobutane, DMF, rt, 1−2 d.

Quinolinone compounds with *N*^1^-alkylation were synthesized from 4-chloro-1-alkyl-6-nitroquinolin-2(1*H*)-one intermediate **5**. *N*-Alkylation was achieved through reaction with sodium hydride and iodomethane or through cesium carbonate and either (bromomethyl)cyclopropane or (bromomethyl)cyclobutane to give **9**, **14a**, and **14b**, respectively. Substitution in the 4-position of *N*^1^-methyl-quinolinone **9** was achieved through nucleophilic aromatic substitution or Buchwald-type couplings with primary amines. Reduction of these 4-substituted nitro-quinolinones **10a–h** was achieved through the use of tin chloride or by transfer hydrogenation with Pd/C and ammonium formate. Substitution with 1-pyrimidin-2-ylethanamine in the 4-position of *N*^1^-methylcyclopropyl and *N*^1^-methylcyclobutyl quinolinones **14a,b** was achieved through Buchwald-type couplings. The resulting nitro-quinolinones **15a,b** were reduced via transfer hydrogenation and coupled to 2,4-dichloropyridine-3-carbonitrile via nucleophilic aromatic substitution to give **16a,b**. The final step for all *N*^1^-substituted compounds was nucleophilic aromatic substitution with 4,6-dichloro-5-cyanopicolinic acid or 2,4-dichloropyridine-3-carbonitrile with DIPEA as a base if needed (Scheme 2).

**Scheme 3:**
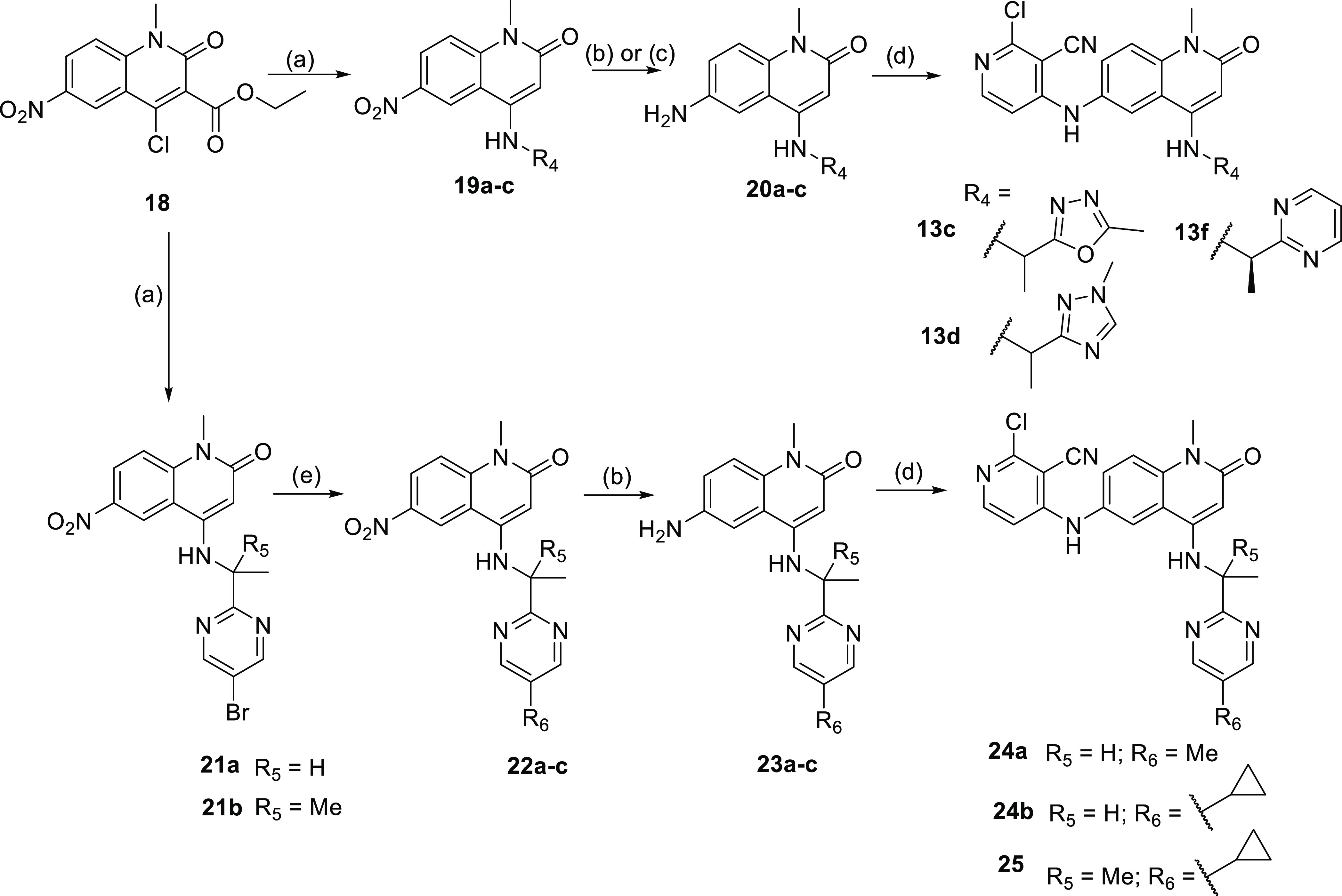
Synthesis of Additional Quinolinone Compounds Shown in Tables 4 and 5 Reagents and conditions: (a) requisite amine, DIPEA, 160 °C, 1 h, then LiCl, 160 °C, 1 h; (b) Pd/C (10% w/w), ammonium formate, ethanol, 60 °C, <4 h; (c) SnCl_2_, ethanol, trifluoroethanol, 120 °C, 1 h; (d) 2,4-dichloropyridine-3-carbonitrile, DIPEA, 160 °C, 1 h; and (e) requisite boronic acid, Pd(PPh_3_)_4_, 2 M aq. Na_2_CO_3_, DMF, 140 °C, <2 h.

Further *N*^1^-methyl compounds were synthesized from the ethyl-ester quinolinone intermediate **18**, which itself was synthesized in three steps from 5-nitro-isatoic anhydride. Substitution in the 4-position was achieved in two sequential steps: primary amines underwent a nucleophilic addition with DIPEA in THF or DMF followed by decarboxylation induced by LiCl or NaOH. Reduction of the nitro compounds **19a–c** was achieved through analogous means to other *N*^1^-methyl compounds. The extended pyrimidine compounds **24a**, **24b**, and **25** were derived from bromopyrimidines **21a** or **21b** through a Suzuki coupling with the requisite boronic acid. These nitro compounds **22a–c** were then reduced by transfer hydrogenation with Pd/C and ammonium formate. Similar to above, the final step for amino-quinolinones **20a–c** and **23a–c** was nucleophilic aromatic substitution with 2,4-dichloropyridine-3-carbonitrile with DIPEA as a base if needed (Scheme 3).

## Results and Discussion

**Figure 2:**
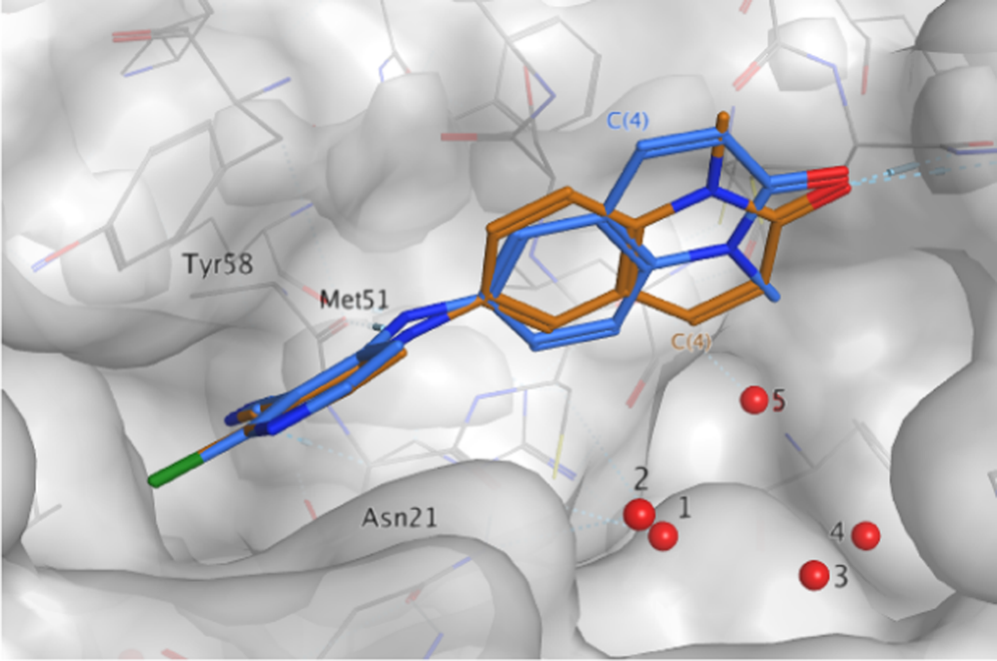
X-ray structure of **2** (PDB: 7OKE, 1.48 Å resolution) showing two fairly equally populated conformations: solvent-facing (blue, 32% occupancy*) and protein-facing (orange, 38% occupancy*). Conserved water molecules in the wider pocket are numbered 1–5. *Total occupancy of the compound is 70%.

To initiate the medicinal chemistry optimization and support the generation of further design hypotheses for the quinolinone core, we solved the X-ray crystal structure of **2** bound to the BCL6 BTB domain dimer. The structure revealed that **2** was bound to BCL6 in two different orientations with the C(4)H of the quinolinone either solvent-facing (blue) or protein-facing (orange) (Figure 2). For both orientations, we observed the key interactions previously reported for the benzimidazolone series. These included hydrogen bonds with the carbonyl of Met51 and the backbone NH of Glu115. The pyridine group of the compound was positioned in a cleft between Tyr58 and Asn21, forming a face-to-face stacking interaction with the side chain of Tyr58. The occupancies for both orientations in the crystal structure of **2** were similar at 32 and 38%, suggesting no preference for one of the two conformers.

Having validated the quinolinone core, our next aim was to improve the potency through growing from this relatively small molecule. We identified a subpocket below the quinolinone core that is part of the peptide-binding groove on the BCL6 BTB domain but not currently occupied by our ligands. Our aim was to increase potency by growing into this pocket. Importantly, the C(4)-position of the quinolinone in the protein-facing conformation seemed well positioned to extend into this pocket with a variety of substituents. However, the pocket was occupied by a network of five water molecules conserved in several in-house ligand-bound BCL6 structures. Growing into this pocket would inevitably perturb this water network, and this perturbation was likely to have an effect on the potency of new ligands. We suspected that the key challenge in improving potency by growing into this pocket was to discover substituents that not only engage in interactions with the protein but also interact with and perturb the water network in a productive way. Furthermore, we hypothesized that we can discover such substituents by probing the pocket by introducing a range of small substituents in the C(4)-position of the core. A representative set of carbon- and nitrogen-linked derivatives are summarized in Table 1. To increase solubility, we decreased lipophilicity by removing the substitution at the *N*^1^*-*position of the quinolinone core.

**Table 1:**
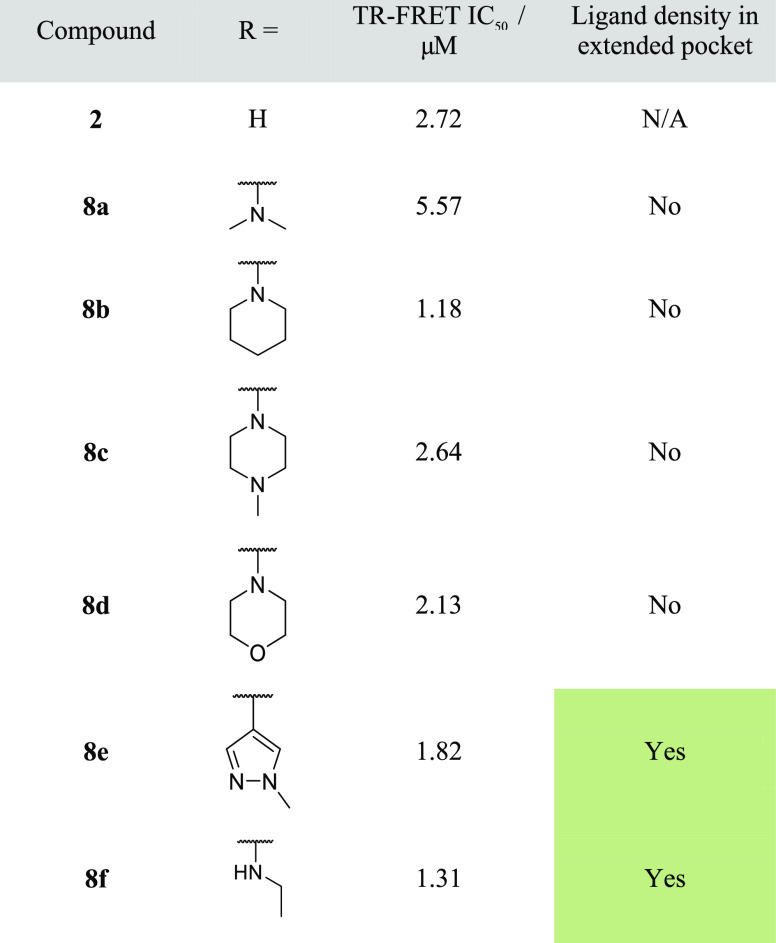
Rapid Geometric X-ray Screen of 4-Substituted Quinolinones

Disappointingly, this set of compounds **8a**–**8f** showed no increase in potency in our biochemical assay. Moreover, the structure–activity relationship (SAR) was flat with IC_50_s of 1.5–2.5 μM and hence did not provide any indications as to which substituents bind preferentially in the targeted pocket. Despite the flat SAR, we hypothesized that some of the compounds may still place the newly introduced substituents in the pocket. Furthermore, we speculated that these represent better starting points since the substituent can be modified further to derive additional interactions and more completely fill the extended pocket. Since our initial starting point bound in two conformations and the flat SAR did not reveal the binding preference, we progressed all compounds to crystallography. To rapidly confirm the binding mode of these compounds, we solved the BCL6 protein–ligand structures but initially did not model and refine the bound ligand. Instead, we just checked for the presence of ligand density and, in particular, for electron density in the extended pocket. Ligand density in the pocket is consistent with the compound bound in the pocket-facing conformation, whereas the presence of electron density for the core of the ligand, but not in the pocket, suggested that the compound bound in the solvent-facing conformation. This crystallographic analysis gave us an insight into the different binding modes of compounds with otherwise similar biochemical potency.

**Figure 3:**
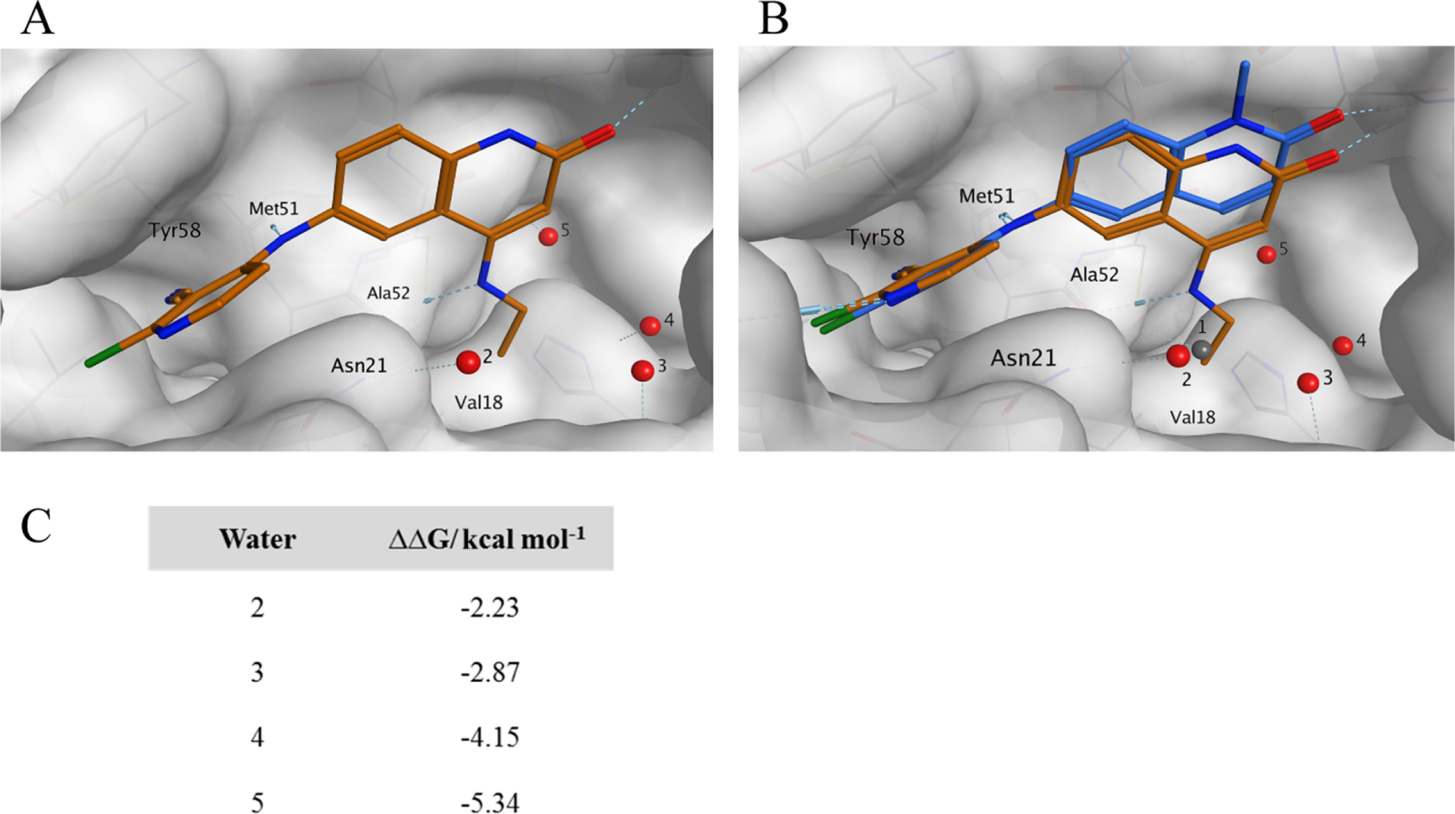
(A) X-ray structure of **8f** (PDB: 7OKH, resolution 1.52 Å) showing interactions with the protein and positions of the remaining water molecules 2–5. Water molecule 1 present in the X-ray structure of **2** (Figure 2) has been displaced and is no longer present. (B) X-ray structure of **2** (PDB: 7OKE) showing positions of water molecules 1–5, overlaid with the chemical structure of **8f** (PDB: 7OKH, ligand only), demonstrating the shift toward the extended pocket and the colocation of the ethyl moiety with the position of water 1 (gray). (C) SZMAP water analysis showing ΔΔ*G* of the four conserved water molecules in the extended pocket of **8f**.

This rapid X-ray analysis showed that the majority of the compounds in Table 1 had no density in the extended pocket, thus most likely binding in a single, solvent-facing orientation, contrary to what we observed with unsubstituted **2**. Secondary amines, including dimethylamine **8a**, piperidine **8b**, piperazine **8c**, and morpholine **8d**, were all found to bind solely in the solvent-facing conformation. Because the second pocket-facing conformation was not observed, we suggest that the substituents did not interact favorably with the extended pocket despite their similar potency consistent with the flat SAR observed. There were two compounds for which ligand density was observed in the extended pocket: carbon-linked pyrazole **8e** and ethylamine **8f**. To understand how these compounds bound, we modeled the compounds and fully refined their structures. The pyrazole compound **8e** was shown to bind in both orientations with comparable occupancies, in a similar fashion to **2** (see the Supporting Information, Figure S1). Interestingly, ethylamine-substituted quinolinone **8f** bound solely orientated with the 4-substituent in the extended pocket. A detailed analysis of the **8f**-bound BCL6 structure (Figure 3) revealed a number of interactions that stabilize the pocket-facing binding mode: first of all, the newly introduced NH of the substituent engages in an additional hydrogen bond with the main chain carbonyl of Ala52. Second, the substituent displaces one of the conserved water molecules (water 1), and its terminal methyl group occupies a small hydrophobic pocket near the side chain of Val18. The structure also offers a possible explanation as to why the potency was not improved despite these additional interactions, as the geometry of hydrogen bond between the substituent NH and Ala52 is suboptimal with a distance of 3.01 Å and an angle of 36° between the NH and H to O vectors. Moreover, the scaffold is shifted compared to **2**, thus potentially leading to less favorable interactions of the core with binding pocket residues (Figure 3B).

Compound **8f** was bound in the pocket-facing orientation, and we next explored it as a chemical starting point. We investigated if significant potency gains could be achieved by further substituting the ethyl chain to interact with the protein and the water network. Inspection of the crystal structure revealed that small substitutions of the terminal methyl group should be tolerated and we designed and synthesized a small set of **8f** analogues (Table 2). The increased lipophilicity of these analogues prompted us to include a solubilizing carboxylic acid group on the pyridine side of the molecule. At the same time, the *N*^1^-position was capped with a methyl group. We knew from previous work^17^ that these modifications only had a minor effect on potency.

**Table 2:**
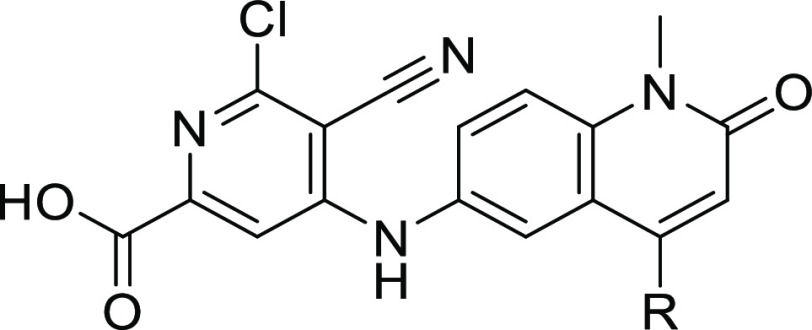

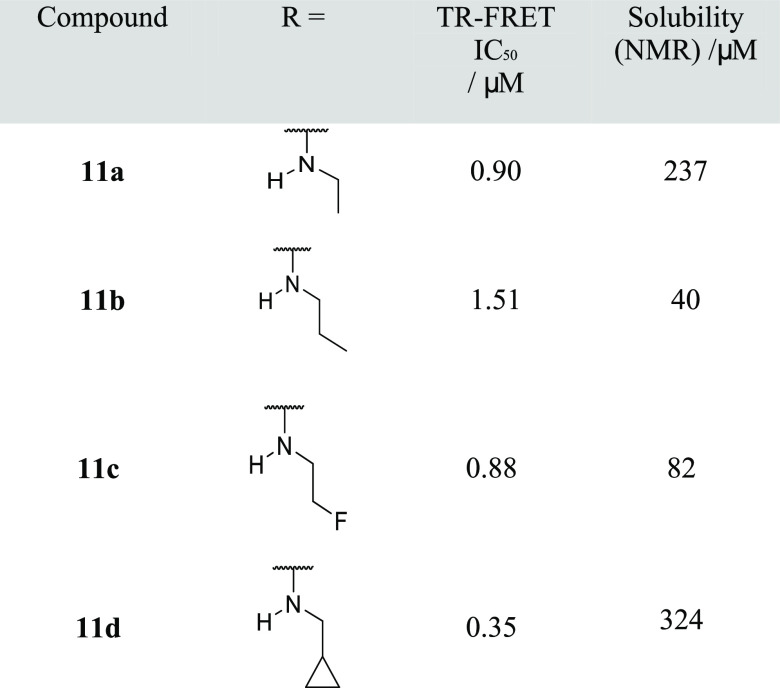
Structure–Activity Relationship of Small Alkylamino Substituents

As anticipated, compound **11a**, which features the same ethylamine substituent as **8f** but differed from it by having a carboxylic acid group on the pyridine ring and a methyl group in the *N*^1^-position, showed comparable potency. Compounds **11b** and **11c** in which the ethyl group was extended by a methyl- or fluoro-substituent, respectively, showed comparable biochemical activity. The methylcyclopropyl analogue **11d** brought a threefold improvement in biochemical potency in our assay. The potency increase suggested that **11d** may bind in the pocket-facing orientation with the cyclopropyl group potentially accommodated next to Val18, engaging in additional hydrophobic interactions. Unfortunately, we were not able to confirm the binding mode of **11d** by crystallography.

Having identified that small modifications to **8f** influenced potency, our next aim was to improve protein–ligand contacts by adding additional functional groups to the carbon atoms of the alkyl chain, particularly to the methylene group adjacent to the nitrogen in **8f**. Any modifications to this part of the molecule would necessarily lead to further perturbations of the other water molecules in the pocket. To better understand the role of the water network on ligand binding, we conducted a computational analysis using Openeye SZMAP software.^19^

This SZMAP analysis estimates a difference or change in relative free energy (ΔΔ*G*) when a water molecule is replaced by a hydrophobic probe at the coordinates of a water molecule seen in an X-ray structure. A positive predicted free-energy difference (ΔΔ*G*) indicates that displacing a water with a hydrophobic group at this location is favored, a negative that this is disfavored. In the case of ethylamine compound **8f**, the remaining waters are all calculated to have a negative free-energy difference (Figure 3C). This indicates that displacing these water molecules will only be favorable for potency if they are replaced by polar groups that mimic the interactions that the water molecules form with each other and the protein.

Given that our calculations predicted that it would not be favorable to replace the remaining four water molecules with lipophilic groups, we next prepared a range of derivatives that featured polar substituents at the methylene group. While most of these did not show significant potency improvement (data not shown), adding an *N*-methyl amide group onto the ethylamine to yield racemic **12a** gave a submicromolar compound in our TR-FRET assay (Table 3).

**Table 3:**
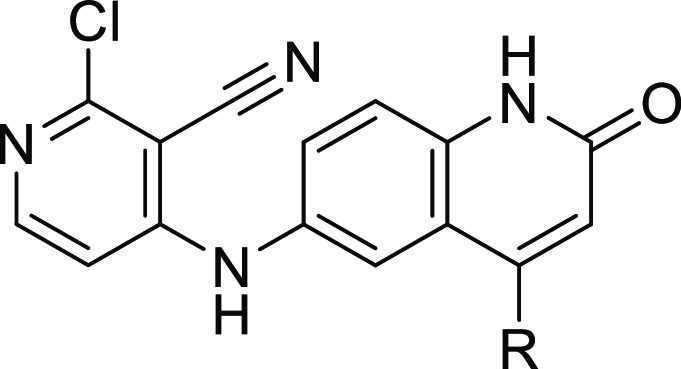

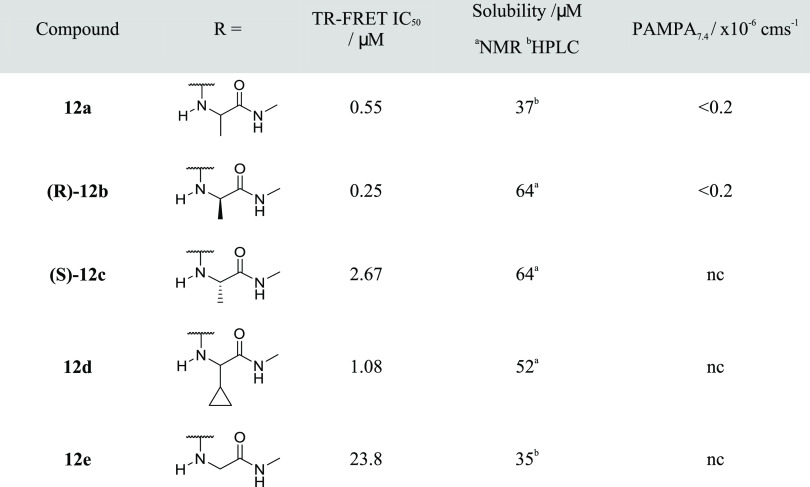
Structure–Activity Relationships for Polar Amide Groups in the Extended Pocket^b^

a Solubility measurement by NMR assay.

b Solubility measurement by high-performance liquid chromatography (HPLC). nc, not conducted.

**Figure 4:**
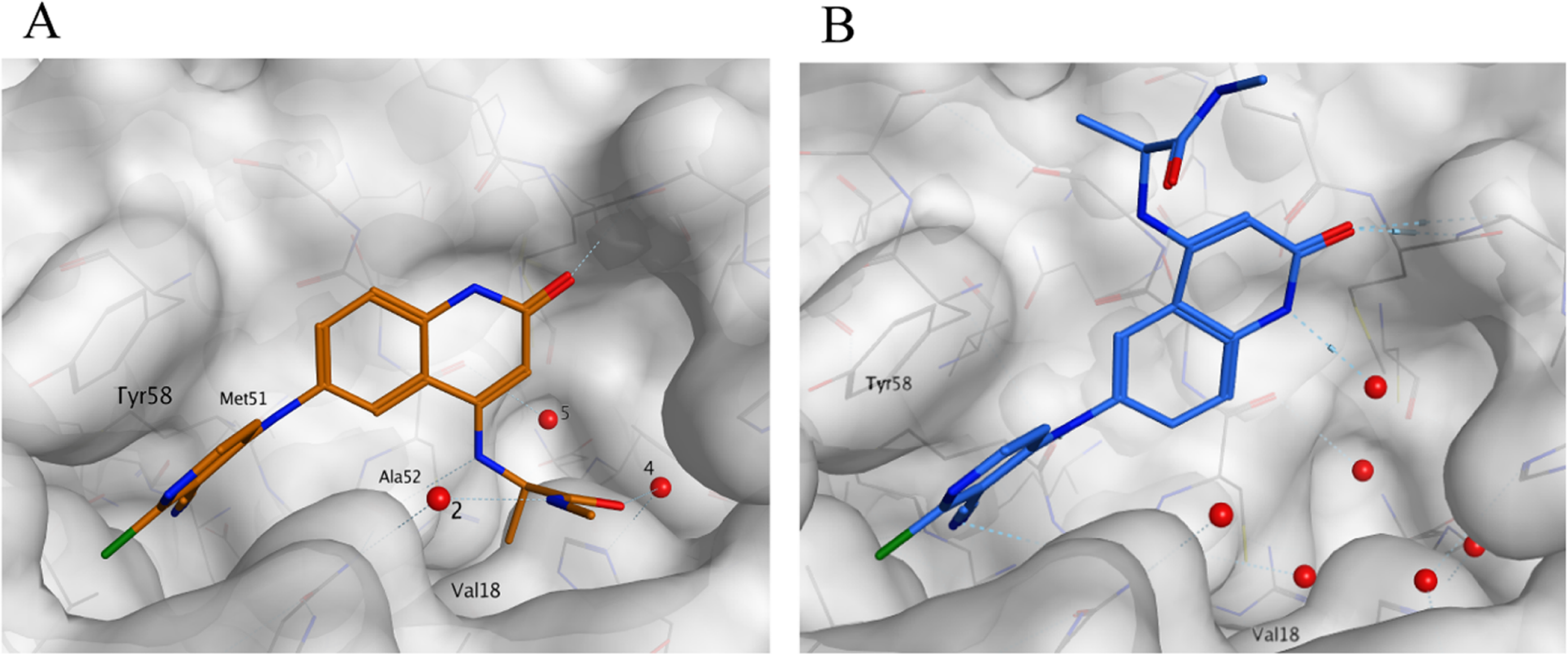
(A) X-ray structure of pocket-facing enantiomer (*R*)-ethyl amide **12b** (PDB: 7OKI, resolution 1.61Å). (B) X-ray structure of (*S*)-enantiomer **12c** (PDB: 7OKJ, resolution 1.43 Å) adopting a solvent-facing binding mode. The structure of **12c** contained a 34% occupancy of opposite enantiomer due to the presence of a small percentage of **12b** in the sample, which was omitted from this figure for clarity.

As amide **12a** was a racemic mixture, we next synthesized the two enantiomers. They showed different potency, with the (*R*)-enantiomer **12b** showing an IC_50_ of 0.25 μM, which translates as a ninefold increase for the introduction of the amide group. The (*S*)-enantiomer **12c** was significantly less potent. To rationalize this SAR and derive design hypotheses, we solved the crystal structures of both compounds. Consistent with its lower activity, the (*S*)-enantiomer bound in the solvent-facing orientation with the amide substituent pointing toward the bulk solvent with no interaction created between the quinolinone C(4)-substituent and the protein. The structure of the (*R*)-enantiomer proved to be more interesting. It bound in the pocket-facing orientation, and the ethylamine portion of the molecule was situated in the same position as observed with **8f**, displacing water 1. Not surprisingly, introducing the amide substituent substantially perturbed the water network. It displaced water 3, positioning the carbonyl oxygen atom in this position. The carbonyl function also engaged in a polar interaction with water 4, with the distance (water to carbonyl oxygen of 2.73 Å) and angles (C=O···O angle of 124.6° with the water oxygen in the plane of the carbonyl) consistent with a strong hydrogen bond. Finally, the NH moiety of the amide acted as a donor and engaged in a hydrogen bond (amide nitrogen to water oxygen distance of 2.83 Å) with water 2 (Figure 4).

We introduced the same amide group to cyclopropyl-substituted **11d** resulting in **12d** but in this case observed a loss of potency (IC_50_ = 1.08 μM), suggesting that the SAR for the ethyl and the cyclopropylmethyl substituents is different.

Another revealing observation was that the removal of the terminal methyl group from **12a** led to a 30-fold loss of activity (**12e**). This result highlights the critical importance of this methyl group to anchor the substituent in the pocket, likely by displacing water 1 and engaging in hydrophobic interaction with the side chain of Val18. Consistent with its poor potency, the crystal structure of **12e** revealed that it bound in the solvent-facing orientation, with the C4 substituent seen in multiple conformations, highlighting its poor stabilization (see Supplementary Figure S2).

With the (*R*)-ethyl amide **12b** achieving a ninefold improvement in potency, we next profiled the compound further. Not surprisingly, given that the compound featured four hydrogen-bond donors, we observed no measurable passive permeability in a PAMPA assay (Table 3). To improve permeation, we first removed one hydrogen-bond donor by capping the quinolinone *N*^1^-position with a methyl group (**13a**). While this modification maintained potency, **13a** still showed no passive permeability (PAMPA Pe <0.2 × 10^–6^ cm s^–1^), likely due to the combination of three remaining hydrogen-bond donors and relatively low lipophilicity (*A* Log *P* = 1.4; Table 4).

**Table 4:**
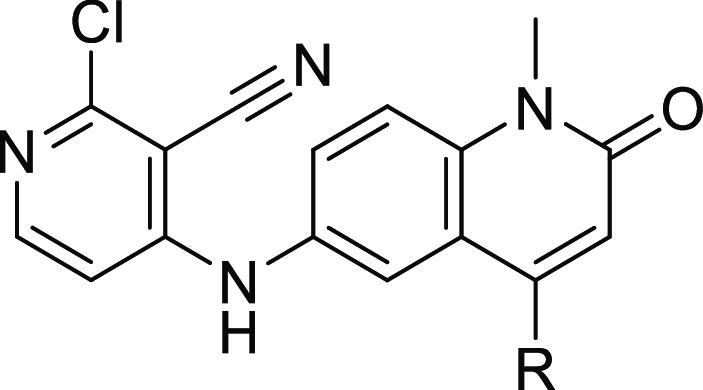

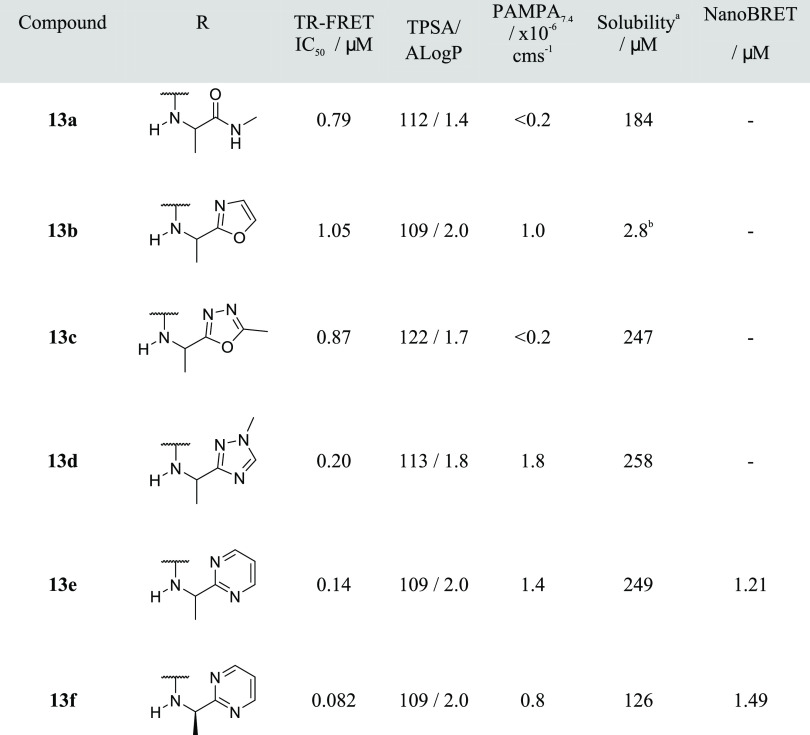
Structure–Activity Relationships of Replacing the Amide Moiety with a Heteroaromatic Isostere^b^

a Solubility measurement by NMR assay unless otherwise stated.

b Solubility measurement by HPLC assay.

Consequently, we turned to amide binding isosteres, particularly heteroaromatic groups.^20,21^ To lower the number of hydrogen-bond donors, we hypothesized that water 2 could reorientate itself to act as a hydrogen-bond donor toward our inhibitor instead of an acceptor, allowing us to replace the NH of the amide substituent with an acceptor moiety. We prepared a series of compounds to test this hypothesis (Table 4). Oxazole **13b** did maintain potency, suggesting that water 2 had indeed reoriented itself. **13b** also showed a slight improvement in passive diffusion (1.0 × 10^–6^ cm s^–1^). 1,3,4-Oxadiazole **13c** also showed comparable potency to amide **13a**. Interestingly, N-Me triazole **13d** brought a fourfold improvement in potency but no measurable improvement in passive permeability, suggesting that the triazole introduced too much polarity. Gratifyingly, the pyrimidine analogue **13e** provided a significant breakthrough. It was not only sevenfold more potent but also had a measurable permeability of 1.4 × 10^–6^ cm s^–1^ in our PAMPA assay. This enabled us to progress into the cellular assay. In contrast to the TR-FRET assay, which uses the BTB domain of BCL6 and a short peptide from the corepressor, the NanoBRET assay uses full-length BCL6 and corepressor. Pleasingly, compound **13e** showed measurable activity (1.21 μM), which we looked to optimize by further increasing binding affinity using a structure-based design.

**Figure 5:**
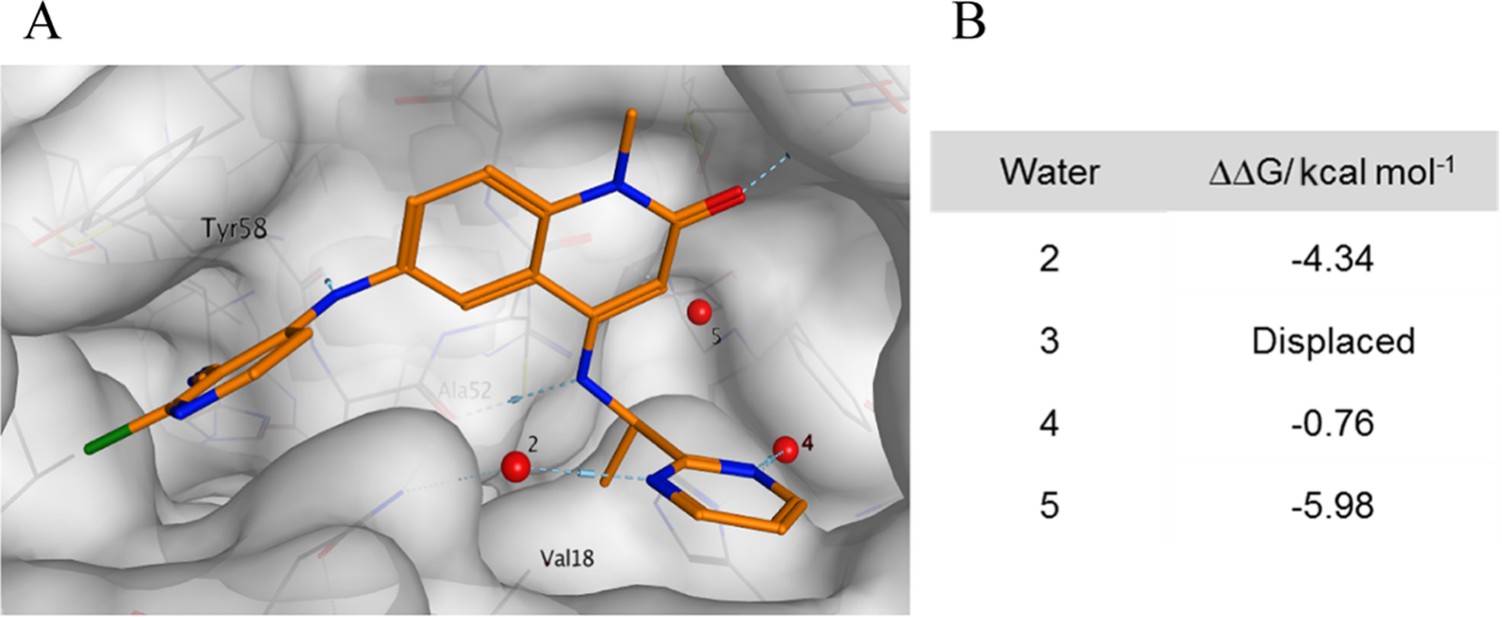
(A) X-ray structure of **13e** (PDB: 7OKL, resolution 1.25 Å). (B) SZMAP analysis of the remaining water molecules from the conserved water molecule set in the extended pocket.

We solved the X-ray structure of pyrimidine **13e**. As hypothesized, the pyrimidine adopted the position of the amide group in **12a**, displacing water 3 while acting as a double hydrogen-bond acceptor from waters 2 and 4 with water positions consistent with interactions with the in-plane lone pairs on the pyrimidine nitrogens (N···H–O distance 2.8 Å). The ethylamine portion of the substituent maintained its position, with the terminal methyl group displacing water 1 and forming lipophilic interactions with Val18 (Figure 5). Despite using racemic material for crystallography, we only observed the (*R*)-enantiomer in the structure. We therefore synthesized the single (*R*)-enantiomer **13f**, which indeed showed the expected minor improvement of biochemical potency (IC_50_ = 0.082 μM) (Table 4).

With pyrimidine **13f**, we had improved biochemical potency and achieved a measurable cellular activity. However, despite the significant improvement, the passive permeability was still <1 × 10^–6^ cm s^–1^. To increase the permeability of **13f**, we aimed to incorporate more lipophilicity into the molecule. Inspection of the crystal structure led to different hypotheses as to where lipophilic substituents could be tolerated. Modification at the *N*^1^-position with lipophilic substituents yielded *N*^1^-methylcyclopropyl and *N*^1^-methylcyclobutyl compounds **17a** and **17b**. These confirmed that increasing the lipophilicity can indeed lead to increased permeation (Table 5). Unfortunately, these compounds showed a threefold higher IC_50_ and we did not explore modification of the *N*^1^-position further. Another position that we exploited was the 5-position of the pyrimidine ring. Extension of the pyrimidine ring with methyl and cyclopropyl groups toward the front of the pocket gave **24a** and **24b**, which maintained a TR-FRET potency of 0.073 and 0.054 μM, respectively (Table 5), and showed improved permeability as measured by PAMPA.

**Table 5:**
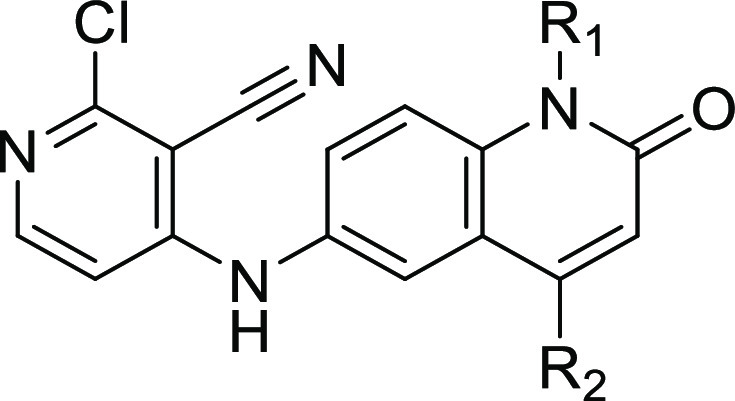

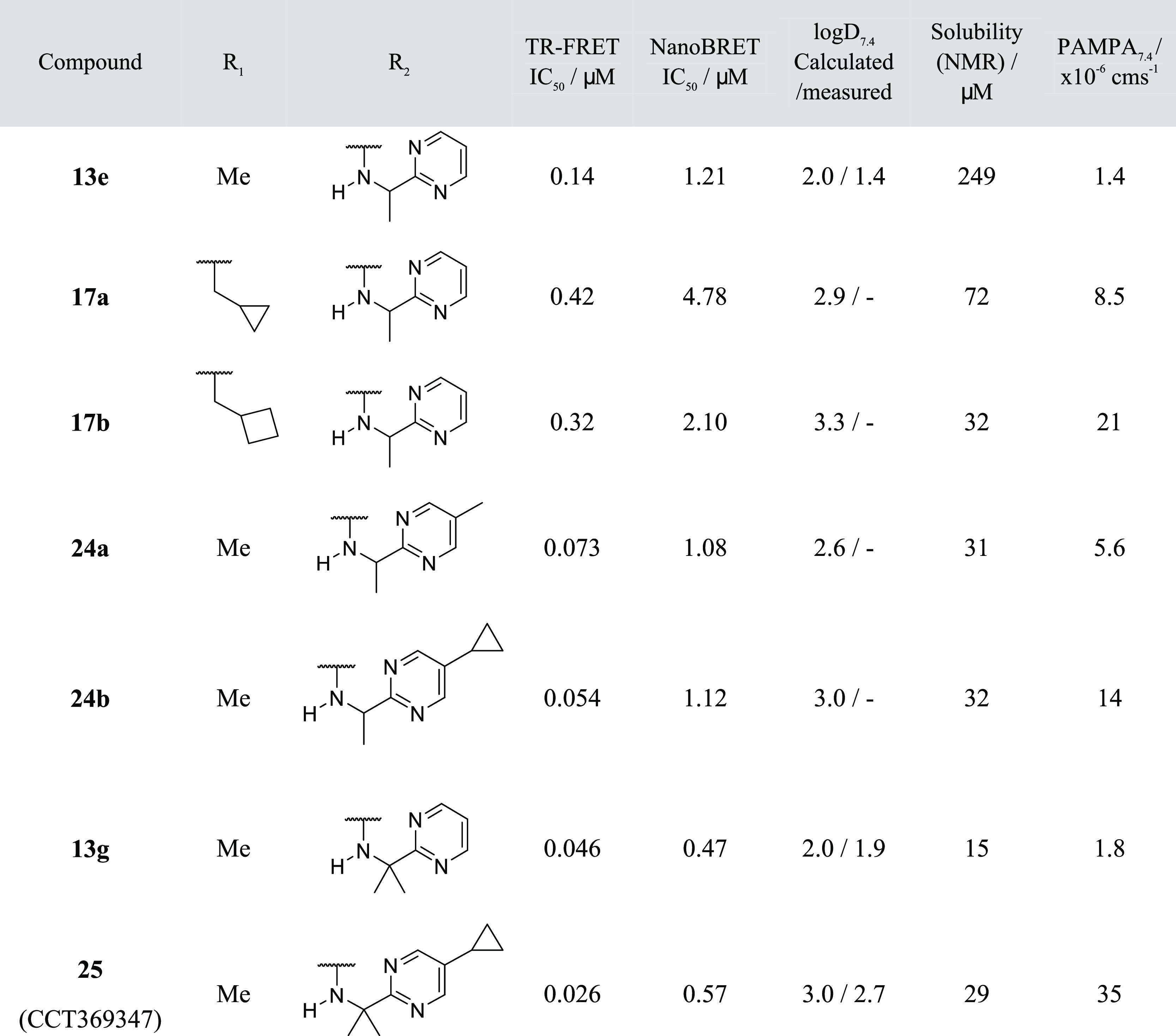
Structure–Activity Relationships of Lipophilic Additions to Pyrimidine Compound **13e**

**Figure 6:**
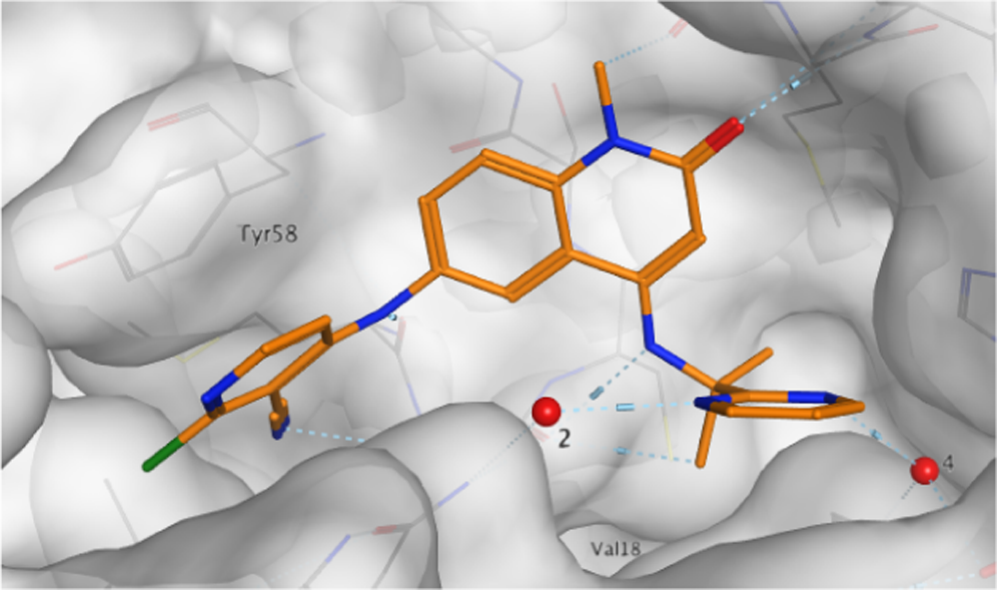
X-ray structure of **13g** (PDB: 7OKM, resolution 1.48 Å). “Water 5” has been displaced.

An additional vector that we considered for improving permeability was branching off the chiral carbon that bears the terminal methyl group and the pyrimidine ring. However, replacing the remaining hydrogen would inevitably displace water 5. We thus conducted an SZMAP analysis to gauge if replacing water 5 in the cocrystal structure of **13e** with a hydrophobic group could be favorable. Interestingly, this analysis suggested that water 5 was tightly bound, with a ΔΔ*G* of −5.98 kcal mol^–1^, which would mean that replacing it with a methyl group would not be expected to be energetically favorable for potency (Figure 5). We nevertheless prepared the dimethyl pyrimidinylmethanamine analogue **13g**. Surprisingly, given the outcome of the SZMAP analysis, we observed an IC_50_ of 0.046 μM in our TR-FRET assay, a small (twofold) gain in potency compared to the (*R*)-enantiomer **13f**. We progressed this compound to crystallography. The X-ray structure showed that the newly introduced methyl group indeed replaced water 5 and otherwise bound in an analogous fashion to **13e**, displacing waters 1 and 3. Distances from pyrimidine nitrogen to waters 2 (2.83 Å) and 4 (2.88 Å) are consistent with hydrogen bonding, although the position of water 4 is no longer coplanar, potentially weakening this interaction (Figure 6).

**Figure 7:**
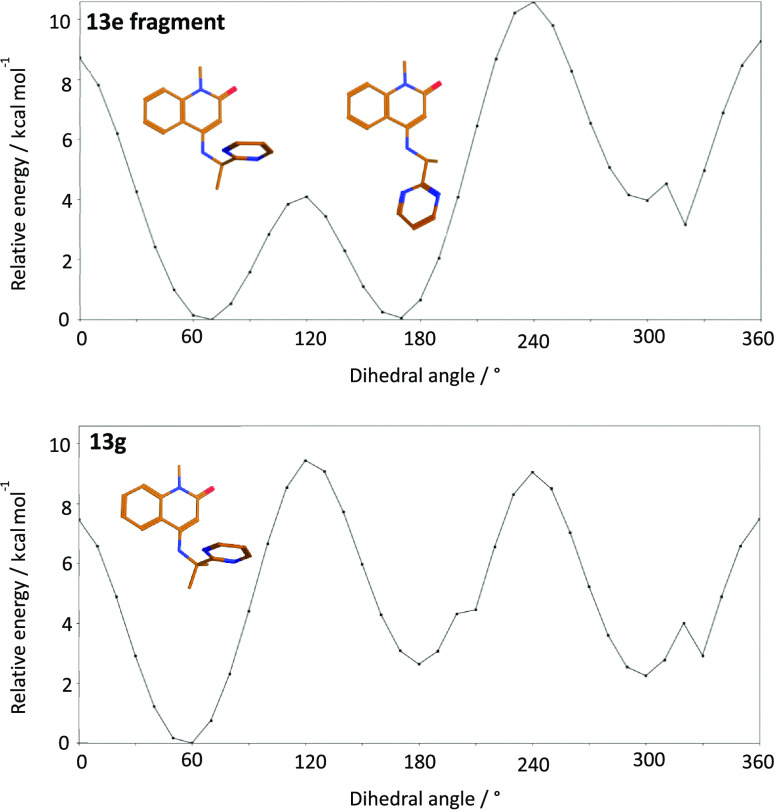
Macromodel dihedral angle calculated free-energy scan for the quinolinone fragments of **13e** and **13g**, showing a single minimum for the more potent **13g** near the bioactive conformation.

The observation that replacing water 5 in the complex of **13e** with BCL6 was tolerated despite being predicted to be unfavorable by the SZMAP analysis was interesting. We hypothesized that the incorporation of an additional methyl group had an effect on the unbound conformation of **13g** and steered it toward the bioactive conformation. Furthermore, we speculated that this effect offsets any loss in potency due to replacing water 5. To investigate this hypothesis, we conducted a computational dihedral angle analysis on the relevant fragments (Figure 7) of both the mono-methyl and dimethyl pyrimidine compounds **13e** and **13g** in the solution phase using Macromodel software. We calculated the relative free strain energy for different dihedral angles between the quinolinone C(4)–NH bond and the C(α)-pyrimidine bond (Figure 7). These revealed differences in the conformational preferences of this dihedral angle. Whereas two minima with equivalent energy were calculated for the **13e** fragment at 60–65 and 170–175°, the dimethyl analogue **13g** was only predicted to have one at 55–60°. The minima at 60–65° for **13e** and 55–60° for **13g** are close to those seen in the crystal structure (66.59 and 53.85°, respectively). This analysis suggests that introducing the methyl group to yield **13g** preorganizes the torsion angle toward a single conformation close to the bioactive conformation. This analysis is consistent with our hypothesis that the conformation preference introduced by the methyl group at least partially compensates for loss due to replacing water 5. Moreover, we observed that the torsion angle slightly differed between **13e** and **13g** and it is possible that the angle observed for **13g** leads to improved interaction of the pyrimidine ring and thus also contributes to off-setting the penalty caused by replacing a relatively stable water with a lipophilic moiety.

Compound **13g** proved to be an effective inhibitor in our cellular NanoBRET assay (IC_50_ = 0.47μM, *n* = 3). However, while the lipophilicity translated into a slight increase in cellular activity, its PAMPA permeability was still relatively low. To optimize passive permeability further, we combined the lipophilic substitution of the chiral carbon (**13g**) and the extended pyrimidine (**24b**). Gratifyingly, the increased lipophilicity of the resulting compound **25** enabled satisfactory permeability (35 × 10^–6^ cm s^–1^) for the first time in this series while maintaining an acceptable solubility of **25** (30 μM). Biochemical and cellular potency was maintained, and binding affinity to the BCL6 BTB domain was also confirmed by SPR (**25** TR-FRET pIC_50_ 7.58, SPR p*K*_d_ 6.63).

**Figure 8:**
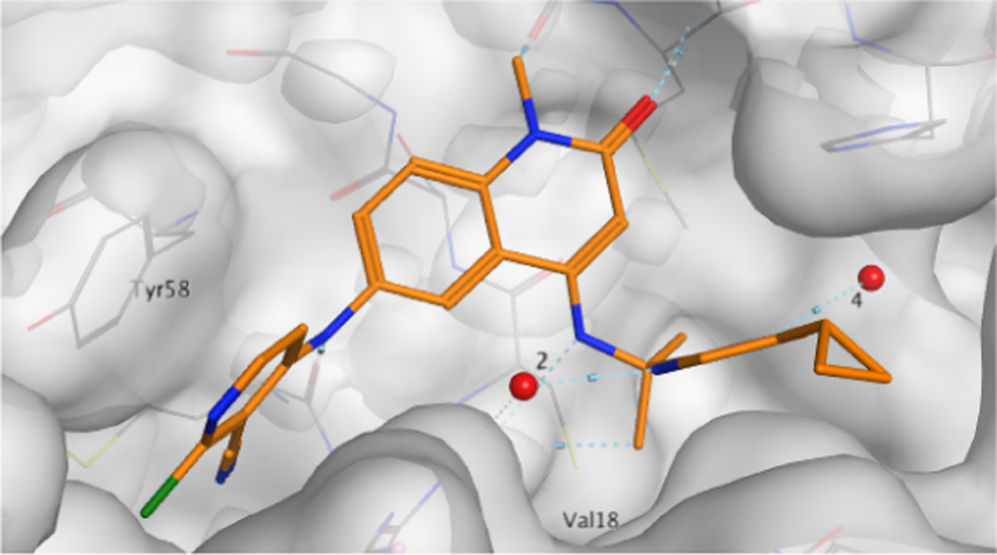
X-ray structure of **25** (PDB: 7OKD, resolution 1.94 Å).

The X-ray structure of **25** (Figure 8) shows that the core scaffold and the pyrimidine substituent bind very similarly to **13g**: the ligand displaced waters 1, 3, and 5 from the extended pocket and the pyrimidine showed interactions with adjacent water molecules including 2 (2.75 Å) and 4 (2.96 Å). In addition, the cyclopropyl group of **25** is shown to fold down over the edge of the pocket, providing additional hydrophobic contacts.

To further assess the potential of this series, we carried out additional profiling. We conducted 14 day viability assays in a panel of BCL6-positive lymphoma cell lines (OCI-Ly1, SU-DHL-4, and SU-DHL-6) and in BCL6-low or negative cell lines (OCI-Ly3, MM.1S) to give an indication of specificity. As expected based on the NanoBRET data, we observed GI50 value ranging from 1 to 4 μM in the BCL6-dependent cell lines and weaker activity in those cell lines with low BCL6 expression (Table 6 and Supplementary Figure S4). Although this data is encouraging, with **25** fulfilling our requirements for an early lead compound, clearly more potent inhibitors of the BCL6:corepressor protein–protein interaction are required to enable the testing of the therapeutic hypothesis in vivo. Nevertheless, to assess whether this series had the potential for further optimization and identify potential issues, we then analyzed *in vivo* pharmacokinetic (PK) parameters in a female Balb\c mouse study (Balb\cAnNCrl, ex. Charles River), dosing at 1 mg kg^–1^ i.v. (*n* = 3) and 5 mg kg^–1^ p.o. (*n* = 3). All experiments were carried out according to the UKCC guidelines for animal experimentation, and no toxicity was observed. **25** (CCT369347) demonstrated moderate clearance (CL 24 mL min^–1^ kg^–1^) with a mean oral bioavailability of 29%. Protein binding measurements using equilibrium dialysis showed that the compound is 1.3% free in mouse plasma.

**Table 6:** Antiproliferative Activity of Compound **25** after 14 Days of Treatment

	BCL6-high			BCL6-low	
compound	OCI-LY1 GI_50_/μM	SU-DHL-4 GI_50_/μM	SU-DHL-6 GI_50_/μM	OCI-Ly3 GI_50_/μM	MM.1S GI_50_/μM
**25**	1.68	3.45	2.55	>10	>10

## Conclusions

We report the rescaffolding of benzimidazolone CCT365386,^17^**1**, to identify quinolinone **2** and the optimization of this compound by growing the core into an extended subpocket of the corepressor binding domain. This subpocket was occupied by a set of five water molecules. A key challenge was thus to discover core substitutions that not only addressed and efficiently filled the subpocket but also perturbed the tightly bound water network in a productive, potency-enhancing fashion. We thus decided to probe the pocket through systematic chemical modifications. However, crystal structures revealed that the starting core was able to bind in two orientations. In cases where newly introduced substituents did not have favorable interactions with the pocket, the core could simply flip around, pointing this substituent toward solvent without losing potency. It was thus not apparent from the initial SAR if derivatives indeed extended into the extended pocket.

We solved this challenge by subjecting several derivatives to a rapid crystallographic analysis. This analysis revealed that only one compound (**8f**) bound in a single conformation with its C(4) substituent extended into the subpocket. All other compounds bound either in the alternative or in both binding orientations. **8f** provided critical information on how the pocket can be stably occupied through forming an additional hydrogen bond with Met51, hydrophobic interactions with the side chain of Val18, and displacing one water molecule. At this stage, we used SZMAP to analyze the network of four water molecules remaining in the subpocket. This analysis predicted that all four water molecules were stably bound, suggesting that simply displacing them with lipophilic groups was unlikely to be beneficial for potency. Starting from **8f**, we hence introduced polar moieties to probe the subpocket and the water network. Amide **12a** provided a breakthrough and was our first submicromolar compound. X-ray analysis showed that the newly introduced amide replaced an additional water molecule and critically formed hydrogen bonds with two additional water molecules.

While **12a** represented a step in the right direction, the introduction of the primary amide function rendered the compounds too polar for satisfactory permeation and hence for BCL6 inhibition in a cellular context. We achieved another breakthrough by replacing the amide with a pyrimidine moiety. This modification led to compound **13e**, which was significantly more potent in our TR-FRET biochemical assay and with significantly improved albeit still low permeability, enabling activity in the cellular NanoBRET assay.

As the remaining H-bond donors in the molecule were all required for activity, we focused on increasing lipophilicity to further improve permeability. Addition of a second alkyl group α to the pyrimidine displaced a water molecule and increased log *D*_7.4_ by 0.5 log units. This displacement was predicted by SZMAP to be detrimental to potency; however, we instead observed a small potency improvement consistent with the increase in lipophilicity. We speculated that the additional methyl group may also stabilize a bioactive conformation; simple conformational analysis suggested that this could indeed be a contributing factor. These observations highlight that deceptively simple SAR results may be governed by multiple factors that can compensate for each other. Continuing our efforts to improve permeability further, we then appended a cyclopropyl group to the pyrimidine. This modification increases log *D*_7.4_ from 1.9 to 2.7, resulting in a 20-fold improvement of PAMPA permeability.

Overall, our work thus highlights the critical role of water molecules in binding events. **25**, in particular, shows the need to find productive ways to perturb networks of tightly bound water molecules. Of the five water molecules present in the unoccupied subpocket, three are replaced by **25**, two by hydrophobic groups and one through a polar moiety. Equally importantly, **25** engages in hydrogen bonds with the two remaining water molecules in the subpocket, thus contributing to the increased potency.

**Figure 9:**
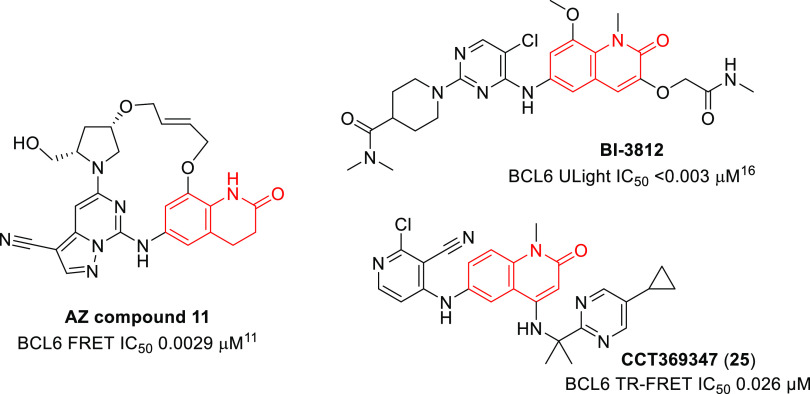
Structures of published BCL6 inhibitors showing different optimizations from quinolinone or dihydroquinolinone hit matter.

It is interesting to note the comparison between published BCL6 inhibitors from our own work and that from Boehringer-Ingelheim^16^ and AstraZeneca.^11^ All three groups independently identified quinolinone or dihydroquinolinone hits, with similar binding modes, but gained potency by different chemical strategies—branching from the C3-position, from the C4-position, or by macrocyclization. This highlights both the tractability of the BCL6-corepressor PPI to small molecule intervention and the creativity of different teams of medicinal chemists in finding multiple distinct and novel solutions to the same problem (Figure 9).

Because of the overall favorable profile of **25**, which inhibits BCL6 in a cellular assay with a submicromolar IC_50_ and shows promising early PK, we nominated this compound as a lead.

## Experimental Section

All *in vivo* experiments were carried out according to the U.K. guidelines for animal experimentation.

### General Synthetic Information

All anhydrous solvents and reagents were obtained from commercial suppliers and used without further purification. Evaporation of solvent was carried out using a rotary evaporator under reduced pressure at a bath temperature of up to 60 °C. Flash column chromatography was carried out using a Biotage purification system using SNAP KP-Sil cartridges or on reverse-phase mode using SNAP Ultra C18 cartridges. HPLC purification was carried out on an Agilent 6120 MS-Prep LC using an ACE 5 C18-PFP 250 mm × 21.2 mm column using a 15 min gradient of water:methanol (both modified with 0.1% formic acid)—for example, 10–100, 40–100, 60–100, or 55–80% at a flow rate of 20 mL per minute. Microwave-assisted reactions were carried out using a Biotage Initiator microwave system. Final compounds were purified to ≥95% purity. Product yields are quoted as (mass, % yield for the final step unless stated). NMR data was collected on a Bruker Avance 500 spectrometer equipped with a 5 mm BBO/QNP probe or on a Bruker Avance Neo 600 spectrometer equipped with a 5 mm TCI Cryo-Probe. NMR data is presented in the form of chemical shift δ (multiplicity, coupling constants, integration) for major diagnostic protons, given in parts per million (ppm) relative to tetramethylsilane (TMS), referenced to the internal deuterated solvent. Where “2 min ToF” is indicated, liquid chromatography–mass spectrometry (LC/MS) and high-resolution mass spectrometry (HRMS) analyses were performed on an Agilent 1200 series HPLC and a diode array detector coupled to a 6210 time-of-flight mass spectrometer with a dual multimode APCI/ESI source. Analytical separation was carried out at 40 °C on a Merck Chromolith Flash column (RP-18e, 25 mm × 2 mm) using a flow rate of 1.5 mL/min in a 2 min gradient elution with detection at 254 nm. The mobile phase was a mixture of methanol (solvent A) and water (solvent B), both containing formic acid at 0.1%. Gradient elution was as follows: 5:95 (A/B)–100:0 (A/B) over 1.25 min, 100:0 (A/B) for 0.5 min, and then reversion back to 5:95 (A/B) over 0.05 min, and finally 5:95 (A/B) for 0.2 min. Where “4 min ToF” is indicated, the method is as previous except at 30 °C, using a flow rate of 0.75 mL/min in a 4 min gradient elution as follows: 5:95 (A/B)–100:0 (A/B) over 2.5 min, 100:0 (A/B) for 1 min, and then reversion back to 5:95 (A/B) over 0.1 min, and finally 5:95 (A/B) for 0.4 min. Where “4 min ESI” is indicated, LC/MS and HRMS analyses were performed on a Waters Acquity UPLC and a diode array detector coupled to a Waters G2 QToF mass spectrometer fitted with a multimode ESI/APCI source. Analytical separation was carried out at 30 °C on a Phenomenex Kinetex C18 column (30 mm × 2.1 mm, 2.6 μ, 100 Å) using a flow rate of 0.3 mL/min in a 4 min gradient elution with detection at 254 nm. The mobile phase was a mixture of methanol (solvent A) and water (solvent B), both containing formic acid at 0.1%. Gradient elution was as follows: 10:90 (A/B)–90:10 (A/B) over 3 min, 90:10 (A/B) for 0.5 min, and then reversion back to 10:90 (A/B) over 0.3 min, and finally 10:90 (A/B) for 0.2 min.

### Preparation of Compounds

#### General Procedure A

Step 1: a suspension of 4-chloro-1-methyl-6-nitroquinolin-2(1*H*)-one **9** (1 equiv) or 4-chloro-6-nitroquinolin-2(1*H*)-one **5** (1 equiv) and the requisite amine (10 equiv) in NMP (0.3 M) was heated in the microwave at 160 °C for up to 16 h. The reaction mixture was allowed to cool to rt, and water was added. The resulting precipitate was filtered and washed with water to afford the desired 4-substituted-quinolinone, which was used without further purification. If no precipitate formed, the reaction mixture underwent HPLC purification to afford the desired product.

Step 2: a mixture of the requisite 4-substituted-6-nitroquinolin-2-one from step 1 (1 equiv) and SnCl_2_ (4 equiv) in a 6:1 mixture of ethanol: trifluoroethanol (0.01 M) was heated in the microwave at 120 °C for up to 2 h. The reaction mixture was loaded onto an SCX-2 column (2 g) and washed with methanol. The desired product was eluted with methanolic ammonia (approximately 2 M) and concentrated under reduced pressure to give the desired amino-quinolin-2-one, which was used with no further purification.

Step 3: the requisite 4-substituted, 6-amino-1-methyl-quinolin-2-one from step 2 (1 equiv), 2,4-dichloropyridine-3-carbonitrile (1.5 equiv), and triethylamine (2 equiv) in NMP (0.1 M) were heated in the MW at 120–160 °C for up to 1 h. The crude reaction mixture was diluted with dimethyl sulfoxide (DMSO) and MeCN and purified by Biotage reverse-phase chromatography [12 g C18 eluting 10–100% MeOH in water (containing 0.1% formic acid)] or via HPLC [40–100% MeOH in water] to give the desired product.

#### General Procedure B

Step 1: to a mixture of 4-chloro-1-methyl-6-nitroquinolin-2(1*H*)-one **9** (1 equiv), with requisite amine (1.5 equiv), cesium carbonate (3 equiv), palladium(II)acetate (20 mol %), and rac-BINAP (20 mol %) was added toluene (2 mL) and the vial was purged with argon for 5 min. The mixture was heated in the microwave at 120 °C for up to 4 h and then diluted with water and extracted with ethyl acetate. The organic layers were dried over MgSO_4_ and concentrated under reduced pressure. The resulting residue was purified by either normal-phase column chromatography (0–15% methanol in dichloromethane (DCM)) or reverse-phase column chromatography (10–100% methanol in water [0.1% formic acid modifier]) to give the desired 4-substituted nitroquinolin-2-one, which was subjected to further purification by SCX-2 if necessary.

Step 2: to a solution of 4-substituted, 6-nitro-1-methyl-quinolin-2-one from step 1 (1 equiv) in ethanol (0.05 M) or NMP (0.05 M) were added Pd/C (10%, 0.05 equiv) and ammonium formate (10 equiv) under argon. The sealed vessel was then placed into a preheated heating block at 60 °C and stirred for 30 min to 4 h. The reaction mixture was loaded onto an SCX-2 column (2 g) and washed with methanol. The desired product was eluted with methanolic ammonia (3.5 M) and concentrated under reduced pressure to give the desired amino-quinolin-2-one of sufficient purity for use in the subsequent step.

Step 3: a mixture of the product of step 2 (1 equiv), 2,4-dichloropyridine-3-carbonitrile (1.2 equiv) or 4,6-dichloro-5-cyano-pyridine-2-carboxylic acid (1.2 equiv), and DIPEA (1.4 equiv) in NMP (0.1 M) was heated to 120–160 °C for 1 h in the microwave. The crude reaction mixture was diluted with DMSO and MeCN and purified by reverse-phase chromatography (10–100% MeOH in water [0.1% formic acid modifier]) to give the desired product.

#### General Procedure C

Step 1: a mixture of the requisite amine (1.5 equiv), ethyl 4-chloro-1-methyl-6-nitro-2-oxo-quinoline-3-carboxylate **18** (1 equiv), and DIPEA (3 equiv) in NMP (0.2 M) was stirred either at 160 °C under microwave irradiation or at 80 °C in a heating block. Solid LiCl (6 equiv) was added to the mixture, and the reaction was further stirred under microwave irradiation at 120–160 °C for 1–6 h. The residue was taken up in water and extracted twice with EtOAc. The organic extracts were combined, washed with water and brine, then dried over MgSO_4_, and concentrated under reduced pressure. The crude residue was purified by reverse-phase chromatography [C18, 30–100% MeOH in water (containing 0.1% formic acid)] to give the desired 4-substituted nitroquinolin-2-one.

Step 2: to the requisite 4-substituted, 6-nitro-1-methyl-quinolin-2-one from step 1 (1 equiv) in ethanol (0.05 M) or NMP (0.05 M) were added Pd/C (10%, 0.05 equiv) and ammonium formate (10 equiv). The flask was sealed and evacuated and then refilled with argon three times. The flask was then placed into a preheated heating block at 60 °C and stirred for 30 min to 4 h. The reaction mixture was loaded onto an SCX-2 column (2 g) and washed with methanol. The desired product was eluted with methanolic ammonia (3.5 M) and concentrated under reduced pressure to give the desired amino-quinolin-2-one, which was used with no further purification.

Step 3: a mixture of the aniline product from step 2 (1 equiv), 2,4-dichloropyridine-3-carbonitrile (1.2 equiv), and DIPEA (1.4 equiv) in NMP (0.1 M) was heated under microwave irradiation to 120–160 °C for 1 h. The crude reaction mixture was diluted with DMSO (0.1 mL) and MeCN (0.1 mL) and directly loaded and purified by reverse-phase chromatography [12 g C18 column eluting 10–100% MeOH in water (containing 0.1% formic acid)] to give the desired product.

#### General Procedure D

Step 1: a mixture of 4-chloro-1-methyl-6-nitroquinolin-2(1*H*)-one **9** (1 equiv) and the requisite amine (10 equiv) in NMP (0.3 M) was heated in the microwave at 160 °C for up to 16 h. The reaction mixture was allowed to cool to rt, and water was added. The resulting precipitate was filtered and washed with water to afford the desired 4-substituted-quinolinone. If no precipitate formed, the reaction mixture underwent direct HPLC [Agilent 6120 MS-Prep LC using an ACE 5 C18-PFP 250 mm × 21.2 mm column using a 15 min gradient of 40–100% MeOH in water (containing 0.1% formic acid)].

Step 2: to a solution of the requisite 4-substituted, 6-nitro-1-methyl-quinolin-2-one from step 1 (1 equiv) in ethanol (0.05 M) or NMP (0.05 M) were added Pd/C (10%, 0.05 equiv) and ammonium formate (10 equiv). The flask was sealed and evacuated and then refilled with argon three times. The flask was then placed into a preheated heating block at 60 °C and stirred for 30 min to 4 h. The reaction mixture was loaded onto an SCX-2 column (2 g) and washed with methanol. The desired product was eluted with methanolic ammonia (3.5 M) and concentrated under reduced pressure to give the required amino-quinolin-2-one.

Step 3: a mixture of the 4-substituted, 6-amino-1-methyl-quinolin-2-one from step 2 (1 equiv) and 4,6-dichloro-5-cyano-pyridine-2-carboxylic acid (1.2 equiv) in NMP (0.1 M) was heated in a heating block at 100 °C for 2 h. The crude reaction mixture was diluted with DMSO (0.1 mL) and MeCN (0.1 mL) and directly loaded and purified by reverse-phase chromatography [12 g C18 column eluting 20–100% MeOH in water (containing 0.1% formic acid)] to give the desired product.

##### 2-Chloro-4-((1-methyl-2-oxo-1,2-dihydroquinolin-6-yl)amino)nicotinonitrile (**2**)

Step 1: a mixture of 6-nitroquinolin-2(1*H*)-one **3** (248 mg, 1.3 mmol), sodium hydride (60% in mineral oil, 63 mg, 1.6 mmol), and iodomethane (325 μL, 5.2 mmol) in DMF (8 mL) was stirred at 0 °C for 1 h. Brine was added, and the resulting precipitate was collected, washed with water, and dried to give 1-methyl-6-nitroquinolin-2(1*H*)-one (188 mg, 71%) as a white solid, which was used without further purification in the subsequent step. LCMS (2 min ToF) *R*_t_ = 1.08 min, *m*/*z* 205.1 [M + H]^+^.

Step 2: to a solution of 1-methyl-6-nitroquinolin-2(1*H*)-one (149 mg, 0.73 mmol) in ethanol:trifluoroethanol (3:1, 4 mL) was added SnCl_2_ (594 mg, 3.13 mmol) and the resulting mixture was heated in the microwave at 120 °C for 1 h. The reaction mixture was purified using an SCX-2 column and then further purified by flash column chromatography (5–10% methanol in DCM) to give 6-amino-1-methylquinolin-2(1*H*)-one (87 mg, 68%). ^1^H NMR (500 MHz, DMSO-*d*_6_) δ 7.66 (d, *J* = 9.4 Hz, 1H), 7.25 (d, *J* = 8.8 Hz, 1H), 6.94 (dd, *J* = 8.8, 2.6 Hz, 1H), 6.78 (d, *J* = 2.6 Hz, 1H), 6.48 (d, *J* = 9.4 Hz, 1H), 5.09 (s, 2H), 3.53 (s, 3H). LCMS (2 min ToF) *R*_t_ = 0.11 min, *m*/*z* 175 [M + H]^+^.

Step 3: a vial containing 6-amino-1-methylquinolin-2(1*H*)-one (20 mg, 0.115 mmol), 2,4-dichloropyridine-3-carbonitrile (20 mg, 0.115 mmol), and triethylamine (24 μL, 0.172 mmol) in DMF (1.5 mL) was heated in the microwave at 160 °C for 1 h. The crude reaction mixture was purified by HPLC to give the title compound (5 mg, 14%). ^1^H NMR (500 MHz, DMF-*d*_7_) δ 8.31 (d, *J* = 6.2 Hz, 1H), 8.14 (d, *J* = 9.5 Hz, 1H), 7.99 (d, *J* = 2.3 Hz, 1H), 7.91–7.83 (m, 2H), 7.14 (d, *J* = 6.2 Hz, 1H), 6.87 (d, *J* = 9.5 Hz, 1H), 3.91 (s, 3H); LCMS (4 min ToF) *R*_t_ = 2.48 min, *m*/*z* 311.0689 [M + H]^+^ expected 311.0694 for C_16_H_12_ClN_4_O.

##### 4-Chloro-6-nitroquinolin-2(1*H*)-one (**5**)

Step 1: to a stirred solution of 2,4-dichloroquinoline **4** (25 g, 127 mmol) in 1,4-dioxane (126 mL) was added concentrated HCl (12 M, 84 mL, 1018 mmol) dropwise. The reaction mixture was refluxed for 18 h. The mixture was cooled to room temperature, poured into excess ice water, and allowed to stir for 1 h. The obtained solid was filtered and dried under vacuum to afford 4-chloroquinolin-2(1*H*)-one (19.2 g, 85%) as an off-white solid. ^1^H NMR (500 MHz, DMSO-*d*_6_) δ 12.09–11.97 (m, 1H), 7.85 (dd, *J* = 8.1, 1.4 Hz, 1H), 7.62 (ddd, *J* = 8.4, 7.2, 1.4 Hz, 1H), 7.38 (dd, *J* = 8.3, 1.1 Hz, 1H), 7.30 (ddd, *J* = 8.2, 7.2, 1.1 Hz, 1H), 6.82 (s, 1H); LCMS (2 min ToF) *R*_t_ = 1.26 min, *m*/*z* 180.0 [M + H]^+^.

Step 2: to a stirred solution of 4-chloro-1*H*-quinolin-2-one from step 1 (24 g, 134 mmol) in sulfuric acid (71 mL, 1336 mmol) cooled to 0 °C was added nitric acid (70%, 13 mL, 147 mmol) dropwise. The solution was stirred at 0 °C for 1 h and then poured onto ice. The yellow precipitate that formed was filtered and washed with water until the pH of the filtrate was ∼7. The residue was then washed with methanol and diethyl ether. This crude residue was then dried under a high vacuum in a desiccator for 72 h to give the title compound (24.9 g, 79%) as a yellow powder. ^1^H NMR (500 MHz, DMSO-*d*_6_) δ 12.57 (s, 1H), 8.68–8.54 (m, 1H), 8.47–8.38 (m, 1H), 7.56–7.40 (m, 1H), 7.09–6.95 (m, 1H); LCMS (2 min ToF) *R*_t_ = 1.17 min, *m*/*z* 225.0056 [M + H]^+^ expected 225.0061 for C_9_H_6_ClN_2_O_3_^+^.

##### 2-Chloro-4-((4-(dimethylamino)-2-oxo-1,2-dihydroquinolin-6-yl)amino)nicotinonitrile (**8a**); 2-Chloro-4-((4-(ethylamino)-2-oxo-1,2-dihydroquinolin-6-yl)amino)nicotinonitrile (**8f**)

Prepared by **General Procedure A**, steps 1–2, from ethylamine (45 mg, 1.0 mmol). The resulting 6-amino-4-(ethylamino)quinolin-2(1*H*)-one (18 mg) was combined with 2,4-dichloropyridine-3-carbonitrile (15 mg) and triethylamine (25 μL) in DMF (1.5 mL) and heated in the MW at 160 °C for 1 h. The crude reaction mixture was diluted with water, and the resulting precipitate was purified by HPLC to give 2-chloro-4-((4-(ethylamino)-2-oxo-1,2-dihydroquinolin-6-yl)amino)nicotinonitrile **8f** (2 mg) as a yellow solid, and further elution gave 2-chloro-4-((4-(dimethylamino)-2-oxo-1,2-dihydroquinolin-6-yl)amino)nicotinonitrile **8a** (3 mg, 10%) as a yellow solid, the dimethylamino group coming from thermal decomposition of DMF solvent. **8a:**^1^H NMR (500 MHz, methanol-*d*_4_) δ 8.00 (d, *J* = 6.3 Hz, 1H), 7.80 (d, *J* = 2.2 Hz, 1H), 7.58–7.37 (m, 2H), 6.76 (d, *J* = 6.2 Hz, 1H), 5.97 (s, 1H), 3.00 (s, 6H); LCMS (4 min ESI) *R*_t_ = 2.32 min, *m*/*z* 340.0962 [M + H]^+^ expected 340.0965 for C_17_H_15_ClN_5_O.

Compound **8f** was reprepared using NMP as solvent to avoid **8a** byproduct. 6-Amino-4-(ethylamino)quinolin-2(1*H*)-one (8 mg, 0.04 mmol), 2,4-dichloropyridine-3-carbonitrile (7.5 mg, 0.043 mmol), and triethylamine (11 μL, 0.079 mmol) in NMP (2 mL) were stirred at 140 °C under microwave irradiation for 1 h, then purified by an SCX-2 column, further purified by HPLC, and desalted by an SCX-2 column. The title compound (4 mg, 30%) was obtained as a yellow solid. ^1^H NMR (500 MHz, DMF-*d*_7_) δ 10.82 (s, 1H), 9.52 (s, 1H), 8.12–8.06 (m, 2H), 7.54–7.45 (m, 2H), 6.92 (t, *J* = 5.0 Hz, 1H), 6.79 (d, *J* = 6.1 Hz, 1H), 5.40 (s, 1H), 3.27 (qd, *J* = 7.1, 4.5 Hz, 2H), 1.29 (t, *J* = 7.1 Hz, 3H). LCMS (4 min ESI) *R*_t_ = 2.43 min, *m*/*z* 340.0966 [M + H]^+^ expected 340.0965 for C_17_H_15_ClN_5_O.

##### 2-Chloro-4-((2-oxo-4-(piperidin-1-yl)-1,2-dihydroquinolin-6-yl)amino)nicotinonitrile (**8b**)

Prepared by **General Procedure A** from piperidine (48 μL, 0.49 mmol) to afford the title compound as a cream solid (5 mg, 36%). ^1^H NMR (500 MHz, DMSO- d_6_) δ 11.41 (s, 1H), 9.56 (s, 1H), 8.03 (d, *J* = 6.2 Hz, 1H), 7.48 (d, *J* = 2.4 Hz, 1H), 7.42 (dd, *J* = 8.7, 2.4 Hz, 1H), 7.32 (d, *J* = 8.7 Hz, 1H), 6.79 (d, *J* = 6.2 Hz, 1H), 5.86 (s, 1H), 3.01 (m, 4H), 1.70 (m, 4H), 1.60 (m, 2H); LCMS (4 min ToF) *R*_t_ = 2.86 min, *m*/*z* 380.1265 [M + H]^+^ expected 380.1273 for C_20_H_19_ClN_5_O.

##### 2-Chloro-4-((4-(4-methylpiperazin-1-yl)-2-oxo-1,2-dihydroquinolin-6-yl)amino)nicotinonitrile (**8c**)

Prepared by **General Procedure A** from 1-methylpiperazine (51 mg, 0.51 mmol) to afford the title compound as a yellow solid (7.5 mg, 61%). ^1^H NMR (500 MHz, methanol-*d*_4_) δ 8.02 (d, *J* = 6.2 Hz, 1H), 7.71 (d, *J* = 2.3 Hz, 1H), 7.54 (dd, *J* = 8.7, 2.3 Hz, 1H), 7.47 (d, *J* = 8.7 Hz, 1H), 6.81 (d, *J* = 6.2 Hz, 1H), 6.15 (s, 1H), 3.44–3.34 (m, broad, 4H), 3.24–3.11 (m, broad, 4H), 2.72 (s, 3H); LCMS (4 min ToF) *R*_t_ = 1.43 min, *m*/*z* 395.1383 [M + H]^+^ expected 395.1387 for C_20_H_20_ClN_6_O.

##### 2-Chloro-4-((4-morpholino-2-oxo-1,2-dihydroquinolin-6-yl)amino)nicotinonitrile (**8d**)

Prepared by **General Procedure A** from morpholine (48 μL, 0.51 mmol) to afford the title compound as a white solid (2.5 mg, 13%). ^1^H NMR (500 MHz, DMF-*d*_7_) δ 11.63 (s, 1H), 9.79 (s, 1H), 8.31 (d, *J* = 6.2 Hz, 1H), 7.98 (d, *J* = 2.3 Hz, 1H), 7.78 (dd, *J* = 8.7, 2.3 Hz, 1H), 7.73 (d, *J* = 8.7 Hz, 1H), 7.13 (d, *J* = 6.2 Hz, 1H), 6.17 (s, 1H), 4.04 (t, *J* = 4.5 Hz, 4H), 3.32 (t, *J* = 4.5 Hz, 4H); LCMS (4 min ToF) *R*_t_ = 2.51 min, *m*/*z* 382.1055 [M + H]^+^ expected 382.1065 for C_19_H_17_ClN_5_O_2_.

##### 2-Chloro-4-((4-(1-methyl-1*H*-pyrazol-4-yl)-2-oxo-1,2-dihydroquinolin-6-yl)amino)nicotinonitrile (**8e**)

Step 1: a suspension of 4-chloro-6-nitroquinolin-2(1*H*)-one **5** (23 mg, 0.102 mmol), 1-methyl-4-(4,4,5,5-tetramethyl-1,3,2-dioxaborolan-2-yl)-1*H*-pyrazole (25 mg, 0.120 mmol), tetrakis(triphenylphosphine)palladium(0) (14 mg, 0.012 mmol), DMF (3 mL), and 2 M aqueous sodium carbonate (0.18 mL, 0.36 mmol) was heated in the microwave at 140 °C for 1 h. The reaction mixture was partitioned between water and EtOAc, and the organic layer was washed with brine and water, dried over MgSO_4_, and concentrated under reduced pressure to give 14 mg of a yellow solid containing 4-(1-methyl-1*H*-pyrazol-4-yl)-6-nitroquinolin-2(1*H*)-one and residual triphenylphosphine oxide. This material was combined with SnCl_2_ (39 mg, 0.207 mmol) in a 6:1 mixture of ethanol: trifluoroethanol (0.01 M) and heated in the microwave at 120 °C for 1 h. The resulting mixture was loaded onto an SCX-2 column (2 g) and washed with methanol. The desired product was eluted with methanolic ammonia (2 M) and concentrated under reduced pressure to give 6-amino-4-(1-methyl-1*H*-pyrazol-4-yl)quinolin-2(1*H*)-one **7e** (6 mg, 48%) as a yellow solid, which was used without further purification in the subsequent step. LCMS (2 min ToF) *R*_t_ = 0.22 min, *m*/*z* 241.1108 expected 241.1084 for C_13_H_13_N_4_O^+^ [M + H]^+^.

Step 2: 6-amino-4-(1-methyl-1*H*-pyrazol-4-yl)quinolin-2(1*H*)-one from step 1 (6 mg, 0.025 mmol), 2,4-dichloropyridine-3-carbonitrile (6.5 mg, 0.037 mmol), and triethylamine (7 μL, 0.05 mmol) in DMF (1.5 mL) were heated in the microwave at 160 °C for 1 h. HPLC purification [40–100% MeOH in water (containing 0.1% formic acid)] afforded the title compound (1 mg, 11%) as a light brown solid. ^1^H NMR (500 MHz, MeOD-*d*_4_) 8.09 (s, 1H), 8.02 (d, *J* = 6.2 Hz, 1H), 7.88–7.85 (m, 2H), 7.59 (dd, *J* = 9.0, 2.2 Hz, 1H), 7.53 (d, *J* = 9.0 Hz, 1H), 6.81 (d, *J* = 6.2 Hz, 1H), 6.70 (s, 1H), 4.01 (s, 3H); LCMS (4 min ToF) *R*_t_ = 2.46 min, *m*/*z* 377.0888 [M + H]^+^ expected 377.0912 for C_19_H_14_ClN_6_O.

##### 4-Chloro-1-methyl-6-nitroquinolin-2(1*H*)-one (**9**)

To an ice-cooled suspension of 4-chloro-6-nitroquinolin-2(1*H*)-one **5** (10 g, 44.5 mmol) in DMF (60 mL) was added sodium hydride (60% in mineral oil, 3.56 g, 89.0 mmol), and the reaction was stirred for 10 min before iodomethane (5.54 mL, 89.0 mmol) was added portionwise. The reaction was stirred at room temperature for 90 min before water (80 mL) was added cautiously. After 10 min, the resulting precipitate was filtered, washed with water (80 mL) and diethyl ether (100 mL), and dried under vacuum to give the title compound (10 g, 85%) as a pale yellow solid. ^1^H NMR (500 MHz, DMSO-*d*_6_) δ 8.67 (d, *J* = 2.7 Hz, 1H), 8.51 (dd, *J* = 9.3, 2.7 Hz, 1H), 7.83 (d, *J* = 9.4 Hz, 1H), 7.19 (s, 1H), 3.67 (s, 3H); LCMS (2 min ToF) *R*_t_ = 1.32 min, *m*/*z* 239.0207 expected 239.0128 for C_10_H_8_ClN_2_O_3_^+^ [M + H]^+^.

##### 6-Chloro-5-cyano-4-((4-(ethylamino)-1-methyl-2-oxo-1,2-dihydroquinolin-6-yl)amino)picolinic Acid (**11a**)

Prepared by **General Procedure D** from ethylamine (2 M in THF, 31 mL, 63 mmol) affording the title compound (5.5 mg, 37%). ^1^H NMR (500 MHz, DMSO-*d*_6_) δ 13.68 (br s, 1 H), 9.92 (s, 1 H), 8.06–8.02 (m, 1 H), 7.54–7.52 (m, 2 H), 7.18 (s, 1 H), 6.80 (t, *J* = 5.0 Hz, 1 H), 5.45 (s, 1 H), 3.53 (s, 3 H), 3.20–3.14 (m, 2 H), 1.21 (t, *J* = 7.1 Hz, 3 H); LCMS (4 min ToF) *R*_t_ = 2.45 min, *m*/*z* 398.0992 [M + H]^+^ expected 398.1014 for C_19_H_17_ClN_5_O_3_.

##### 6-Chloro-5-cyano-4-((1-methyl-2-oxo-4-(propylamino)-1,2-dihydroquinolin-6-yl)amino)picolinic Acid (**11b**)

Prepared by **General Procedure D** from propylamine (207 μL, 2.51 mmol) affording the title compound as a pale orange solid (9 mg, 44%). ^1^H NMR (500 MHz, DMSO-*d*_6_) δ 9.90 (s, 1H), 8.06–8.05 (m, 1H), 7.55–7.50 (m, 2H), 7.19 (s, 1H), 6.85 (t, *J* = 5.3 Hz, 1H), 5.45 (s, 1H), 3.52 (s, 3H), 3.11 (q, *J* = 6.6 Hz, 2H), 1.62 (h, *J* = 7.3 Hz, 2H), 0.93 (t, *J* = 7.4 Hz, 3H); LCMS (4 min ESI) *R*_t_ = 2.39 min, *m*/*z* 412.1162 [M + H]^+^ expected 412.1176 for C_20_H_19_ClN_5_O_3_.

##### 6-Chloro-5-cyano-4-((4-((2-fluoroethyl)amino)-1-methyl-2-oxo-1,2-dihydroquinolin-6-yl)amino)picolinic Acid (**11c**)

Prepared by **General Procedure B** from 2-fluoroethylamine hydrochloride (63 mg, 0.629 mmol) to afford the title compound (5 mg, 23%) as a yellow solid. ^1^H NMR (500 MHz, DMSO-*d*_6_) δ 9.89 (s, 1H), 8.06 (s, 1H), 7.55 (d, *J* = 1.9 Hz, 2H), 7.17 (s, 1H), 7.06–7.02 (m, 1H), 5.57 (s, 1H), 4.67 (t, *J* = 4.9 Hz, 1H), 4.58 (t, *J* = 4.8 Hz, 1H), 3.57–3.50 (m, 4H), 3.48 (d, *J* = 5.0 Hz, 1H); LCMS (4 min ToF) *R*_t_ = 2.42 min, *m*/*z* 416.0919 [M + H]^+^ expected 416.0920 for C_19_H_16_ClFN_5_O_3_.

##### 6-Chloro-5-cyano-4-((4-((cyclopropylmethyl)amino)-1-methyl-2-oxo-1,2-dihydroquinolin-6-yl)amino)picolinic Acid (**11d**)

Prepared by **General Procedure B** from cyclopropylmethanamine (0.06 mL, 0.69 mmol) to afford the title compound (9 mg, 29%) as a yellow solid. ^1^H NMR (500 MHz, DMSO-*d*_6_) δ 9.93 (s, 1H), 8.08 (d, *J* = 2.0 Hz, 1H), 7.59–7.48 (m, 2H), 7.20 (s, 1H), 6.95 (t, *J* = 5.4 Hz, 1H), 5.48 (s, 1H), 3.53 (s, 3H), 3.10–2.91 (m, 2H), 1.13 (d, *J* = 5.5 Hz, 1H), 0.51–0.45 (m, 2H), 0.27–0.21 (m, 2H); LCMS (4 min ToF) *R*_t_ = 2.86 min, *m*/*z* 424.1165 [M + H]^+^ expected 424.1171 for C_21_H_19_ClN_5_O_3_.

##### 2-((6-((2-Chloro-3-cyanopyridin-4-yl)amino)-2-oxo-1,2-dihydroquinolin-4-yl)amino)-*N*-methylpropanamide (**12a**)

Prepared by **General Procedure A** from 2-amino-*N*-methylpropanamide (50 mg, 0.49 mmol) to afford the title compound (5 mg, 41%) as a light brown solid. ^1^H NMR (500 MHz, DMSO-*d*_6_) δ 10.96 (s, 1H), 8.10 (s, 1H), 8.10 (s, 1H), 8.03 (dd, *J* = 9.6, 5.7 Hz, 2H), 7.38 (dd, *J* = 8.7, 2.1 Hz, 1H), 7.28 (d, *J* = 8.7 Hz, 1H), 6.77 (d, *J* = 6.9 Hz, 1H), 6.60 (d, *J* = 6.2 Hz, 1H), 5.17 (s, 1H), 3.93 (q, *J* = 7.0 Hz, 1H), 2.61 (d, *J* = 4.6 Hz, 3H), 1.40 (d, *J* = 7.0 Hz, 3H); LCMS (4 min ToF) RT = 2.28 min, *m*/*z* 397.1166 [M + H]^+^ expected 397.1174 for C_19_H_18_ClN_6_O_2_.

##### (*R*)-2-((6-((2-Chloro-3-cyanopyridin-4-yl)amino)-2-oxo-1,2-dihydroquinolin-4-yl)amino)-*N*-methylpropanamide (**12b**)

Prepared by **General Procedure A** from (*R*)-2-amino-*N*-methylpropanamide (78 mg, 0.56 mmol) to afford the title compound (3 mg, 26%) as a light brown solid. ^1^H NMR (500 MHz, methanol-*d*_4_) δ 8.08 (d, *J* = 2.2 Hz, 1H), 8.00 (d, *J* = 6.2 Hz, 1H), 7.49 (dd, *J* = 8.7, 2.2 Hz, 1H), 7.43 (d, *J* = 8.7 Hz, 1H), 6.72 (d, *J* = 6.2 Hz, 1H), 5.45 (s, 1H), 4.09 (q, *J* = 7.0 Hz, 1H), 2.77 (s, 3H), 1.56 (d, *J* = 7.0 Hz, 3H); LCMS (4 min ToF) *R*_t_ = 2.30 min, *m*/*z* 397.1167 [M + H]^+^ expected 397.1174 for C_19_H_18_ClN_6_O_2_.

##### (*S*)-2-((6-((2-Chloro-3-cyanopyridin-4-yl)amino)-2-oxo-1,2-dihydroquinolin-4-yl)amino)-*N*-methylpropanamide (**12c**)

Prepared by **General Procedure A** from (*S*)-2-amino-*N*-methyl-propanamide hydrochloride (86 mg, 0.62 mmol) to afford the title compound (5.5 mg, 40%) as a light brown solid. ^1^H NMR (500 MHz, methanol-*d*_4_) δ 8.08 (d, *J* = 2.2 Hz, 1H), 7.99 (d, *J* = 6.2 Hz, 1H), 7.49 (dd, *J* = 8.7, 2.2 Hz, 1H), 7.43 (d, *J* = 8.7 Hz, 1H), 6.72 (d, *J* = 6.2 Hz, 1H), 5.45 (s, 1H), 4.09 (q, *J* = 7.0 Hz, 1H), 2.77 (s, 3H), 1.56 (d, *J* = 7.0 Hz, 3H); LCMS (4 min ToF) *R*_t_ = 2.29 min, *m*/*z* 397.1146 [M + H]^+^ expected 397.1174 for C_19_H_18_ClN_6_O_2_.

##### 2-((6-((2-Chloro-3-cyanopyridin-4-yl)amino)-2-oxo-1,2-dihydroquinolin-4-yl)amino)-2-cyclopropyl-*N*-methylacetamide (**12d**)

Prepared by **General Procedure D** from 2-amino-2-cyclopropyl-*N*-methylacetamide trifluoroacetic acid salt to afford the title compound as a white solid (2.7 mg, 9%). ^1^H NMR (600 MHz, methanol-*d*_4_) δ 8.14 (d, *J* = 2.2 Hz, 1H), 8.01 (d, *J* = 6.2 Hz, 1H), 7.50 (dd, *J* = 8.7, 2.2 Hz, 1H), 7.43 (d, *J* = 8.7 Hz, 1H), 6.73 (d, *J* = 6.2 Hz, 1H), 5.41 (s, 1H), 3.31 (d, *J* = 9.1 Hz, 1H), 2.80 (s, 3H), 1.40–1.32 (m, 1H), 0.76–0.66 (m, 2H), 0.66–0.59 (m, 1H), 0.46–0.39 (m, 1H); LCMS (4 min ESI) *R*_t_ = 2.27 min, *m*/*z* 423.1334 [M + H]^+^ expected 423.1336 for C_21_H_20_ClN_6_O_2_.

##### 2-((6-((2-Chloro-3-cyanopyridin-4-yl)amino)-2-oxo-1,2-dihydroquinolin-4-yl)amino)-*N*-methylacetamide; Formic Acid Salt (**12e**)

Prepared by **General Procedure A** from 2-amino-*N*-methylacetamide hydrochloride (58 mg, 0.46 mmol) to afford the title compound (6 mg, 64%) as a pale brown solid. ^1^H NMR (600 MHz, DMSO-*d*_6_/D_2_O) δ 8.30 (s, formic acid), 8.03 (d, *J* = 6.2 Hz, 1H), 7.89 (d, *J* = 2.3 Hz, 1H), 7.38 (dd, *J* = 8.6, 2.3 Hz, 1H), 7.29 (d, *J* = 8.6 Hz, 1H), 6.67 (d, *J* = 6.2 Hz, 1H), 3.75 (s, 2H), 2.61 (s, 3H); LCMS (4 min ESI) *R*_t_ = 2.17 min, *m*/*z* 383.1004 [M + H]^+^ expected 383.1018 for C_18_H_16_ClN_6_O_2_.

##### 2-((6-((2-Chloro-3-cyanopyridin-4-yl)amino)-1-methyl-2-oxo-1,2-dihydroquinolin-4-yl)amino)-*N*-methylpropanamide (**13a**)

Prepared by **General Procedure A** from 2-amino-*N*-methyl-propanamide (50 mg, 0.49 mmol) to afford the title compound (1.5 mg, 10%) as a cream solid. ^1^H NMR (500 MHz, methanol-*d*_4_) δ 8.14 (d, *J* = 2.3 Hz, 1H), 8.01 (d, *J* = 6.1 Hz, 1H), 7.67 (d, *J* = 9.0 Hz, 1H), 7.61 (dd, *J* = 9.0, 2.3 Hz, 1H), 6.75 (d, *J* = 6.1 Hz, 1H), 5.56 (s, 1H), 4.08 (q, *J* = 7.0 Hz, 1H), 3.69 (s, 3H), 2.77 (s, 3H), 1.57 (d, *J* = 7.0 Hz, 3H); ^13^C NMR (126 MHz, methanol-*d*_4_) δ 174.5, 164.1, 156.9, 153.5, 150.7, 150.4, 138.5, 131.9, 128.8, 119.8, 116.6, 116.1, 113.2, 106.8, 93.6, 92.1, 52.8, 28.3, 25.0, 17.4; LCMS (4 min ToF) *R*_t_ = 2.42 min, *m*/*z* 411.1329 [M + H]^+^ expected 411.1258 for C_20_H_20_ClN_6_O_2_.

##### 2-Chloro-4-((1-methyl-4-((1-(oxazol-2-yl)ethyl)amino)-2-oxo-1,2-dihydroquinolin-6-yl)amino)nicotinonitrile (**13b**)

Prepared by **General Procedure B** from 1-oxazol-2-ylethanamine (56 mg, 0.50 mmol) to afford the title compound (18 mg, 68%). ^1^H NMR **(**500 MHz, DMSO-*d*_6_) δ 8.18 (s, 1H), 8.05 (s, 1H), 8.04 (d, *J* = 6.2 Hz, 1H), 7.55–7.47 (m, 2H), 7.16 (s, 1H), 7.02 (br s, 1H), 7.65 (br s, 1H), 6.61 (d, *J* = 6.2 Hz, 1H), 5.56 (s, 1H), 5.00–4.87 (m, 1H), 3.50 (s, 3H), 1.62 (d, *J* = 6.9 Hz, 3H); LCMS (4 min ESI) *R*_t_ = 2.56 min, *m*/*z* 421.1169 [M + H]^+^ expected 421.1174 for C_21_H_18_ClN_6_O_2_.

##### 2-Chloro-4-((1-methyl-4-((1-(5-methyl-1,3,4-oxadiazol-2-yl)ethyl)amino)-2-oxo-1,2-dihydroquinolin-6-yl)amino)nicotinonitrile (**13c**)

Step 1: a mixture of 1-(5-methyl-1,3,4-oxadiazol-2-yl)ethanamine hydrochloride (26 mg, 0.157 mmol), ethyl 4-chloro-1-methyl-6-nitro-2-oxo-1,2-dihydroquinoline-3-carboxylate **18** (32.5 mg, 0.105 mmol), and *N,N*-diisopropylethylamine (0.05 mL, 0.3138 mmol) in NMP (1.5 mL) was stirred at 160 °C under microwave irradiation for 1 h. Solid LiCl (26 mg, 0.628 mmol) was then added to the mixture, and the reaction was further stirred under microwave irradiation at 160 °C for 1 h. The reaction mixture was purified by HPLC [40–100% MeOH in water (containing 0.1% formic acid)] to give 1-methyl-4-((1-(5-methyl-1,3,4-oxadiazol-2-yl)ethyl)amino)-6-nitroquinolin-2(1*H*)-one **19a** (14 mg, 41%); ^1^H NMR (500 MHz, DMSO) δ 9.18 (d, *J* = 2.5 Hz, 1H), 8.40 (dd, *J* = 9.3, 2.5 Hz, 1H), 7.80 (d, *J* = 7.5 Hz, 1H), 7.64 (d, *J* = 9.3 Hz, 1H), 5.75 (s, 1H), 5.17 (dq, *J* = 7.5, 6.9 Hz, 1H), 3.56 (s, 3H), 2.48 (s, 3H), 1.69 (d, *J* = 6.9 Hz, 3H), LCMS (2 min ToF), *R*_t_ = 1.19 min, *m*/*z* 330.1178 expected 330.1197 for C_15_H_16_N_5_O_4_^+^ [M + H]^+^.

Step 2: a mixture of 1-methyl-4-((1-(5-methyl-1,3,4-oxadiazol-2-yl)ethyl)amino)-6-nitroquinolin-2(1*H*)-one from step 1 (12.5 mg, 0.038 mmol) and SnCl_2_ (28 mg, 0.151 mmol) in a 6:1 mixture of ethanol: trifluoroethanol (0.01 M) was heated in the microwave at 120 °C for 1 h. The reaction mixture was loaded onto an SCX-2 column (2 g) and washed with methanol. The desired product was eluted with methanolic ammonia (2 M) and concentrated under reduced pressure to give 6-amino-1-methyl-4-((1-(5-methyl-1,3,4-oxadiazol-2-yl)ethyl)amino)quinolin-2(1*H*)-one **20a** (11 mg, 97%), which was used without further purification in the subsequent step; ^1^H NMR (500 MHz, methanol-*d*_4_) δ 7.34 (d, *J* = 9.0 Hz, 1H), 7.28 (d, *J* = 2.5 Hz, 1H), 7.18–7.07 (m, 1H), 5.70 (s, 1H), 5.09 (q, *J* = 6.9 Hz, 1H), 3.59 (s, 3H), 2.52 (s, 3H), 1.79 (d, *J* = 6.9 Hz, 3H), LCMS (2 min ToF), *R*_t_ = 0.47 min, *m*/*z* 300.1443 expected 300.1455 for C_15_H_18_N_5_O_2_ [M + H]^+^.

Step 3: a mixture of 6-amino-1-methyl-4-((1-(5-methyl-1,3,4-oxadiazol-2-yl)ethyl)amino)quinolin-2(1*H*)-one from step 2 (11 mg, 0.037 mmol), 2,4-dichloropyridine-3-carbonitrile (8.9 mg, 0.11 mmol), and DIPEA (19 μL, 0.11 mmol) in NMP (1.5 mL) was heated in the MW at 160 °C for 1 h. Purification by HPLC [40–100% MeOH in water (containing 0.1% formic acid)] afforded the title compound (1 mg, 6%). ^1^H NMR (500 MHz, methanol-*d*_4_) δ 8.11 (d, *J* = 2.2 Hz, 1H), 8.00 (d, *J* = 6.2 Hz, 1H), 7.67 (d, *J* = 9.0 Hz, 1H), 7.60 (dd, *J* = 9.0, 2.2 Hz, 1H), 6.72 (d, *J* = 6.2 Hz, 1H), 5.78 (s, 1H), 5.12 (q, *J* = 6.9 Hz, 1H), 3.68 (s, 3H), 2.52 (s, 3H), 1.78 (d, *J* = 6.9 Hz, 3H); LCMS (4 min ToF) *R*_t_ = 2.48 min, *m*/*z* 436.1271 [M + H]^+^ expected 436.1283 for C_21_H_19_ClN_7_O_2_.

##### 2-Chloro-4-((1-methyl-4-((1-(1-methyl-1*H*-1,2,4-triazol-3-yl)ethyl)amino)-2-oxo-1,2-dihydroquinolin-6-yl)amino)nicotinonitrile (**13d**)

1-(1-Methyl-1*H*-1,2,4-triazol-3-yl)ethan-1-amine hydrochloride (84 mg, 0.51 mmol) was used according to **General Procedure C** to afford the title compound (6 mg, 46%) as a cream solid. ^1^H NMR (500 MHz, CDCl_3_) δ 8.04 (d, *J* = 6.1 Hz, 1H), 7.95 (s, 1H), 7.56 (d, *J* = 2.4 Hz, 1H), 7.40 (dd, *J* = 9.0, 2.4 Hz, 1H), 7.35 (d, *J* = 9.0 Hz, 1H), 7.12 (s, 1H), 6.58 (d, *J* = 6.1 Hz, 1H), 5.81 (s, 1H), 5.78 (d, *J* = 6.8 Hz, 1H), 4.85 (app. quin., *J* = 6.7 Hz, 1H), 3.89 (s, 3H), 3.61 (s, 3H), 1.66 (d, *J* = 6.7 Hz, 3H); LCMS (4 min ToF) *R*_t_ = 2.47 min, *m*/*z* 435.1440 [M + H]^+^ expected 435.1443 for C_21_H_20_ClN_8_O.

##### 2-Chloro-4-((1-methyl-2-oxo-4-((1-(pyrimidin-2-yl)ethyl)amino)-1,2-dihydroquinolin-6-yl)amino)nicotinonitrile (**13e**)

Prepared by **General Procedure B** from 1-pyrimidin-2-ylethanamine hydrochloride (232 mg, 1.89 mmol) to afford the title compound (18.4 mg, 49%) as a yellow solid. ^1^H NMR (500 MHz, DMSO-*d*_6_) δ 9.62 (s, 1H), 8.79 (d, *J* = 4.9 Hz, 2H), 8.29 (d, *J* = 2.2 Hz, 1H), 8.05 (d, *J* = 6.2 Hz, 1H), 7.56–7.44 (m, 2H), 7.40 (t, *J* = 4.9 Hz, 1H), 7.23 (d, *J* = 7.0 Hz, 1H), 6.61 (d, *J* = 6.2 Hz, 1H), 5.27 (s, 1H), 4.74 (app quin, *J* = 6.9 Hz, 1H), 3.46 (s, 3H), 1.60 (d, *J* = 6.9 Hz, 3H). LCMS (4 min ToF) *R*_t_ = 2.55 min, *m*/*z* 432.1329 [M + H]^+^ expected 432.1334 for C_22_H_19_ClN_7_O.

##### (*R*)-2-Chloro-4-((1-methyl-2-oxo-4-((1-(pyrimidin-2-yl)ethyl)amino)-1,2-dihydroquinolin-6-yl)amino)nicotinonitrile (**13f**)

Prepared by **General Procedure C** from (*R*)-1-(pyrimidin-2-yl)ethan-1-amine (30 mg, 0.24 mmol) to yield the title compound (7 mg, 48%). ^1^H NMR (500 MHz, DMSO-*d*_6_) δ 9.62 (s, 1H), 8.79 (d, *J* = 4.9 Hz, 2H), 8.29 (d, *J* = 2.2 Hz, 1H), 8.05 (d, *J* = 6.2 Hz, 1H), 7.56–7.44 (m, 2H), 7.40 (t, *J* = 4.9 Hz, 1H), 7.23 (d, *J* = 7.0 Hz, 1H), 6.61 (d, *J* = 6.2 Hz, 1H), 5.27 (s, 1H), 4.74 (p, *J* = 6.9 Hz, 1H), 3.46 (s, 3H), 1.60 (d, *J* = 6.9 Hz, 3H); LCMS (4 min ToF) *R*_t_ = 2.53 min, *m*/*z* 432.1309 [M + H]^+^ expected 432.1334 for C_22_H_19_ClN_7_O.

##### 2-Chloro-4-((1-methyl-2-oxo-4-((2-(pyrimidin-2-yl)propan-2-yl)amino)-1,2-dihydroquinolin-6-yl)amino)nicotinonitrile (**13g**)

Prepared by **General Procedure B** from 2-pyrimidin-2-ylpropan-2-amine dihydrochloride (79 mg, 0.378 mmol) to afford the title compound (16 mg, 58%) as a pale orange solid. ^1^H NMR (500 MHz, DMSO-*d*_6_) δ 9.59 (s, 1H), 8.82 (d, *J* = 4.9 Hz, 2H), 8.25 (d, *J* = 2.3 Hz, 1H), 8.05 (d, *J* = 6.2 Hz, 1H), 7.51 (dd, *J* = 9.0, 2.3 Hz, 1H), 7.46 (d, *J* = 9.0 Hz, 1H), 7.39 (t, *J* = 4.9 Hz, 1H), 6.85 (s, 1H), 6.61 (d, *J* = 6.2 Hz, 1H), 4.69 (s, 1H), 3.41 (s, 3H), 1.76 (s, 6H); ^13^C NMR (126 MHz, DMSO-*d*_6_) δ 172.5, 161.2, 157.5, 156.7, 153.1, 151.4, 147.4, 138.4, 131.0, 128.6, 120.6, 119.4, 116.1, 116.0, 114.3, 107.5, 94.4, 92.3, 58.8, 28.4, 27.5.; LCMS (4 min ToF) *R*_t_ = 2.64 min, *m*/*z* 446.1482 [M + H]^+^ expected 446.1491 for C_23_H_21_ClN_7_O.

##### 4-Chloro-1-(cyclopropylmethyl)-6-nitroquinolin-2(1*H*)-one (**14a**)

To a suspension of 4-chloro-6-nitroquinolin-2(1*H*)-one **5** (100 mg, 0.45 mmol) in DMF (2 mL) were added bromomethylcyclopropane (86 μL, 0.89 mmol) and cesium carbonate (219 mg, 0.67 mmol). The reaction was stirred at rt for 42 h. The reaction was diluted with DCM and washed with 1 M aq HCl and brine. The organic layer was dried and concentrated under reduced pressure and then purified by column chromatography (12–100% EtOAc in cyclohexane) affording the title compound (62 mg, 50%) as a yellow solid. ^1^H NMR (500 MHz, CDCl_3_) δ 8.90 (d, *J* = 2.2 Hz, 1H), 8.46 (dd, *J* = 9.3, 2.6 Hz, 1H), 7.65 (d, *J* = 9.3 Hz, 1H), 7.00 (s, 1H), 4.27 (d, *J* = 7.0 Hz, 2H), 1.28–1.17 (m, 1H), 0.57 (d, *J* = 6.5 Hz, 4H). LCMS (2 min ToF) *R*_t_ = 1.51 min, *m*/*z* 279.0530 expected 279.0531 for C_13_H_12_ClN_2_O_3_^+^ [M + H]^+^.

##### 4-Chloro-1-(cyclobutylmethyl)-6-nitroquinolin-2(1*H*)-one (**14b**)

Prepared from 4-chloro-6-nitroquinolin-2(1*H*)-one **5** (250 mg, 1.11 mmol) and (bromomethyl)cyclobutane (250 μL, 2.23 mmol) using method as for **14a** affording the title compound (116 mg, 34%) as a yellow solid. ^1^H NMR (500 MHz, CDCl_3_) δ 8.93 (d, *J* = 2.6 Hz, 1H), 8.45 (dd, *J* = 9.4, 2.6 Hz, 1H), 7.49 (d, *J* = 9.4 Hz, 1H), 7.02 (s, 1H), 4.43 (d, *J* = 7.1 Hz, 2H), 2.86–2.70 (m, 1H), 2.11–2.01 (m, 2H), 2.02–1.84 (m, 4H); LCMS (2 min ToF) *R*_t_ = 1.56 min, *m*/*z* 293.0676 expected 293.0687 for C_14_H_14_ClN_2_O_3_ [M + H]^+^.

##### 2-Chloro-4-((1-(cyclopropylmethyl)-2-oxo-4-((1-(pyrimidin-2-yl)ethyl)amino)-1,2-dihydroquinolin-6-yl)amino)nicotinonitrile (**17a**)

**14a** (62 mg, 0.22 mmol) and 1-pyrimidin-2-ylethanamine hydrochloride (53 mg, 0.33 mmol) were used in an analogous fashion to **General Procedure B** to afford the title compound (1.3 mg, 11%) as a white solid. ^1^H NMR (600 MHz, methanol-*d*_4_) δ 8.80 (d, *J* = 4.9 Hz, 2H), 8.19 (d, *J* = 2.3 Hz, 1H), 8.03 (d, *J* = 6.2 Hz, 1H), 7.78 (d, *J* = 9.0 Hz, 1H), 7.61 (dd, *J* = 9.0, 2.3 Hz, 1H), 7.40 (t, *J* = 4.9 Hz, 1H), 6.79 (d, *J* = 6.3 Hz, 1H), 5.53 (s, 1H), 4.93–4.89 (m, 1H), 4.21 (d, *J* = 6.8 Hz, 2H), 1.71 (d, *J* = 6.9 Hz, 3H), 1.36–1.20 (m, 1H), 0.52–0.43 (m, 4H). LCMS (4 min ESI) *R*_t_ = 2.71 min, *m*/*z* 472.1650 [M + H]^+^ expected 472.1653 for C_25_H_23_ClN_7_O.

##### 2-Chloro-4-((1-(cyclobutylmethyl)-2-oxo-4-((1-(pyrimidin-2-yl)ethyl)amino)-1,2-dihydroquinolin-6-yl)amino)nicotinonitrile (**17b**)

**14b** (50 mg, 0.17 mmol) and 1-pyrimidin-2-ylethanamine hydrochloride (55 mg, 0.34 mmol) were used in an analogous fashion to **General Procedure B** to afford the title compound (4.5 mg, 27%) as a brown solid. ^1^H NMR (600 MHz, methanol-*d*_4_) δ 8.80 (d, *J* = 4.9 Hz, 2H), 8.17 (d, *J* = 2.3 Hz, 1H), 8.03 (d, *J* = 6.2 Hz, 1H), 7.63 (d, *J* = 9.1 Hz, 1H), 7.58 (dd, *J* = 9.0, 2.4 Hz, 1H), 7.40 (t, *J* = 4.9 Hz, 1H), 6.78 (d, *J* = 6.2 Hz, 1H), 5.53 (s, 1H), 4.92–4.88 (m, 1H), 4.36 (d, *J* = 7.1 Hz, 2H), 2.85–2.75 (m, 1H), 2.06–1.81 (m, 6H), 1.71 (d, *J* = 6.9 Hz, 3H); LCMS (4 min ESI) *R*_t_ = 2.89 min, *m*/*z* 486.1804 [M + H]^+^ expected 486.1809 for C_26_H_25_ClN_7_O.

##### Ethyl 4-Chloro-1-methyl-6-nitro-2-oxo-1,2-dihydroquinoline-3-carboxylate (**18**)

Step 1: to a solution of 5-nitro-isatoic anhydride (25.1 g, 121 mmol) in DMF (241 mL) was added sodium hydride (60% in mineral oil, 7.24 g, 181 mmol). The solution was allowed to stir at room temperature for 15 min. Iodomethane (19 mL, 301 mmol) was added, and the mixture was stirred for 4 h at room temperature. The reaction mixture was poured onto ice and then filtered and washed with water (5 L). The solid was collected and dried under vacuum overnight affording 1-methyl-6-nitro-2*H*-benzo[*d*][1,3]oxazine-2,4(1*H*)-dione (19.9 g, 73%) as an orange powder. ^1^H NMR (500 MHz, DMSO-*d*_6_) δ 8.64 (d, *J* = 2.5 Hz, 1H), 8.61 (dd, *J* = 9.1, 2.7 Hz, 1H), 7.66 (d, *J* = 9.1 Hz, 1H), 3.54 (s, 3H).

Step 2: to a solution of 1-methyl-6-nitro-2*H*-benzo[*d*][1,3]oxazine-2,4(1*H*)-dione (19.6 g, 88.2 mmol) in DMF (176 mL) was added diethyl malonate (40 mL, 265 mmol). The solution was cooled to 0 °C, and sodium hydride (60% in mineral oil, 7.06 g, 176 mmol) was added in 4 portions over 30 min. The solution was allowed to warm to room temperature and stirred for 4 h. Water was added with care to the reaction mixture followed by 10% aq. HCl until the pH of the mixture was ∼5. The resulting precipitate was filtered and washed with water (5 L) and dried under vacuum to give ethyl 4-hydroxy-1-methyl-6-nitro-2-oxo-1,2-dihydroquinoline-3-carboxylate (24.2 g, 94%) as a pale yellow solid, which was used without further purification in the subsequent step. LCMS (2 min ToF) *R*_t_ = 1.45 min, *m*/*z* 293.0739 expected 293.0768 for C_13_H_13_N_2_O_6_^+^ [M + H]^+^.

Step 3: a mixture of ethyl 4-hydroxy-1-methyl-6-nitro-2-oxo-1,2-dihydroquinoline-3-carboxylate (24.1 g, 82.5 mmol) and phosphorus oxychloride (250 mL, 2674 mmol) was heated to 80°C under argon with stirring for 2.5 h. The reaction was concentrated under reduced pressure. *CAUTION, apply appropriate procedures in the disposal of POCl*_*3*_. The residue was diluted with water and extracted with EtOAc. The combined organic extracts were washed with brine and then dried over MgSO_4_. The crude residue was purified (by dry loading onto silica) using column chromatography (340 g, KP-Sil, 0–10% MeOH in DCM) to afford the title compound (14.5 g, 57%) as a dark orange solid. ^1^H NMR (500 MHz, CDCl_3_) δ 8.95 (d, *J* = 2.5 Hz, 1H), 8.50 (dd, *J* = 9.3, 2.5 Hz, 1H), 7.53 (d, *J* = 9.3 Hz, 1H), 4.48 (q, *J* = 7.1 Hz, 2H), 3.78 (s, 3H), 1.42 (t, *J* = 7.1 Hz, 3H); LCMS (2 min ToF), *R*_t_ = 1.42 min, *m*/*z* 311.0427 expected 311.0429 for C_13_H_12_ClN_2_O_5_ [M + H]^+^.

##### 4-((1-(5-Bromopyrimidin-2-yl)ethyl)amino)-1-methyl-6-nitroquinolin-2(1*H*)-one (**21a**)

A mixture of ethyl 4-chloro-1-methyl-6-nitro-2-oxo-quinoline-3-carboxylate **18** (1.2 g, 3.9 mmol), 1-(5-bromopyrimidin-2-yl)ethanamine (940 mg, 4.6 mmol), and DIPEA (2 mL, 11.6 mmol) in NMP (9.7 mL) was stirred at 80 °C overnight. Once cooled to rt, lithium chloride (980 mg, 23 mmol) was added to the mixture, which was then heated to 160 °C for 2 h. The residue was taken up in water and extracted twice with EtOAc. The organic extracts were washed twice with 1 M aq. NaOH and brine and then dried over MgSO_4_. The residue was purified by flash column chromatography eluting 50–100% EtOAc in cyclohexane. The title compound was isolated as a mustard solid (895 mg, 52%) in 90% purity and used without further purification in the subsequent step. LCMS (method T2) *R*_t_ = 1.38 min, *m*/*z* 404.0291 expected 404.0353 for C_16_H_15_BrN_5_O_3_ [M + H]^+^.

##### 4-((2-(5-Bromopyrimidin-2-yl)propan-2-yl)amino)-1-methyl-6-nitroquinolin-2(1*H*)-one (**21b**)

A suspension of ethyl 4-chloro-1-methyl-6-nitro-2-oxo-quinoline-3-carboxylate **18** (0.5 g, 1.61 mmol), 1-(5-bromopyrimidin-2-yl)-1-methylethylamine hydrochloride (513 mg, 1.93 mmol), and DIPEA (1.1 mL, 6.44 mmol) was stirred at 120 °C for 6 h. Once cooled to rt, lithium chloride (409 mg, 9.66 mmol) was added to the mixture and heated to 160 °C overnight. Once cooled to rt, the residue was taken up in water and extracted twice with EtOAc. The combined organic extracts were washed with water and brine and then dried over MgSO_4_. The residue was purified by flash column chromatography (Biotage KP-Sil 25 g eluting 0–10% MeOH in DCM). A second purification was required, so the residue was purified again (Biotage KP-Sil 25 g eluting 20–100% EtOAc in cyclohexane) affording the title compound (405 mg, 60%) as a yellow solid. ^1^H NMR (500 MHz, DMSO-*d*_6_) δ 9.27 (d, *J* = 2.6 Hz, 1H), 9.03 (s, 2H), 8.37 (dd, *J* = 9.3, 2.6 Hz, 1H), 7.57 (d, *J* = 9.3 Hz, 1H), 7.54 (s, 1H), 4.71 (s, 1H), 3.46 (s, 3H), 1.79 (s, 6H). LCMS (method T2) *R*_t_ = 1.42 min, *m*/*z* 418.0502 expected 418.0509 for C_17_H_17_BrN_5_O_3_ [M + H]^+^.

##### 2-Chloro-4-((1-methyl-4-((1-(5-methylpyrimidin-2-yl)ethyl)amino)-2-oxo-1,2-dihydroquinolin-6-yl)amino)nicotinonitrile (**24a**)

Step 1: to 4-((1-(5-bromopyrimidin-2-yl)ethyl)amino)-1-methyl-6-nitroquinolin-2(1*H*)-one **21a** (22 mg, 0.054 mmol), bis(triphenylphosphine)palladium(II) chloride (19 mg, 0.027 mmol), and methylboronic acid (32.6 mg, 0.54 mmol) were added 1,4-dioxane (0.54 mL) and 2 M aq sodium carbonate (0.14 mL, 0.28 mmol). The mixture was then heated under an argon atmosphere in the microwave at 120 °C for 2 h. Once cooled to rt, the reaction mixture was filtered through a syringe filter, and the resulting filtrate was concentrated under reduced pressure. DMSO (0.5 mL) was added to the sample, which was purified using reverse-phase C18 column eluting from 10 to 100% MeOH in water (containing 0.1% formic acid). 1-Methyl-4-((1-(5-methylpyrimidin-2-yl)ethyl)amino)-6-nitroquinolin-2(1*H*)-one **22a** (5 mg, 27%) was obtained as an off-white solid. ^1^H NMR (500 MHz, chloroform-*d*) δ 8.68 (d, *J* = 2.5 Hz, 1H), 8.61 (s, 2H), 8.39 (dd, *J* = 9.3, 2.5 Hz, 1H), 7.40 (d, *J* = 9.3 Hz, 1H), 6.53 (d, *J* = 6.6 Hz, 1H), 5.82 (s, 1H), 4.87 (app. quin., *J* = 6.7 Hz, 1H), 3.67 (s, 3H), 2.35 (s, 3H), 1.67 (d, *J* = 6.7 Hz, 3H). LCMS (method T2) *R*_t_ = 1.30 min, *m*/*z* 340.1386 expected 340.1404 for C_17_H_18_N_5_O_3_ [M + H]^+^.

Step 2: to a solution of 1-methyl-4-((1-(5-methylpyrimidin-2-yl)ethyl)amino)-6-nitroquinolin-2(1*H*)-one from step 1 (5 mg, 0.015 mmol) in ethanol (1 mL) were added ammonium formate (9.3 mg, 0.15 mmol) and Pd/C (10 wt %) (10 mg). The resulting mixture was stirred in a sealed vial under argon at 60 °C for 1 h. 6-Amino-1-methyl-4-((1-(5-methylpyrimidin-2-yl)ethyl)amino)quinolin-2(1*H*)-one **23a** (4 mg, 88%) was obtained as a pale orange solid following SCX purification and used without further purification in the subsequent step. LCMS (2 min ToF) *R*_t_ = 0.87 min, *m*/*z* 310.1658 expected 310.1662 for C_17_H_20_N_5_O [M + H]^+^.

Step 3: a mixture of the product from step 2 (4 mg, 0.0129 mmol), 2,4-dichloropyridine-3-carbonitrile (2.7 mg, 0.016 mmol), NMP (3.96 mL), and triethylamine (3.6 μL, 0.0259 mmol) under argon was heated in the microwave for 1 h at 160 °C. DMSO (0.5 mL) was added, and the sample was purified by reverse-phase chromatography (C18, 30–100% MeOH in water (containing 0.1% formic acid)) to yield the title compound (2 mg, 35%) as an off-white solid. ^1^H NMR (600 MHz, CDCl_3_) δ 8.61 (s, 2H), 8.10 (d, *J* = 6.1 Hz, 1H), 7.60 (d, *J* = 2.2 Hz, 1H), 7.48 (dd, *J* = 8.9, 2.2 Hz, 1H), 7.44 (d, *J* = 8.9 Hz, 1H), 7.00 (s, 1H), 6.67 (d, *J* = 6.1 Hz, 1H), 6.41 (d, *J* = 6.4 Hz, 1H), 5.85 (s, 1H), 4.88 (app. quin., *J* = 6.6 Hz, 1H), 3.69 (s, 3H), 2.36 (s, 3H) 1.67 (d, *J* = 6.7 Hz, 3H); ^13^C NMR (151 MHz, CDCl_3_) δ 167.1, 163.2, 157.4, 156.1, 153.9, 151.7, 147.7, 139.5, 129.8, 129.1, 127.8, 118.6, 116.6, 116.5, 113.9, 105.7, 94.4, 94.4, 53.3, 29.1, 20.9, 15.4. LCMS (4 min ToF) *R*_t_ = 2.61 min, *m*/*z* 446.1480 [M + H]^+^ expected 446.1491 for C_23_H_21_ClN_7_O.

##### 2-Chloro-4-((4-((1-(5-cyclopropylpyrimidin-2-yl)ethyl)amino)-1-methyl-2-oxo-1,2-dihydroquinolin-6-yl)amino)nicotinonitrile (**24b**)

Method as for **24a** starting from cyclopropylboronic acid (26 mg, 0.309 mmol) to afford the title compound (8 mg, 57%) as an off-white solid. ^1^H NMR (600 MHz, CDCl_3_) δ 8.47 (s, 2H), 8.06 (d, *J* = 6.1 Hz, 1H), 7.58 (d, *J* = 2.4 Hz, 1H), 7.44 (dd, *J* = 8.9, 2.4 Hz, 1H), 7.40 (d, *J* = 8.9 Hz, 1H), 7.09 (s, 1H) 6.63 (d, *J* = 6.1 Hz, 1H), 6.43 (d, *J* = 6.4 Hz, 1H), 5.80 (s, 1H), 4.83 (app. quin., *J* = 6.6 Hz, 1H), 3.65 (s, 3H), 1.90–1.83 (m, 1H), 1.62 (d, *J* = 6.7 Hz, 3H), 1.15–1.06 (m, 2H), 0.83–0.74 (m, 2H); ^13^C NMR (151 MHz, CDCl_3_) δ 166.8, 163.2, 156.1, 155.1, 151.7, 147.7, 139.4, 135.3, 129.8, 127.8, 118.6, 116.6, 116.5, 113.9, 105.7, 94.3, 94.3, 53.3, 29.1, 20.8, 10.7, 8.9, 8.9. LCMS (4 min ToF) *R*_t_ = 2.73 min, *m*/*z* 472.1633 [M + H]^+^ expected 472.1633 for C_25_H_23_ClN_7_O.

##### 2-Chloro-4-((4-((2-(5-cyclopropylpyrimidin-2-yl)propan-2-yl)amino)-1-methyl-2-oxo-1,2-dihydroquinolin-6-yl)amino)nicotinonitrile (**25**) (CCT369347)

Step 1: a mixture of cyclopropylboronic acid (104 mg, 1.2 mmol), tetrakis(triphenylphosphine)palladium(0) (17.4 mg, 0.015 mmol), 4-((2-(5-bromopyrimidin-2-yl)propan-2-yl)amino)-1-methyl-6-nitroquinolin-2(1*H*)-one **21b** (63 mg, 0.15 mmol), and sodium carbonate (2 M aq, 0.19 mL, 0.38 mmol) in DMF (2.4 mL) was stirred under microwave irradiation at 140°C for 1 h. The solid was removed by filtration, washing with DMF. Brine was then added to the filtrate, and the precipitate formed was collected, washed with water, and dried to afford 4-((2-(5-cyclopropylpyrimidin-2-yl)propan-2-yl)amino)-1-methyl-6-nitroquinolin-2(1*H*)-one **22c** (24 mg) as a light brown solid, which was used without further purification in the subsequent step. LCMS (2 min ESI) *R*_t_ = 1.52 min, *m*/*z* 380.1714, expected 380.1717 for C_20_H_22_N_5_O_3_ [M + H]^+^.

Step 2: to a solution of the product from step 1 (24 mg) in ethanol (1 mL) were added ammonium formate (40 mg, 0.63 mmol) and Pd/C (10%, 6.7 mg, 0.0063 mmol). The suspension was stirred under microwave irradiation at 60 °C for 1 h, filtered, and the filtrate was purified using an SCX-2 column to afford 6-amino-4-((2-(5-cyclopropylpyrimidin-2-yl)propan-2-yl)amino)-1-methylquinolin-2(1*H*)-one **23c** (17 mg, 32% over two steps) as a yellow oil. LCMS (4 min ToF) *R*_t_ 1.68 min, *m*/*z* 350.1988 expected 350.1975 for C_20_H_24_N_5_O [M + H]^+^.

Step 3: a mixture of 6-amino-4-[[1-(5-cyclopropylpyrimidin-2-yl)-1-methyl-ethyl]amino]-1-methyl-quinolin-2-one from step 2 (7 mg, 0.02 mmol), 2,4-dichloropyridine-3-carbonitrile (5.2 mg, 0.03 mmol), and triethylamine (5.6 μL, 0.04 mmol) in NMP (0.6 mL) under argon was heated in the microwave for 1 h at 160 °C. The mixture was diluted with DMSO (0.5 mL) and purified by reverse-phase chromatography (C18, 30–100% MeOH in water (containing 0.1% formic acid)). The isolated material was then passed through a silica plug, eluting with 5% MeOH in DCM to give the title compound (3 mg, 31%) as an off-white solid. ^1^H NMR (600 MHz, DMSO-*d*_6_) δ 9.58 (s, 1H, NH), 8.56 (s, 2H), 8.24 (d, *J* = 2.3 Hz, 1H), 8.05 (d, *J* = 6.2 Hz, 1H), 7.51 (dd, *J* = 9.0, 2.3 Hz, 1H), 7.45 (d, *J* = 9.0 Hz, 1H), 6.81 (s, 1H, NH), 6.61 (d, *J* = 6.2 Hz, 1H), 4.71 (s, 1H), 3.42 (s, 3H), 1.96–1.89 (m, 1H), 1.73 (s, 6H), 1.06–0.98 (m, 2H), 0.87–0.81 (m, 2H); ^13^C NMR (151 MHz, DMSO-*d*_6_) δ 169.6, 161.2, 156.7, 154.6, 153.1, 151.4, 147.5, 138.4, 134.4, 131.0, 128.6, 120.6, 116.1, 116.0, 114.3, 107.5, 94.4, 92.3, 58.5, 28.4, 27.5, 10.3, 9.2; LCMS (4 min ESI) *R*_t_ = 2.73 min, *m*/*z* 486.1804 [M + H]^+^ expected 486.1809 for C_26_H_25_ClN_7_O.

## Supporting Information Available

Molecular formula strings with associated biochemical assay data and calculated properties (CSV)
Supplementary experimental methods: protein production, purification, and crystallography (BCL6 constructs used, methods for expression, purification, and crystallography, including data collection, processing, and refinement), biological assay conditions (methods for TR-FRET, NanoBRET, SPR, and cell proliferation assays), physicochemical assay conditions (methods for NMR and HPLC), description of a method for analysis of water molecules using SZMAP, in vivo PK experimental methods; Supplementary Table S1: crystallographic data collection and refinement statistics, Supplementary Tables S2 and S3: individual values and summary statistics for TR-FRET and NanoBRET assays, Supplementary Table S4: SPR sensorgrams and Langmuir curves for **25**, Supplementary Table S5: PK parameters for individual animals taken from the pharmacokinetic study of **25**; Supplementary Figures S1 and S2: X-ray structures of BCL6 with **8e** and **12e**, Supplementary Figure S3: hydrogen-bond angles and distances from X-ray structures of BCL6 with **8f, 12b, 13e, 13g, 25**, Supplementary Figure S4: concentration vs time plot from PK study with **25**, Supplementary Figure S5: curves from a 14 day cell viability assay (PDF)

## Accession Codes

Atomic coordinates and structure factors for the crystal structures of BCL6 with compounds **2, 8c, 8e, 8f, 12b, 12c, 12e, 13e, 13g**, and **25** can be accessed using PDB codes 7OKE, 7OKF, 7OKG, 7OKH, 7OKI, 7OKJ, 7OKK, 7OKL, 7OKM, and 7OKD, respectively. The authors will release the atomic coordinates and experimental data upon article publication.

## Abbreviations Used

DIPEA: *N*,*N*-diisopropylethylamine
DLBCL: diffuse large B-cell lymphoma
TR-FRET: time-resolved fluorescence energy transfer
NanoBRET: a BRET-based assay format commercialized by Promega, which uses NanoLuc luciferase to generate the donor signal and a HaloTag ligand as the fluorescent energy acceptor
