# Supplementary material for: Into Deep Water: Optimizing BCL6 Inhibitors by Growing into a Solvated Pocket
Source: J Med Chem. 2021 Nov 30;64(23):17079–97. doi: 10.1021/acs.jmedchem.1c00946 (PMC8667045; doi:10.1021/acs.jmedchem.1c00946)
Supplement: Supplementary file 2 — jm1c00946_si_002.pdf [file jm1c00946_si_002.pdf]

Supporting information

Into deep water: optimising BCL6 inhibitors by growing into a solvated pocket

Matthew G. Lloyd‡, Rosemary Huckvale‡, Kwai-Ming J. Cheung, Matthew J. Rodrigues, Gavin W. Collie #, Olivier A. Pierrat, Mahad Gatti Iou, Michael Carter, Owen A. Davis, P. Craig McAndrew, Emma Gunnell, Yann-Vaï Le Bihan, Rachel Talbot, Alan T. Henley, Louise D. Johnson, Angela Hayes, Michael D. Bright, Florence I. Raynaud, Mirco Meniconi, Rosemary Burke, Rob L. M. van Montfort[#], Olivia W. Rossanese, Benjamin R. Bellenie[†], Swen Hoelder[†]**

AUTHOR ADDRESS

[†]Cancer Research UK Cancer Therapeutics Unit and [#]Division of Structural Biology, The Institute of Cancer Research, London SM2 5NG, U.K.

*E-mail: Benjamin.Bellenie@icr.ac.uk. Phone: +44 (0) 2087224602 (B.B.).

*E-mail: Swen.Hoelder@icr.ac.uk. Phone: +44 (0) 2087224353 (S.H.).

Contents

Contents 2

1. Supplementary experimental: protein production, purification and crystallography 3

1.1 BCL6 constructs used for assays and crystallography 3

1.2 BCL6 expression 3

1.3 BCL6 purification 3

1.4 BCL6 crystallisation 4

1.5 Crystallographic data collection, processing and refinement 4

2. Supplementary experimental: biological assay conditions 6

2.1 TR-FRET assay 6

2.2 NanoBRET assay 6

2.3 SPR assay 7

2.4 Cell proliferation assay 8

3. Supplementary experimental: physicochemical assays 9

3.1 NMR solubility assay 9

3.2 HPLC solubility assay 9

4. Supplementary experimental: in silico experiments 11

4.1 Analysis of thermodynamic properties of water molecules using Openeye SZMAP 11

5. Supplementary experimental: in vivo PK and PD experiments 12

5.1 *In vivo* pharmacokinetic studies 12

6. Supplementary data tables 13

Supplementary Table S1: Crystallographic data collection and refinement statistics. 13

Supplementary Table S2: Individual values and summary statistics for TR-FRET assay. 17

Supplementary Table S3: Individual values and summary statistics for NanoBRET assay. 18

Supplementary Table S4: SPR sensorgrams and Langmuir curves 18

Supplementary Table S5: Data (individual replicates) from pharmacokinetic study of ***25****.* 19

7. Supplementary figures 20

Supplementary Figure S1: Alternative binding conformations of ***8e*** *shown by X-ray* 20

Supplementary Figure S2: X-ray structure of ***12e*** *bound to the BTB domain of BCL6.* 20

Supplementary Figure S3: Alternative view of X-ray structures of ***8f*** *showing key measurements.* 21

Supplementary Figure S4: Concentration:time curves from PK study with ***25*** 24

Supplementary Figure S5: Curves from 14-day cell viability assays with compound ***25*** 25

8. Supplementary analytical data 26

8.1 LCMS traces for key compounds 26

9. **References for supplementary information** 32

Supplementary experimental: protein production, purification and crystallography

1.1 BCL6 constructs used for assays and crystallography

A first construct of BCL6 BTB domain, which we will refer to as Trx-6His-HRV3C-BCL6, was obtained by sub-cloning the sequence coding for residues 5-129 of human BCL6, corresponding to its BTB domain, into a pET48b vector with N-terminal Thioredoxin and 6-Histidine tags, followed by a HRV-3C protease cleavage site. For the TR-FRET assay, the Trx-6His-HRV3C-BCL6 protein construct was used without cleaving the tag, as a 6His was needed to bind to the anti-6His-Terbium antibody. For the FP assay and crystallography with compounds 2, 8c, 8e, 8f, 12b, 12c, 12e, 13e and 13g, the tag was removed by HRV-3C protease treatment, generating a simpler BCL6 5-129 product.

For crystallography with compound 25, the construct described above was modified to introduce a Flag Tag and a TEV cleavage site between the HRV3C and BCL6 sequences. This construct will be referred to as Flag-TEV-BCL6.

1.2 BCL6 expression

For both plasmid constructs described above, transformed BL21-AI E. coli cells were grown in LB media supplemented with 50 mg/L kanamycin at 37 ^{o}C until an OD600 nm of 0.6 was reached. Protein expression was then induced by addition of 0.2 mM IPTG and 0.2 % Arabinose. Expression was carried out at 18 ^{o}C for 18 hours. Cells were harvested by centrifugation (5500 g for 30 minutes at 4 ^{o}C) and stored at -80 ^{o}C.

1.3 BCL6 purification

Cells were re-suspended in a buffer composed of 20 mM Tris pH 8, 250 mM NaCl, 1 mM $MgCl_2$, 0.5 mM TCEP and 5 % glycerol, 1x cOmplete^{TM} ULTRA protease inhibitors and 12.5 U/ml Benzonaze. Cells were lysed by sonication followed by centrifugation at 21,000 g for 45 minutes at 4 ^{o}C. The supernatant was loaded onto a HisTrap FF column followed by on-column cleavage of the Trx-6His-HRV3C tag by addition of 2 mg of HRV-3C protease. The cleaved BCL6 5-129 BTB domain or Flag-TEV-BCL6 5-129 was then eluted and purified further by ResourceQ (for Flag-TEV-BCL6 construct only) and gel filtration using a HiLoad 26/60 Superdex75 column in a buffer containing 20 mM HEPES pH 7.5, 250 mM NaCl, 1 mM TCEP and 5 % glycerol. The final protein was assessed for purity and molar mass by SDS-PAGE and high-resolution mass spectrometry, respectively.

For the uncleaved Trx-6His-HRV3C-BCL6 protein construct to be used in TR-FRET, the protein was directly eluted from the HisTrap FF column without HRV-3C treatment, and submitted to Superdex75 gel filtration as described above.

1.4 BCL6 crystallisation

The purified BCL6 5-129 was crystallised in the presence of a tetra-peptide of sequence Ac-WVIP-NH_2. A stock solution of WVIP peptide at 100 mM in 100 % DMSO was added to a 2 mg/mL solution of purified BCL6 to a final concentration of 1 mM. This mixture was then concentrated to a final protein concentration of 4 mg/mL using a centrifugal concentrator with a 3 kDa molecular weight cut-off. Crystals were grown at 18 ^{o}C in hanging drops composed of 2 μL of the BCL6-BTB/WVIP complex plus 1 μL of a crystallisation solution consisting of 1 M K_2HPO_4, 0.7 M NaH_2PO_4, 75 mM sodium acetate buffer pH 4.5 and 2 % DMSO, against 350 μL of crystallisation solution. Crystals typically grew in 2 days, and compounds were soaked into crystals by addition of 0.5 μL of each compound (dissolved in DMSO to a final concentration of 10 to 200 mM) directly to crystallisation drops, followed by 10-120 minutes incubation. Crystals were then cryo-protected in a solution composed of the crystallisation reagent supplemented with 30 % ethylene glycol and cryo-cooled in liquid nitrogen.

The purified Flag-TEV-BCL6 5-129 was crystallised without any peptide, the supplementary Flag-TEV tag of this construct replacing the WVIP peptide in the crystal packing. The protein was concentrated to a final protein concentration of 10 mg/mL using a centrifugal concentrator with a 3 kDa molecular weight cut-off. Crystals were grown at 18 ^{o}C in hanging drops composed of 1.5 μL of the Flag-TEV-BCL6 complex plus 1.5 μL of a crystallisation solution consisting of 0.1 M Tris pH 7.5 and 0.80 M Na/K Tartrate, against 300 μL of crystallisation solution. Crystals typically grew in 2 days, and compounds were soaked as described above for the other construct. Crystals were then cryo-protected in a solution composed of the crystallisation reagent supplemented with 30 % ethylene glycol and cryo-cooled in liquid nitrogen.

1.5 Crystallographic data collection, processing and refinement

X-ray data were collected at Diamond Light Source, Harwell campus, Oxfordshire, UK, on beamlines I03, and I04-1, at ESRF, Grenoble, France, on beamline ID30A-1, or in-house at the Institute of Cancer Research, London, UK, on a Rigaku FRX-AFC11-VariMax Cu-VHF-Pilatus300K. Crystals obtained with both BCL6 constructs belonged to the space group P 6_1 2 2 and diffracted to between 1.25 and 2.05

Å resolution. Datasets were integrated with XDS[1] or DIALS[2] and scaled and merged with AIMLESS[3] or AutoPROC.[4] Structures were solved by molecular replacement using PHASER[5-6] with a publicly available BCL6 structure[7] (PDB code 3BIM) with ligand and water molecules removed used as molecular replacement model. All protein/ligand structures were manually corrected and rebuilt in COOT[8] and refined with BUSTER[9] in iterative cycles. Ligand restraints were generated with GRADE[10] and MOGUL[11]. The quality of the structures was assessed with MOLPROBITY[12-13]. The data collection and refinement statistics are presented in Supplementary Table S1.

2. Supplementary experimental: biological assay conditions

Cell lines were supplied by the German Collection of Microorganisms and Cell Cultures (DSMZ). Cell lines were authenticated by STR profiling using a GenePrint® 10 kit (Promega, Southampton, UK) and a 3730xl DNA analyser (Applied Biosystems, Warrington, UK). All STR profiles were >80% match (using ATCC or DSMZ matching algorithms) with the respective reference profile. Cells were routinely screened for *Mycoplasma*, using an in-house PCR-based assay (Universal Mycoplasma Detection Kit (30-1012K, ATCC, Manassas, VA, USA).

2.1 TR-FRET assay

Assays were performed in a 384-well black Proxiplate (Perkin Elmer) containing 1 nM* Trx-6xHis-BCL6 (in house-produced, human BCL6 BTB domain covering amino-acid sequence 5-129), 300 nM BCOR-AF633 peptide (RSEIISTAPSSWVVPGP-Cys-AlexaFluor 633-amide, Cambridge Research Biochemical) and 0.5 nM anti-6xHis-Terbium cryptate (CisBio Bioassays, France), in assay buffer (25 mM Hepes pH8, 100 mM NaCl, 0.05% Tween20, 0.5 mM TCEP, 0.05% bovine serum albumin). Test compounds in DMSO or DMSO alone were added to the wells using an ECHO550 acoustic dispenser (Labcyte Inc) to give the appropriate test concentration in 0.7% v/v DMSO final. After 2 hours incubation at room temperature the plate was read on an Envision plate reader (Perkin Elmer) with 337 nm laser excitation, a first emission filter APC 665 nm and a second emission filter Europium 615 nm, or alternatively on a Pherastar FSX (BMG Labtech) plate reader equipped with 337 nm laser excitation filter, a first emission filter at 620 nm and a second emission filter at 665 nm. The % inhibition at each concentration was calculated by normalising FRET ratio to the appropriate high (DMSO with all reagents) and low (DMSO without BCL6) controls. IC_{50} values were determined using GraphPad Prism 6.0 or Dotmatics (Bishops Stortford, UK) software by fitting the normalised data to a sigmoidal four-parameter logistic fit equation.

*10 nM was used for early examples, as indicated in data tables.

2.2 NanoBRET assay

A cellular nano-Bioluminescence Resonance Energy Transfer (nanoBRET) assay (Promega NanoBRET Nano-Glo Detection System, catalogue number N1662) was used to detect inhibition of the BCL6-SMRT (also called NCOR2) corepressor protein-protein interaction. DNA encoding full length BCL6 and SMRT were inserted into pFC32K.NanoLuc and pFC14K.HaloTag vectors (Promega) to produce C-terminal tagged fusion proteins BCL6-nanoLuc and SMRT-HaloTag, respectively.

HEK293T cells were plated (5x10^5) in T75 tissue culture flask and bulk transfected 48 hours later with Fugene 6 (Promega cat.# E2691) reagent and 18 µg total DNA plasmids encoding BCL6-nanoLuc as donor and SMRT-HaloTag as acceptor, at a donor:acceptor DNA ratio of 1:25. At 24 hr post-transfection, HEK293T cells were collected and stored in liquid nitrogen in 90% FBS (PAN Biotech UK) and 10% DMSO. At the time of assay, compounds (100nL/well) and NanoBRET 618 ligand (10nL of 1mg/ml stock solution per well) were dispensed in a dry 384-well NUNC white assay plate (ThermoScientific NUNC cat.#10080681) using Echo550 acoustic dispensing (Labcyte Inc.). Frozen transfected HEK293T cells were thawed, centrifuged and freezing medium was replaced by phenol red-free OptiMEM+4% FBS (Life Technology). The cell density was adjusted to 3x10^5 cells/ml and 20 µL (6000 cells) were plated in each well containing test compounds (0.0125-50 µM) in DMSO or DMSO alone and 0.5 µg/ml NanoBRET 618 fluorescence ligand, in 0.55% v/v DMSO final concentration. Cells were incubated for 6 hr at 37 °C / 5% CO_2 then NanoBRET furimazine substrate (Promega) was added to give a final concentration of 10 µM. After a short centrifugation the plates were read on an Envision (Perkin Elmer) plate reader equipped with a LUM/D600 Dual mirror, Lum 450/40 nm bandpass and D605 nm longpass filters, with a 0.2 sec reading to determine the BRET ratio. Alternatively, plates were read on Pherastar FSX (BMG Labtech) equipped with BRET module LP610 nm (1st emission filter) / 450-80 nm (2nd emission filter). The % inhibition at each test concentration was calculated by normalising the BRET ratio to the appropriate high and low controls. The compound IC_{50}s were determined using Graphpad Prism 6.0 or Dotmatics software by fitting the normalised data to a sigmoidal four-parameter logistic fit equation.

2.3 SPR assay

Surface plasmon resonance (SPR) experiments were carried out on a Biacore T200 (GE Healthcare Life Sciences) and amine coupling chemistry was used to immobilise the protein on a research grade CM5 sensor chip. The running buffer was 100mM sodium acetate, 100mM sodium chloride, 1mM TCEP pH6.0 and the chip's surface was activated for 10min using a 1:1 mixture of 100mM N-hydroxysuccinimide and 400mM 1-ethyl-3-(3-dimethylaminopropyl)-carbodiimide. BCL6 BTB protein was injected for 20min at a concentration of 100µg/mL in a 10mM sodium acetate buffer pH5.5. Finally, the surface was blocked via an injection of 1M ethanolamine pH8.5 for 7min. The flow rate was maintained at 10µL/min for all the above procedures and ~1500 response units (RU) of BCL6 was immobilised on the chip. Flow cell one was left unmodified as the reference surface.

Following protein immobilisation, the running buffer was changed to 100mM sodium acetate, 100mM sodium chloride, 1mM TCEP, 0.05% Tween20 (v/v), 5% DMSO pH6.0.

All compound handling was done on an ECHO 550 acoustic liquid dispenser (Labcyte) and compounds were added to 384-well polypropylene V-bottomed plates (Greiner), which became the sample plates for the SPR. For KD determinations, an eight-point concentration range was generated as shown, with a final DMSO concentration of 4%. The flow rate was 30µL/min, the injection time for the samples was 60 secs and the dissociation time was 60 sec. The surface was not regenerated between sample injections. KD values were calculated from the Langmuir plot under equilibrium conditions using the 1:1 binding model in the Biacore software version 2 (GE Life Sciences, Amersham Place, UK).

2.4 Cell proliferation assay

Cells were seeded in 96-well culture plates at a density of 2500 cells/well in RPMI-1640 medium (Sigma-Aldrich) supplemented with 10% FBS (Gibco). Compounds were initially dispensed into 96-well U-bottom plates using an Echo 550 acoustic dispenser (Labcyte Inc.), then diluted in RPMI-1640 medium and transferred onto the cells. Cells were treated with 8 compound concentrations in duplicate, ranging from 1.07 nM to 10 µM, in a final DMSO concentration of 0.1% and final volume of 100 µl. Cells were incubated with compound for 14 days, with medium changes at days 3, 7 and 10 carried out as follows: fresh 96-well cell culture plates were prepared containing 100 µl medium plus compound at the assay concentrations (white plates were used on day 10 to optimise luminescence measurement). Assay plates containing cells were vortexed to mix and cell density in one control well was counted using a Coulter Z2 cell counter (Beckman Coulter). The volume of medium containing 2500 cells in the control well was calculated and this volume of cells was transferred from every well of the assay plates to the corresponding well of the fresh plates containing compound. After 14 days, CellTiter Glo reagent (Promega) was added to the medium in each well of the assay plate at a ratio of 1:2, mixed on a plate shaker, then incubated at room temperature for 10 minutes. Luminescence was measured using an Envision plate reader (Perkin Elmer) and the relative luminescence at each compound concentration, compared to DMSO alone, was calculated. GI50 were determined using a 4-parameter curve fit in Dotmatics (Bishops Stortford, UK).

3. Supplementary experimental: physicochemical assays

3.1 NMR solubility assay

9 µL of 10 mM DMSO stock solution was pipetted into one well of a 384 deepwell plate (Greiner, part-no. 781270), then 171 µL of HEPES buffer (20 mM HEPES [Sigma Aldrich, cat-no. H3375-250G], 150 mM NaCl, 0.5 mM TCEP, 10% D_2O) was pipetted into the same well and mixed by up-down pipetting 3 times to create a 0.5 mM solution or suspension containing 5% DMSO. It was then separated by centrifugation (1000 rpm for 1 min, Eppendorf 5810C). The plate was then sealed and incubated at room temperature for 20 hours, without shaking. The plate was centrifuged again for 1 minute at 1000 rpm on Eppendorf 5810R before 165 µL of the supernatant was transferred to a 3 mm NMR tube (Bruker, Part No. Z112272) using liquid handler SamplePro Tube SJ S (Bruker). The concentration of the solubilized compound in solubility sample is measured by quantitative ^{1}H-NMR using a single external standard (200 µM caffeine (Sigma, C1778) in PBS (pH 7.4) with 1% DMSO-d6).

The detail of the NMR method is as following: NMR data was collected on a Bruker Avance Neo 600 spectrometer equipped with a 5 mm TCI-CryoProbe. The ^{1}H spectrum was referenced to the internal deuterated solvent. The operating frequency for ^{1}H was 600 MHz. All NMR data were acquired at the temperature of 298 K. All data were acquired and processed using Bruker Topspin 4.0. The quantitative ^{1}H-NMR spectrum was acquired using a Bruker standard 1D lc1pngppsf2 pulse sequence with 32 scans. The sweep width was 6.2 ppm with O1P set to 8.8 ppm, and the FID contained 16k time-domain data points. Relaxation delay was set to 20 sec. Water signal was suppressed. [14-16]

3.2 HPLC solubility assay

10 µL of 10 mM DMSO stock solution was pipetted into a micro centrifuge tube (1.5 mL, Sarstedt part-no. 72.690.001) containing 990 µL of PBS buffer (pH 7.4, Sigma Aldrich, cat-no. 79382) and mixed for 5 seconds on vortex mixer (Grant-bio vortex mixer) to create 100 µM solution with 1% DMSO. Following shaking of the suspension on a Bohdan Shaker at 500 rpm for 2 hours at room temperature (20 °C), it was separated by centrifugation (14000 rpm for 15 min, Eppendorf 5415C). 200 µL of the supernatant was transferred to a 2 mL Agilent vial containing 50 µL of DMSO (Sigma Aldrich, cat-no. 41640-100ML) and mixed for 5 seconds to avoid precipitation from the saturated solution.

The concentration of the solubilized compound in solubility sample is measured by HPLC with UV detection using an external standard which was prepared by pipetting 10 μL of the same batch of compound DMSO stock used in solubility sample preparation to 990 μL of DMSO.

The detail of the HPLC method is as following: chromatographic separation at 30°C is carried out over a 5 minute gradient elution method from 90:10 to 10:90 water:methanol (both modified with 0.1% formic acid) at a flow rate of 1.5 mL/min. Calibration curve is prepared by injecting 0.5, 2.5, and 5 μL of compound external standard. Compound solubility value is obtained by injecting 6.25 and 62.5 μL of compound solubility sample.

4. Supplementary experimental: in silico experiments

4.1 Analysis of thermodynamic properties of water molecules using Openeye SZMAP.

Analysis of thermodynamic properties of water molecules using Openeye SZMAP.

In the work described in this manuscript, we have used the Openeye SZMAP software [1] to analyze thermodynamic properties of water molecules in the binding site of BCL6, in the presence of several ligands, and to assess how different ligands can increase biological activity by favourably perturbating the water network around them. In particular, we used SZMAP to estimate the change in the relative free energy (called SZMAP neutral probe free energy difference, or simply $\Delta\Delta G$) when a water molecule observed in a crystal structure is replaced, computationally, by a hydrophobic probe. A positive $\Delta\Delta G$ calculated at the coordinate of a particular water molecule indicates that a hydrophobic probe would be better tolerated in that position. On the contrary, a negative $\Delta\Delta G$ indicates that the water molecule is sitting in a hydrophilic spot. Calculated $\Delta\Delta G$ values in the region of -0.5 to +0.5 kcal/mol reflect a preference for neither polar nor hydrophobic probes. Work published in 2015 by Astra-Zeneca shows that the SZMAP calculated thermodynamic parameter correlates well with analogue results from more sophisticated computational methods as well as experimental data [2].

We carried out SZMAP water analyses on several BCL6:ligand complexes of interest, described in the *results* section. The PDB structures of the BCL6:ligand complexes used in our calculations were resolved in house. Hydrogen atoms positions and protonation states were then determined using the protonate-3D procedure implemented in MOE v2019 [3,4] using the default options and the GB/VI electrostatics. To estimate the $\Delta\Delta G$ we used SZMAP v1.2.1.4 with all the default options. All the calculated $\Delta\Delta G$ are provided as kcal/mol.

1. SZMAP; OpenEye Scientific Software Inc., Santa Fe, NM, USA, 2011

2. Bayden, A. S.; Moustakas, D. T.; Joseph-McCarthy, D.; Lamb, M. L. Evaluating Free Energies of Binding and Conservation of Crystallographic Waters Using SZMAP. *Journal of chemical information and modeling* **2015**, *55* (8), 1552–1565. https://doi.org/10.1021/ci500746d.

3. Molecular Operating Environment (**MOE**), 2019.01; Chemical Computing Group ULC, 1010 Sherbooke St. West, Suite #910, Montreal, QC, Canada, H3A 2R7, 2021.

4. Labute, P. Protonate3D: Assignment of ionization states and hydrogen coordinates to macromolecular structures. *Proteins: Structure, Function, and Bioinformatics* **2009**, *75* (1), 187–205. https://doi.org/10.1002/prot.22234.

5. Supplementary experimental: in vivo PK and PD experiments

All procedures were in accordance with UK Home Office regulations under the Animals (Scientific Procedures) Act 1986, approved by The Institute of Cancer Research's Ethics Committee and in accordance with published guidelines[17]. Mice were allowed access to food and water *ad libitum*.

5.1 *In vivo* pharmacokinetic studies

All experiments were conducted in accordance with the UKCC guidelines for animal experimentations[17]. Animals were adapted to laboratory conditions for at least 1 week prior to dosing and were allowed food and water *ad libitum*. **CCT369347 (25)** was administered iv or po (mouse, 0.1 mL/10 g in 10% DMSO, 5% tween 20 in saline); Blood samples were collected in heparinised capillaries from the tail vein at 8 time points over the 24 h post dose and frozen on collection together with a standard curve and quality controls spiked in control blood. Samples were reconstituted in a Water:MeOH mixture containing internal standard as previously described (Roberts et al, 2016). Following centrifugation, extracts were analyzed by multiple reaction monitoring of precursor and product ions by ESI-LCMS/MS on either a Waters (Milford, MA, USA) Xevo TQ-S or Sciex (Framingham, MA, USA) QTrap6500 following gradient separation with 0.1% formic acid and methanol on a Phenomenex (Macclesfield, UK) Kinetex C18 UPLC column (50 × 2.1 mm, 2.6 μM). Quantitation was carried out with an external calibration. Quality controls were included and were within 20% of nominal concentration. Pharmacokinetic parameters were derived from noncompartmental analysis using Phoenix Pharsight. Non compartmental analysis (model 200 and 201) version 6.3.

6. Supplementary data tables

Supplementary Table S1: Crystallographic data collection and refinement statistics.

Crystal system	BCL6/WVIP	BCL6/WVIP	BCL6/WVIP
Ligand	**2**	**8c**	**8e**
PDB code	7OKE	7OKF	7OKG
Crystal			
Space group	P 6_1 2 2	P 6_1 2 2	P 6_1 2 2
Unit cell dimensions (a/b/c in Å)	67.13/67.13/164.89	68.49/68.49/167.48	67.24/67.24/165.47
Unit cell angles (α/β/γ in °)	90/90/120	90/90/120	90/90/120
Data collection and processing			
Beamline	ESRF ID30A-1	In-house Rigaku	ESRF ID30A-1
Wavelength (Å)	0.9660	1.5419	0.9660
Integration program	XDS	DIALS	XDS
Reduction program	AIMLESS	AIMLESS	AIMLESS
Resolution range	47.51 – 1.48	33.72 – 1.60	41.37 – 1.32
Number of unique reflections [a]	37651 (3631)	30154 (1500)	53022 (2508)
Completeness [a]	100 (100)	99.9 (99.5)	99.9 (97.8)
Redundancy [a]	19.1 (18.4)	14.4 (8.9)	12.2 (11.5)
R_{merge} (%) [a]	11.0 (258.5)	9.6 (133.7)	9.8 (216.9)
I/σ(I) [a]	17.7 (1.3)	16.5 (1.6)	13.2 (1.3)
$CC_{1/2}$ [a, b]	0.999 (0.511)	0.998 (0.409)	0.999 (0.470)
Refinement			
Program	BUSTER	BUSTER	BUSTER
R_{work} (%)	16.82	16.67	16.92
R_{free} (%)	18.66	17.80	17.95
Number of residues	131	131	131
Number of water molecules	188	204	195
Average B-factor ($Å^2$)	28.34	22.32	22.91
Ramachandran favoured (%)	97.62	96.83	98.88
Ramachandran outliers (%)	0	0	0
RMSD bonds (Å)	0.016	0.014	0.012
RMSD angles (°)	1.520	1.540	1.483

[a] Values in parentheses are for the highest resolution shell.

[b] Half-dataset correlation coefficient, see: Karplus, P. A.; Diederichs, K. Linking crystallographic model and data quality. *Science* **2012**, *336*, 1030−1033.

Crystal system	BCL6/WVIP	BCL6/WVIP	BCL6/WVIP
Ligand	**8f**	**12b**	**12c**
PDB code	7OKH	7OKI	7OKJ
Crystal			
Space group	P 6_1 2 2	P 6_1 2 2	P 6_1 2 2
Unit cell dimensions (a/b/c in Å)	66.45/66.45/151.20	67.90/67.90/166.03	67.47/67.47/165.49
Unit cell angles (α/β/γ in °)	90/90/120	90/90/120	90/90/120
Data collection and processing			
Beamline	ESRF ID30A-1	ESRF ID30A-1	ESRF ID30A-1
Wavelength (Å)	0.9660	0.9660	0.9660
Integration program	XDS	XDS	XDS
Reduction program	AIMLESS	AUTOPROC	AUTOPROC
Resolution range	41.40 – 1.52	58.80 – 1.61	58.43 – 1.43
Number of unique reflections [a]	35422 (1701)	29885 (1392)	41944 (2043)
Completeness [a]	100 (100)	98.5 (93.8)	100 (99.3)
Redundancy [a]	13.6 (13.4)	7.3 (7.7)	11.1 (9.5)
R_{merge} (%) [a]	19.2 (268.7)	10.8 (80.5)	9.3 (109.4)
I/σ(I) [a]	10.9 (1.2)	12.3 (2.5)	13.7 (2.2)
$CC_{1/2}$ [a, b]	0.997 (0.553)	0.998 (0.391)	0.999 (0.475)
Refinement			
Program	BUSTER	BUSTER	BUSTER
R_{work} (%)	18.07	16.22	16.54
R_{free} (%)	19.42	18.89	17.68
Number of residues	130	131	131
Number of water molecules	199	153	198
Average B-factor (Å^2)	24.68	28.41	24.60
Ramachandran favoured (%)	97.60	98.41	98.90
Ramachandran outliers (%)	0	0	0
RMSD bonds (Å)	0.014	0.014	0.011
RMSD angles (°)	1.526	1.551	1.469

[a] Values between brackets are for the highest resolution shell.

[b] Half-dataset correlation coefficient, see: Karplus, P. A.; Diederichs, K. Linking crystallographic model and data quality. *Science* **2012**, *336*, 1030−1033.

Crystal system	BCL6/WVIP	BCL6/WVIP	BCL6/WVIP
Ligand	**12e**	**13e**	**13g**
PDB code	7OKK	7OKL	7OKM
Crystal			
Space group	P 6_1 2 2	P 6_1 2 2	P 6_1 2 2
Unit cell dimensions (a/b/c in Å)	67.35/67.35/164.50	68.28/68.28/168.17	68.49/68.49/167.48
Unit cell angles (α/β/γ in °)	90/90/120	90/90/120	90/90/120
Data collection and processing			
Beamline	In-house Rigaku	DLS I04-1	DLS I04-1
Wavelength (Å)	1.5419	0.9282	0.9282
Integration program	XDS	DIALS	DIALS
Reduction program	AIMLESS	AIMLESS	AIMLESS
Resolution range	47.58– 2.05	48.37 – 1.25	48.40 – 1.48
Number of unique reflections [a]	14393 (1070)	64768 (3134)	39743 (1924)
Completeness [a]	98.7 (97.1)	99.7 (99.2)	100 (99.9)
Redundancy [a]	10.3 (6.4)	12.4 (12.8)	12.2 (11.4)
R_{merge} (%) [a]	24.9 (179.9)	6.3 (217.2)	6.3 (260.1)
I/σ(I) [a]	8.2 (1.4)	15.4 (1.2)	15.5 (0.9)
$CC_{1/2}$ [a, b]	0.982 (0.364)	1.000 (0.503)	1.000 (0.472)
Refinement			
Program	BUSTER	BUSTER	BUSTER
R_{work} (%)	19.76	18.75	18.71
R_{free} (%)	22.37	19.72	20.56
Number of residues	129	130	131
Number of water molecules	132	197	194
Average B-factor (Å^2)	21.55	23.60	34.33
Ramachandran favoured (%)	99.02	98.40	98.41
Ramachandran outliers (%)	0	0	0
RMSD bonds (Å)	0.012	0.013	0.015
RMSD angles (°)	1.650	1.413	1.505

[a] Values between brackets are for the highest resolution shell.

[b] Half-dataset correlation coefficient, see: Karplus, P. A.; Diederichs, K. Linking crystallographic model and data quality. *Science* **2012**, *336*, 1030−1033.

Crystal system	Flag-BCL6
Ligand	**25**
PDB code	7OKD
Crystal	
Space group	P 6_1 2 2
Unit cell dimensions (a/b/c in Å)	67.52/67.52/167.19
Unit cell angles (α/β/γ in °)	90/90/120
Data collection and processing	
Beamline	DLS I03
Wavelength (Å)	0.9762
Integration program	XDS
Reduction program	AIMLESS
Resolution range	47.92 – 1.94
Number of unique reflections [a]	17565 (1159)
Completeness [a]	100 (100)
Redundancy [a]	12.8 (12.4)
R_{merge} (%) [a]	9.3 (230.3)
I/σ(I) [a]	14.1 (1.3)
$CC_{1/2}$ [a, b]	0.999 (0.629)
Refinement	
Program	BUSTER
R_{work} (%)	18.09
R_{free} (%)	19.84
Number of residues	132
Number of water molecules	120
Average B-factor (Å^2)	47.68
Ramachandran favoured (%)	99.23
Ramachandran outliers (%)	0
RMSD bonds (Å)	0.014
RMSD angles (°)	1.636

[a] Values between brackets are for the highest resolution shell.

[b] Half-dataset correlation coefficient, see: Karplus, P. A.; Diederichs, K. Linking crystallographic model and data quality. *Science* **2012**, *336*, 1030−1033.

Supplementary Table S2: Individual values and summary statistics for TR-FRET assay.

	**TR-FRET assay. Italic values indicate using 10nM BCL6, otherwise assay used 1nM BCL6**																	
**No**	**IC50 individual replicates, uM**											**Geo Mean IC50 uM**	**Mean pIC50**	**sd**	**sem**	**n**	**LE**	**LLE**
**1**	3.32	*4.22*	2.23	1.70	9.38	*3.23*	2.47					3.27	5.49	0.24	0.09	7	0.35	3.09
**2**	*2.28*	4.29	4.07	1.38								2.72	5.57	0.23	0.12	4	0.35	2.87
**8a**	5.52	7.01	*4.46*									5.57	5.25	0.10	0.06	3	0.31	3.05
**8b**	*1.57*	*1.24*	*0.92*	*0.54*	*2.32*							1.18	5.93	0.24	0.11	5	0.31	2.83
**8c**	5.77	2.55	1.24	*2.44*	*2.90*							2.64	5.58	0.24	0.11	5	0.28	3.68
**8d**	*2.66*	*2.56*	*1.47*	*2.10*	*2.08*							2.13	5.67	0.10	0.05	5	0.29	3.87
**8e**	*1.52*	2.19										1.82	5.74	0.11	0.08	2	0.30	3.54
**8f**	1.66	0.68	1.95	1.13	1.05	*1.91*						1.31	5.88	0.18	0.07	6	0.34	3.48
**11a**	*0.89*	*0.87*	0.81	0.84	1.12							0.90	6.05	0.06	0.02	5	0.30	3.05
**11b**	1.33	*1.44*	1.81	1.49								1.51	5.82	0.06	0.03	4	0.28	2.32
**11c**	0.87	0.89	0.88									0.88	6.06	0.01	0.00	3	0.29	3.46
**11d**	*0.36*	0.27	0.42	0.35								0.35	6.46	0.08	0.04	4	0.30	3.06
**12a**	*0.62*	*0.51*	0.66	*0.47*	*0.46*	*0.50*	0.74	*0.48*	*0.65*	*0.49*	0.58	0.55	6.26	0.07	0.02	11	0.31	4.66
**12b**	0.24	0.30	0.22	0.30	0.28	0.19	0.26					0.25	6.60	0.07	0.03	7	0.33	5.00
**12c**	3.39	2.07	2.27	3.40	3.90	2.94	1.54					2.67	5.57	0.14	0.05	7	0.28	3.97
**12d**	1.18	0.97	1.10									1.08	5.97	0.04	0.02	3	0.28	3.97
**12e**	*19.94*	*22.77*		*74.07*	*10.19*	22.35						23.81	4.62	0.31	0.14	5	0.24	3.42
**13a**	*1.19*	0.70	0.72	*0.75*	*0.97*	0.55	*0.75*	0.88				0.79	6.10	0.10	0.04	8	0.29	4.30
**13b**	1.95	1.10	*0.97*	0.79	*0.79*							1.05	5.98	0.16	0.07	5	0.28	3.88
**13c**	*0.69*	1.07	*0.68*	1.18	0.84							0.87	6.06	0.11	0.05	5	0.27	4.56
**13d**	0.12	0.40	0.16									0.20	6.70	0.27	0.15	3	0.30	5.20
**13e**	*0.12*	0.09	*0.19*	*0.16*	0.12	*0.17*	*0.11*	0.15	0.16			0.14	6.86	0.11	0.04	9	0.31	4.86
**13f**	0.07	0.09	0.09									0.08	7.08	0.06	0.03	3	0.32	5.08
**17a**	0.41	0.23	0.79									0.42	6.38	0.27	0.16	3	0.26	3.48
**17b**	0.25	0.21	0.64									0.32	6.49	0.26	0.15	3	0.26	3.19
**24a**	0.06	0.07	0.09									0.07	7.14	0.09	0.05	3	0.31	4.54
**24b**	0.05	0.05	0.06									0.05	7.27	0.03	0.02	3	0.30	4.27
**13g**	*0.05*	0.08	*0.05*	0.02	*0.04*	0.06						0.05	7.33	0.20	0.08	6	0.32	5.33
**25**	0.04	0.02	0.03	0.03	0.02							0.03	7.58	0.16	0.07	5	0.30	4.58

Supplementary Table S3: Individual values and summary statistics for NanoBRET assay.

compound	IC50 individual replicates, uM									Geo Mean IC50 uM	pIC50 mean	sd	sem	n
**1**	36.2	41.5								38.75	4.41	0.04	0.03	2
**13e**	1.12	1.02	1.17	1.1	1.59	1.68	0.96	1.12	1.31	1.21	5.92	0.08	0.03	9
**13f**	0.87	2.13	1.79							1.49	5.83	0.21	0.12	3
**17a**	6.82	5.7	2.81							4.78	5.32	0.20	0.12	3
**17b**	2.07	4.64	0.96							2.10	5.68	0.34	0.20	3
**24a**	1.08	1.08								1.08	5.97	0.00	0.00	2
**24b**	0.96	1.52	0.96							1.12	5.95	0.12	0.07	3
**13g**	0.3	0.67	0.5							0.47	6.33	0.18	0.10	3
**25**	0.72	0.51	0.56	0.51						0.57	6.25	0.07	0.04	4

*Supplementary Table S4: SPR sensorgrams and Langmuir curves for **25***

Langmuir	Sensorgram	K_D (nM)	Rmax (RU)
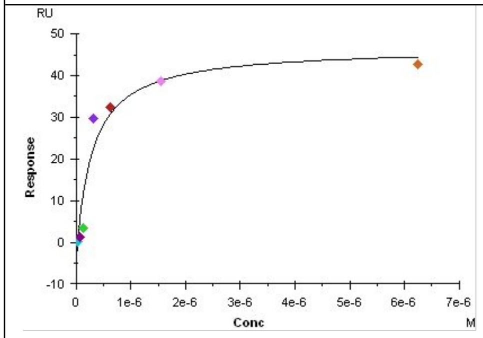	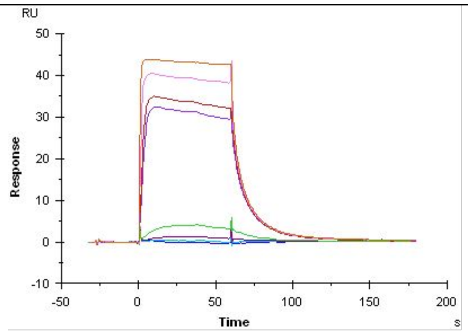	277	52
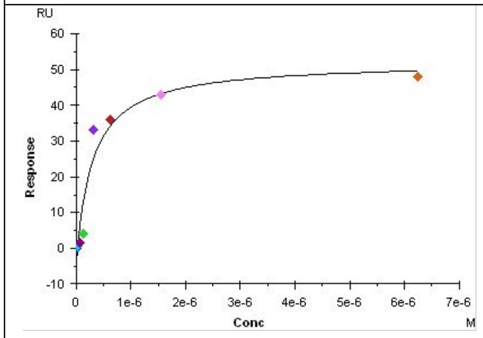	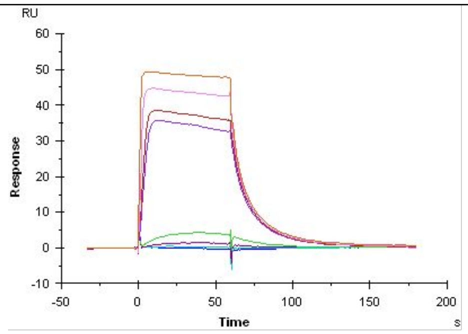	282	58

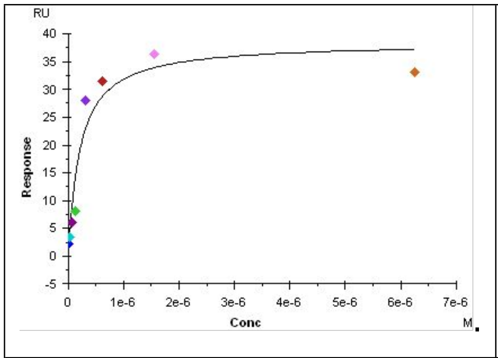	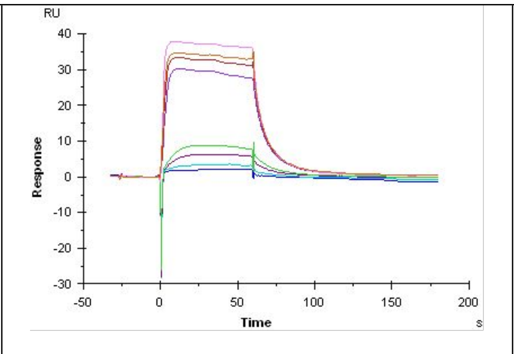	198	40
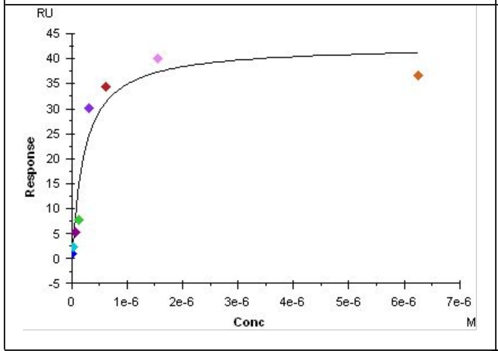	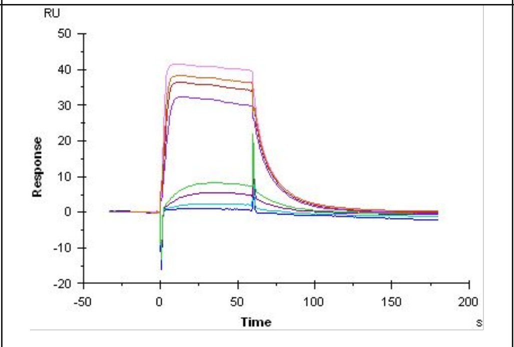	201	46
	Mean values	236	49
	pKD +/- SEM	6.63 ± 0.04	

Supplementary Table S5: Data (individual replicates) from pharmacokinetic study of ***25****.*

Route	Dose (mg/kg)	Animal	Animal Wt (g)	Tmax (h)	Cmax (nmol/L)	AUClast (h*nmol/L)	AUCINF (h*nmol/L)	CL (ml/min/kg)	HL_Lambda_z (h)	Vss (L/kg)	F* AUClast	F* AUCINF
IV	1	2	18.6	0.08	521	1275	1376	24.9	7.99	9.43	-	-
		3	18.9	0.08	471	1395	1450	23.7	5.93	6.96		
		4	19.4	0.08	443	1344	1416	24.2	6.83	7.81		
PO	5	6	18.6	2	347	1352	1435	24.5	1.33	-	0.29	0.30
		7	20.0	2	382	1534	1848	19.0	2.12			
		8	19.9	1	427	1233	1279	27.5	1.14			

** assuming linear PK, calculated from AUC6h*

7. Supplementary figures

*Supplementary Figure S1: Alternative binding conformations of **8e** shown by X-ray*

A B

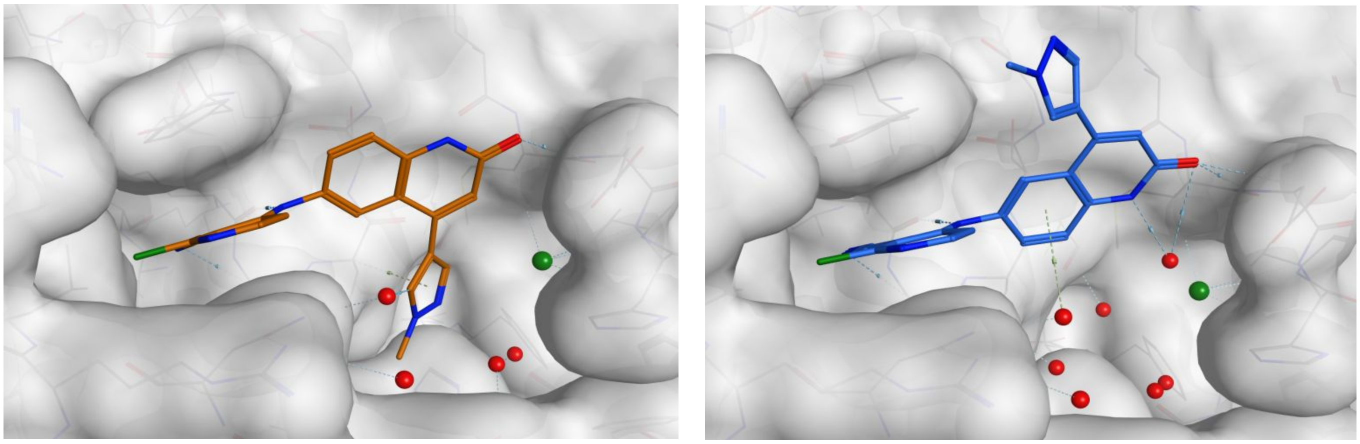

X-ray structure of **8e** *shown in its two alternate conformations, pocket-facing (A) and solvent-facing (B), each showing associated water molecules in extended pocket. PDB: 7OKG*

*Supplementary Figure S2: X-ray structure of **12e** bound to the BTB domain of BCL6.*

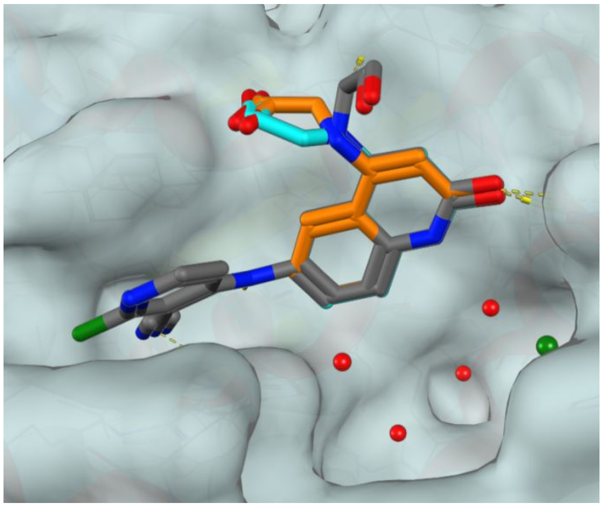

X-ray structure of **12e** *showing ligand bound in the 'solvent-facing' orientation, with associated water molecules in extended pocket. Atoms from the pendant amide group which were poorly stabilised are omitted here for clarity. Three different conformations of the pendant amide group are observed, shown in grey, orange and cyan. PDB: 7OKK*

*Supplementary Figure S3: Alternative view of X-ray structures of **8f** showing key measurements.*

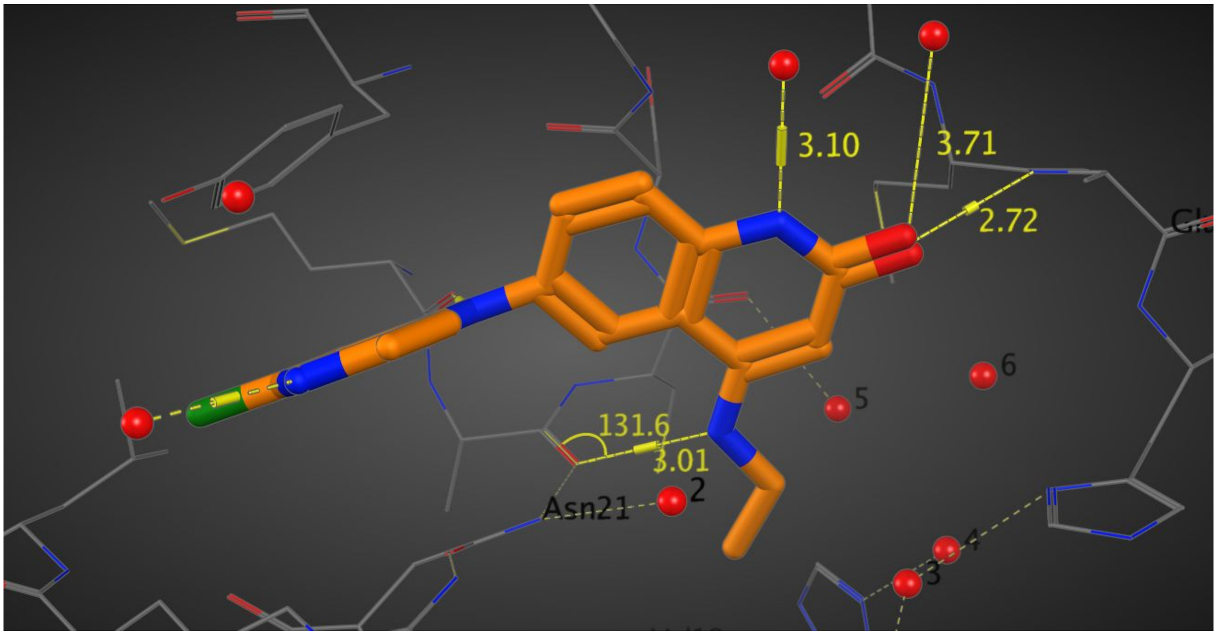

Compound 8f (PDB: 7OKH)

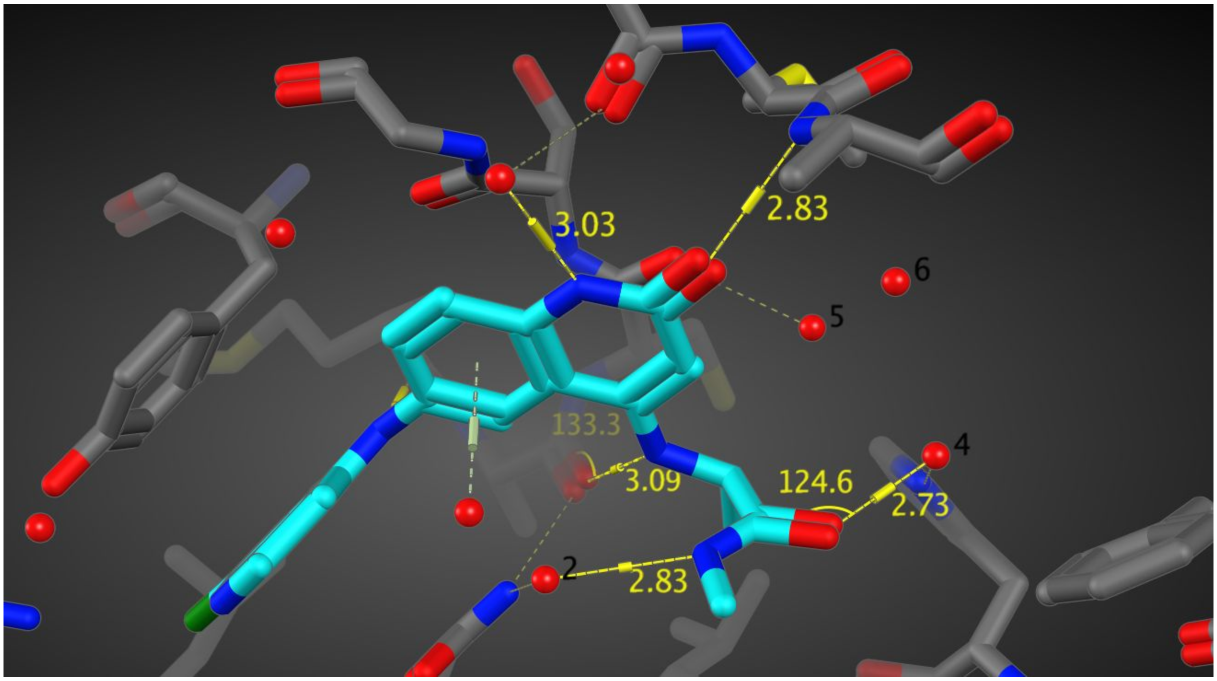

Compound 12b (PDB: 7OKI)

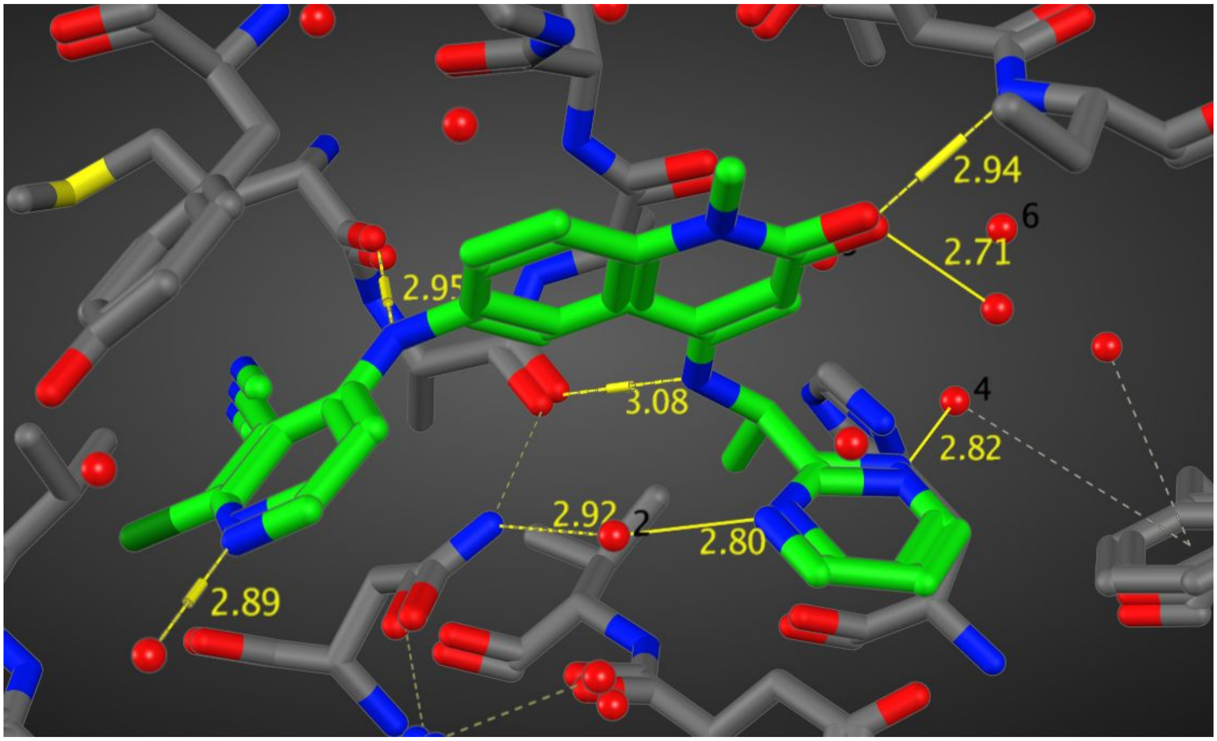

Compound 13e (PDB: 7OKL)

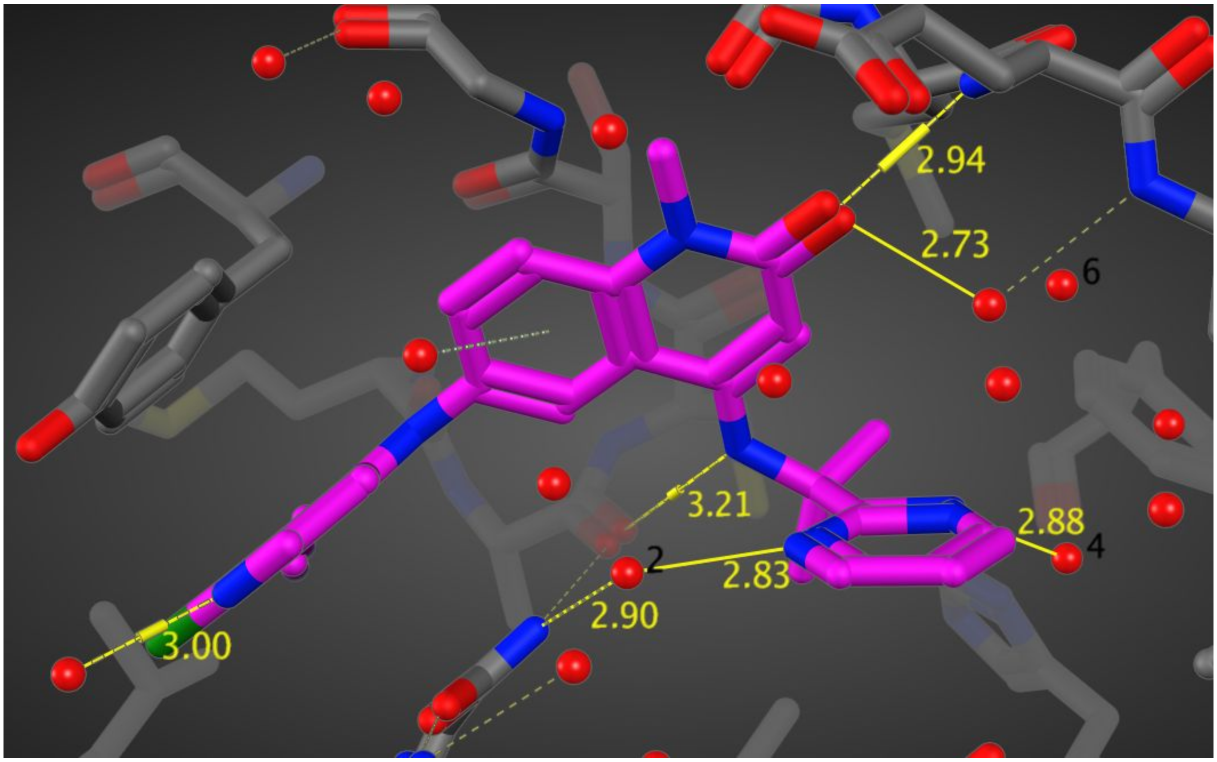

Compound 13g (PDB: 7OKG)

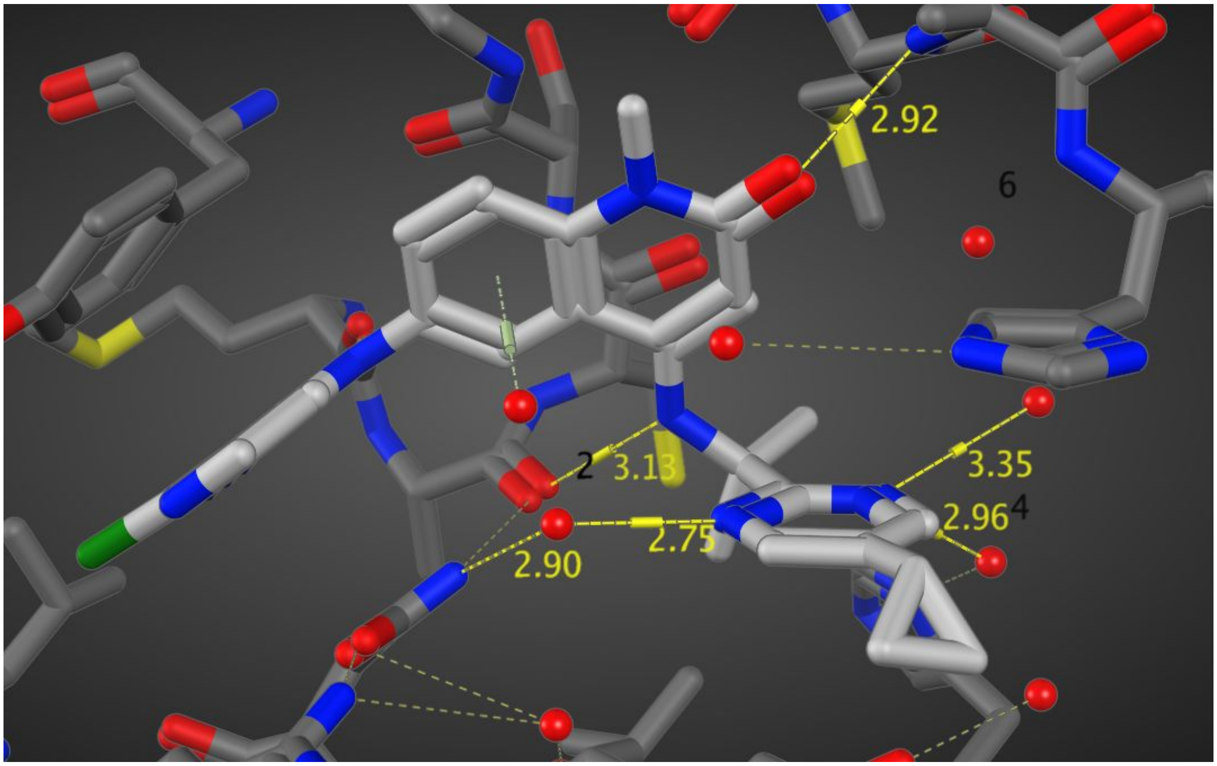

Compound 25 (PDB: 7OKD)

*Supplementary Figure S4: Concentration:time curves from PK study with **25***

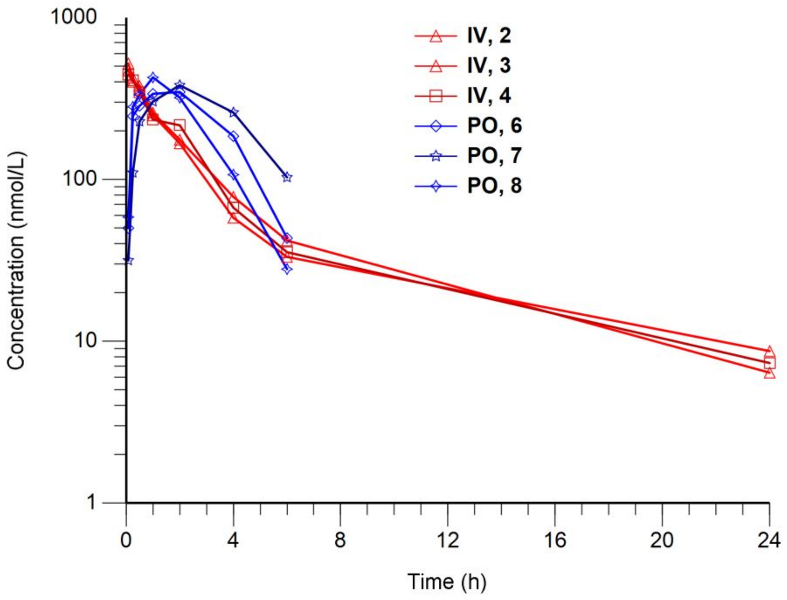

Supplementary Figure S5: Curves from 14-day cell viability assays with compound ***25***

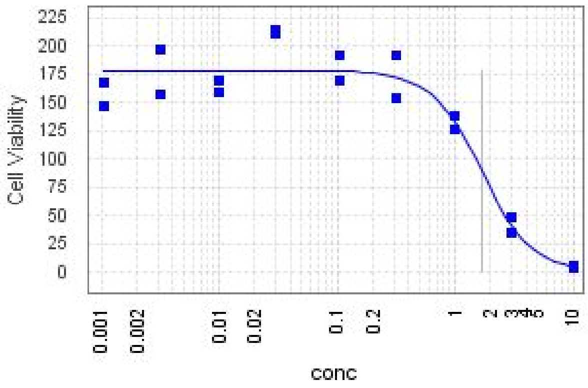

OCI-Ly1, GI50 = 1.68 µM

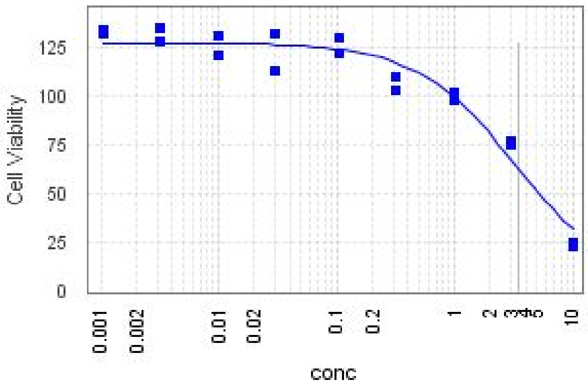

SU-DHL-4, GI50 = 3.45 µM

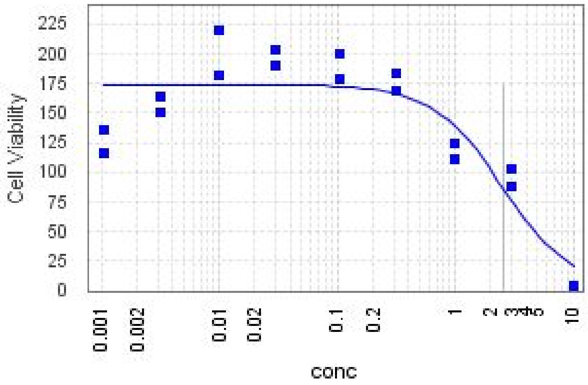

SU-DHL-6, GI50 = 2.55 µM

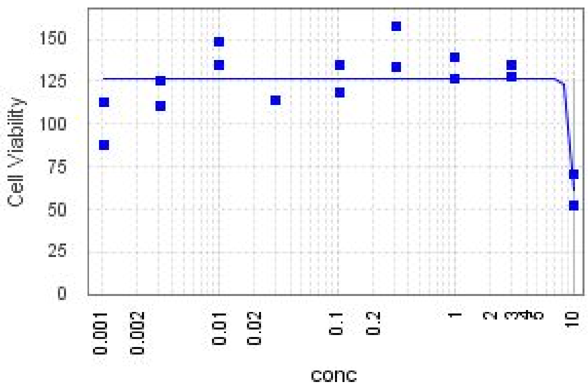

OCI-Ly3, GI50 > 10 µM

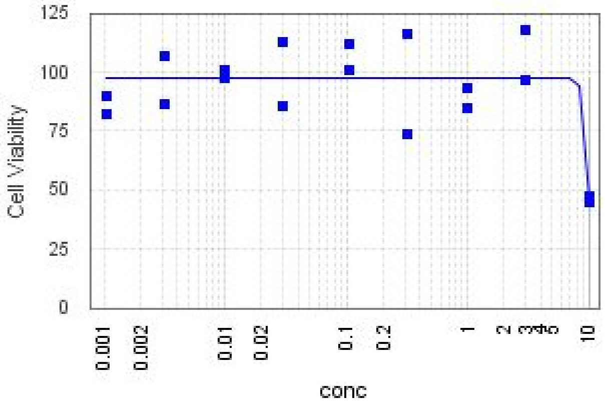

MM.1S, GI50 > 10 µM

8. Supplementary analytical data

8.1 LCMS traces for key compounds

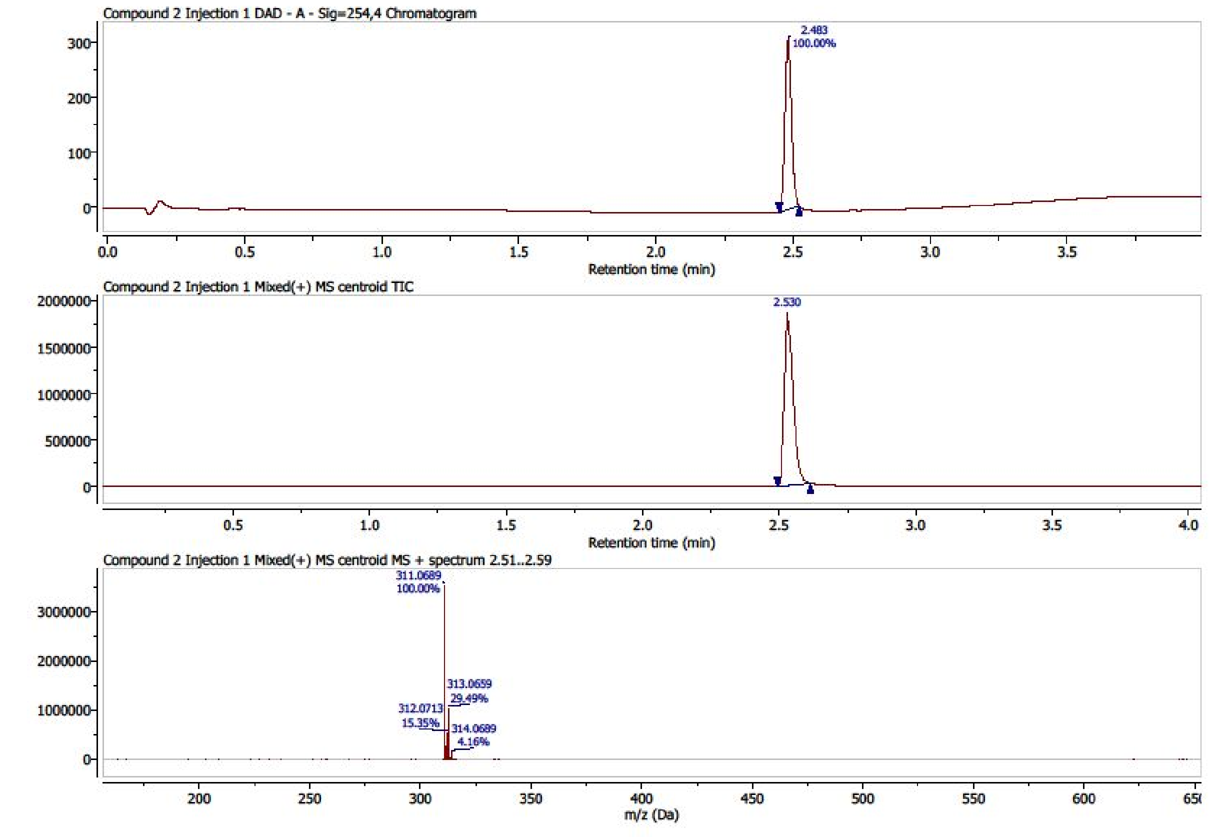

Compound 2

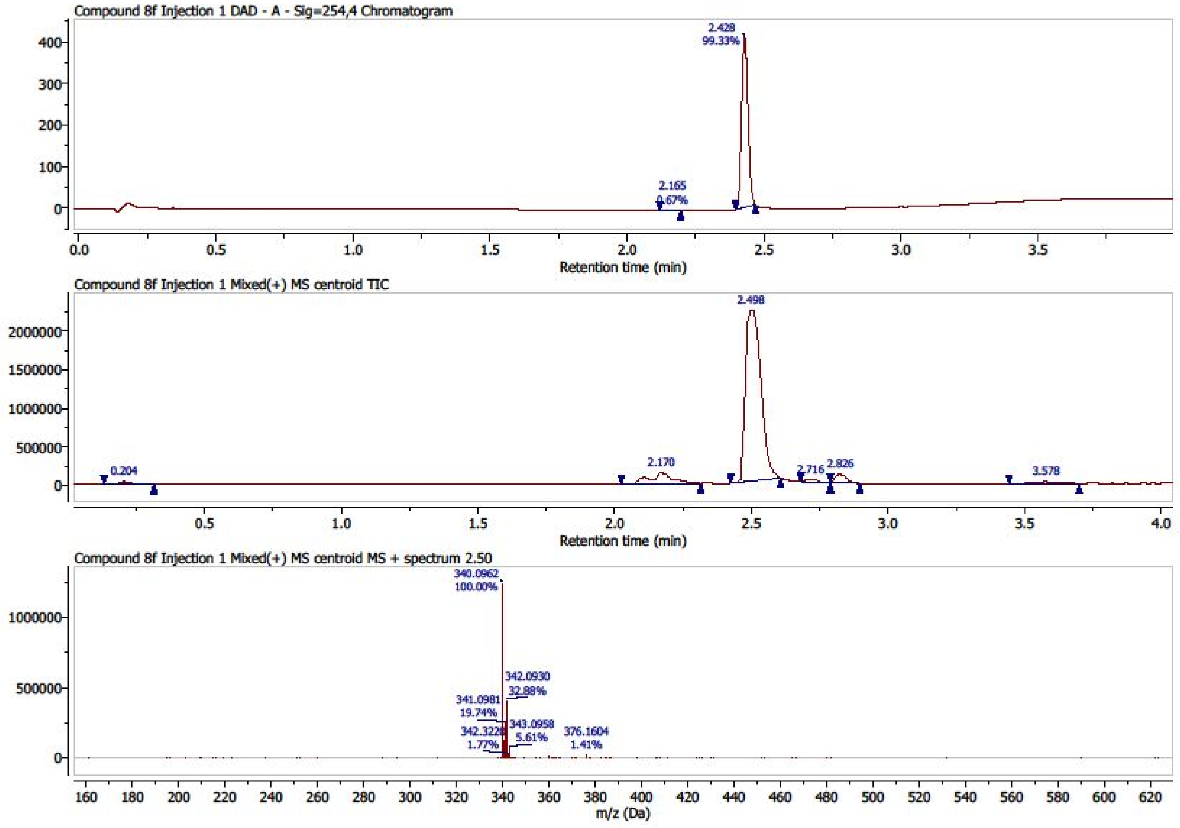

Compound 8f Injection 1 DAD - A - Sig=254,4 Chromatogram
2.428
99.33%
2.165
0.67%
Retention time (min)
Compound 8f Injection 1 Mixed(+) MS centroid TIC
2.498
0.204
2.170
2.716
2.826
3.578
Retention time (min)
Compound 8f Injection 1 Mixed(+) MS centroid MS + spectrum 2.50
340.0962
100.00%
342.0930
32.88%
341.0981
19.74%
342.3228
1.77%
343.0958
5.61%
376.1604
1.41%
m/z (Da)

Compound 8f

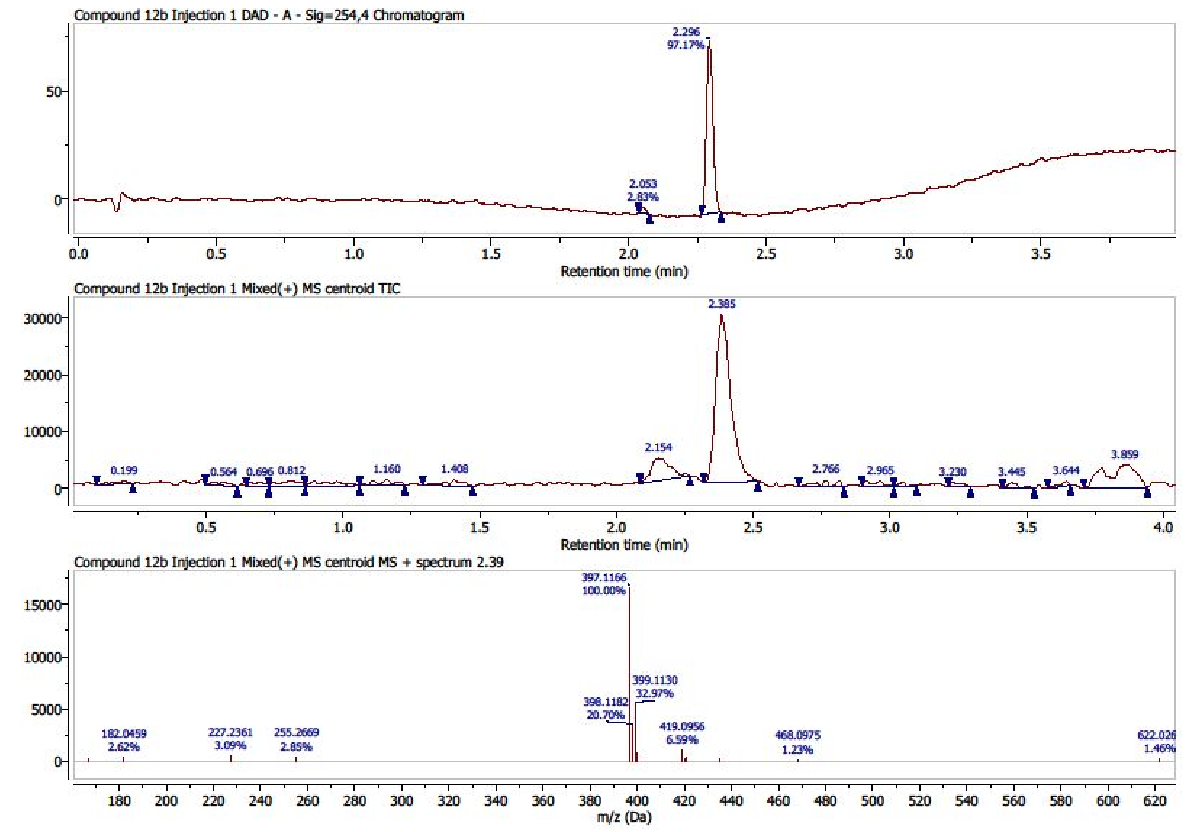

Compound 12b

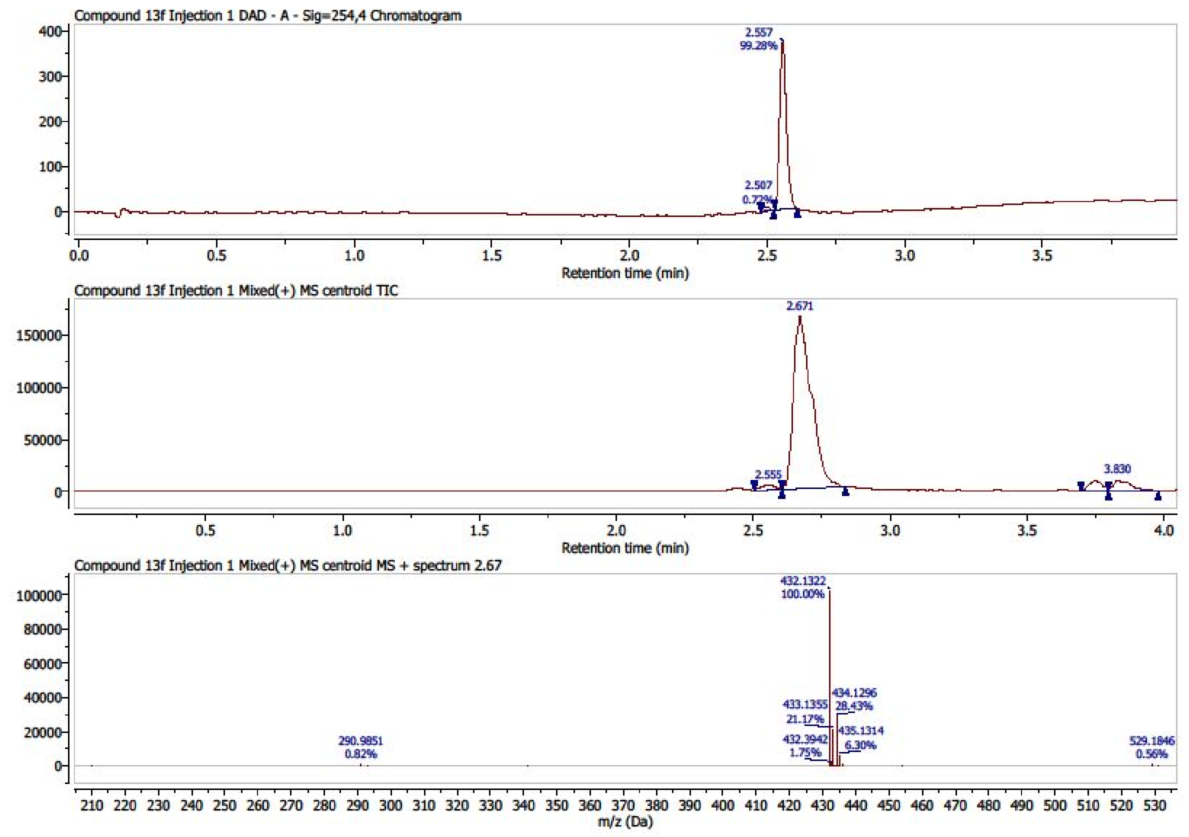

Compound 13f

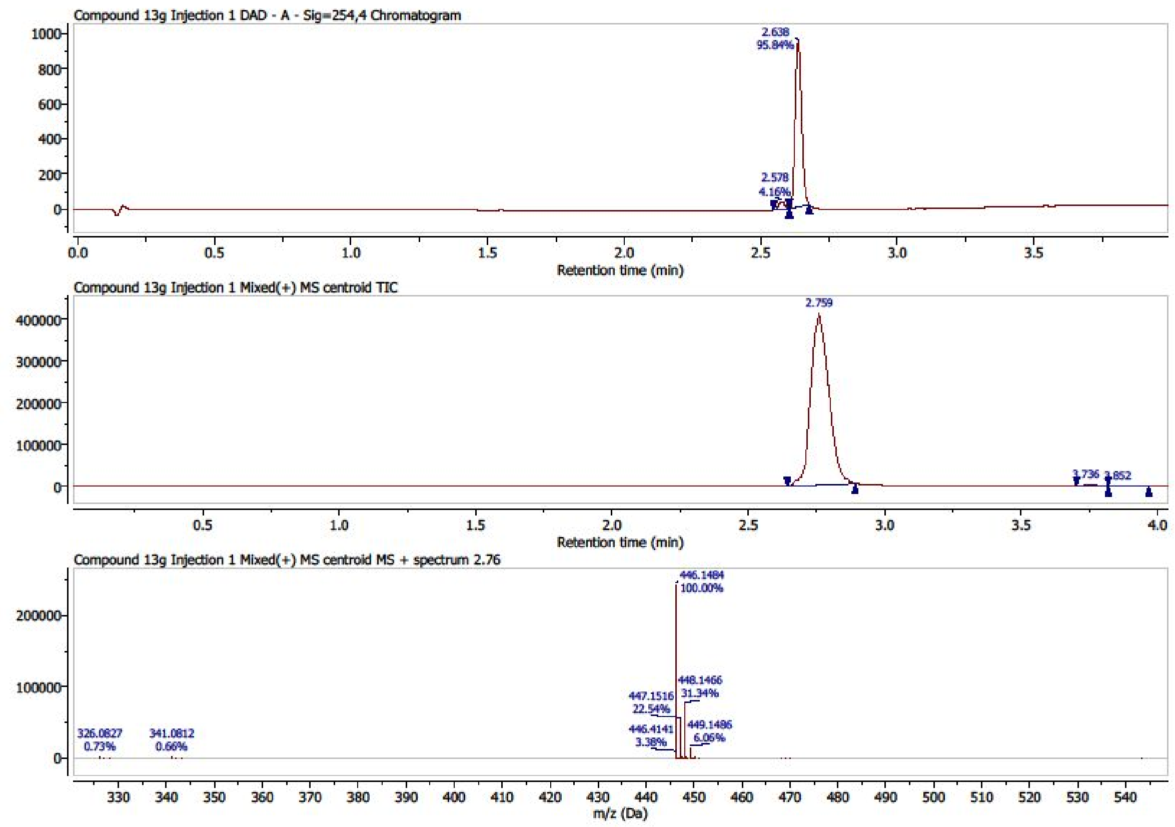

Compound 13g

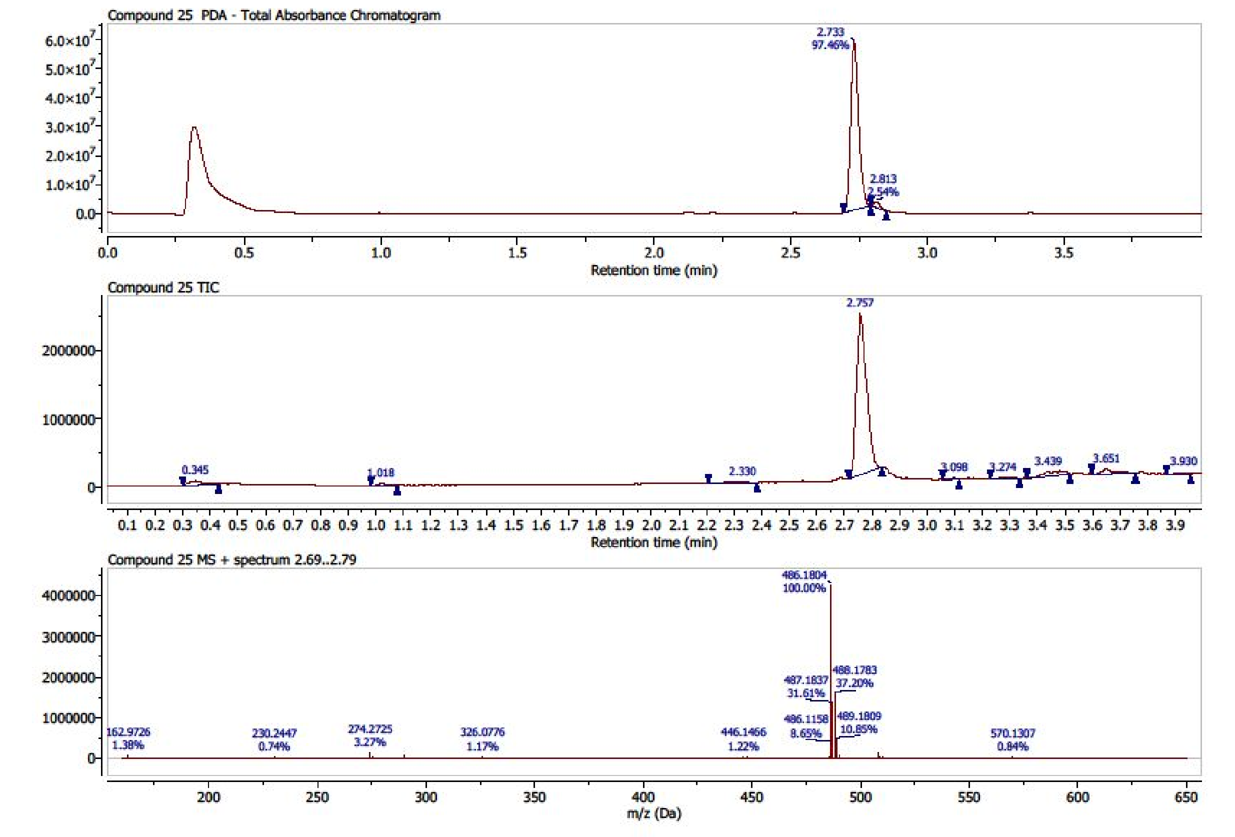
Compound 25 PDA - Total Absorbance Chromatogram
2.733
97.46%
2.813
2.54%
Retention time (min)
Compound 25 TIC
2.757
0.345
1.018
2.330
3.098
3.274
3.439
3.651
3.930
Retention time (min)
Compound 25 MS + spectrum 2.69..2.79
486.1804
100.00%
487.1837
31.61%
488.1783
37.20%
486.1158
8.65%
489.1809
10.85%
162.9726
1.38%
230.2447
0.74%
274.2725
3.27%
326.0776
1.17%
446.1466
1.22%
570.1307
0.84%
m/z (Da)

Compound 25

9. **References for supplementary information**

1. Kabsch, W., XDS. *Acta Crystallographica Section D* **2010,** *66* (2), 125-132.

2. Winter, G.; Waterman, D. G.; Parkhurst, J. M.; Brewster, A. S.; Gildea, R. J.; Gerstel, M.; Fuentes-Montero, L.; Vollmar, M.; Michels-Clark, T.; Young, I. D.; Sauter, N. K.; Evans, G., DIALS: implementation and evaluation of a new integration package. *Acta Crystallographica Section D* **2018,** *74* (2), 85-97.

3. Evans, P., Scaling and assessment of data quality. *Acta Crystallographica Section D* **2006,** *62* (1), 72-82.

4. Vonrhein, C.; Flensburg, C.; Keller, P.; Sharff, A.; Smart, O.; Paciorek, W.; Womack, T.; Bricogne, G., Data processing and analysis with the autoPROC toolbox. *Acta Crystallographica Section D* **2011,** *67* (4), 293-302.

5. McCoy, A. J.; Grosse-Kunstleve, R. W.; Adams, P. D.; Winn, M. D.; Storoni, L. C.; Read, R. J., Phaser crystallographic software. *Journal of Applied Crystallography* **2007,** *40* (4), 658-674.

6. Winn, M. D.; Ballard, C. C.; Cowtan, K. D.; Dodson, E. J.; Emsley, P.; Evans, P. R.; Keegan, R. M.; Krissinel, E. B.; Leslie, A. G. W.; McCoy, A.; McNicholas, S. J.; Murshudov, G. N.; Pannu, N. S.; Potterton, E. A.; Powell, H. R.; Read, R. J.; Vagin, A.; Wilson, K. S., Overview of the CCP4 suite and current developments. *Acta Crystallographica Section D* **2011,** *67* (4), 235-242.

7. Ghetu, A. F.; Corcoran, C. M.; Cerchietti, L.; Bardwell, V. J.; Melnick, A.; Privé, G. G., Structure of a BCOR Corepressor Peptide in Complex with the BCL6 BTB Domain Dimer. *Molecular Cell* **2008,** *29* (3), 384-391.

8. Emsley, P.; Cowtan, K., Coot: model-building tools for molecular graphics. *Acta Crystallographica Section D* **2004,** *60* (12 Part 1), 2126-2132.

9. G. Bricogne, E. B., M. Brandl, C. Flensburg, P. Keller, W. Paciorek, P. Roversi,; A. Sharff, O. S. S., C. Vonrhein, T.O. Womack, *BUSTER, Version 2.10.2, Global Phasing Ltd., Cambridge, United Kingdom* **2015**.

10. O.S. Smart, T. O. W., A. Sharff, C. Flensburg, P. Keller, W. Paciorek,; C. Vonrhein, G. B., *Grade, Version 1.2.9, Global Phasing Ltd., Cambridge, United Kingdom, 2014.*

11. Bruno, I. J.; Cole, J. C.; Lommerse, J. P. M.; Rowland, R. S.; Taylor, R.; Verdonk, M. L., IsoStar: A library of information about nonbonded interactions. *Journal of Computer-Aided Molecular Design* **1997,** *11* (6), 525-537.

12. Chen, V. B.; Arendall, W. B., III; Headd, J. J.; Keedy, D. A.; Immormino, R. M.; Kapral, G. J.; Murray, L. W.; Richardson, J. S.; Richardson, D. C., MolProbity: all-atom structure validation for macromolecular crystallography. *Acta Crystallographica Section D* **2010,** *66* (1), 12-21.
13. Davis, I. W.; Leaver-Fay, A.; Chen, V. B.; Block, J. N.; Kapral, G. J.; Wang, X.; Murray, L. W.; Arendall, W. B., III; Snoeyink, J.; Richardson, J. S.; Richardson, D. C., MolProbity: all-atom contacts and structure validation for proteins and nucleic acids. *Nucleic Acids Research* **2007,** *35* (suppl_2), W375-W383.
14. Wider, G.; Dreier, L., Measuring Protein Concentrations by NMR Spectroscopy. *Journal of the American Chemical Society* **2006,** *128* (8), 2571-2576.
15. Lin, M.; Tesconi, M.; Tischler, M., Use of 1H NMR to facilitate solubility measurement for drug discovery compounds. *International Journal of Pharmaceutics* **2009,** *369* (1), 47-52.
16. Saal, C.; Petereit, A. C., Optimizing solubility: Kinetic versus thermodynamic solubility temptations and risks. *European Journal of Pharmaceutical Sciences* **2012,** *47* (3), 589-595.
17. Workman, P.; Aboagye, E. O.; Balkwill, F.; Balmain, A.; Bruder, G.; Chaplin, D. J.; Double, J. A.; Everitt, J.; Farningham, D. A. H.; Glennie, M. J.; Kelland, L. R.; Robinson, V.; Stratford, I. J.; Tozer, G. M.; Watson, S.; Wedge, S. R.; Eccles, S. A.; An ad hoc committee of the National Cancer Research, I., Guidelines for the welfare and use of animals in cancer research. *British Journal of Cancer* **2010,** *102* (11), 1555-1577.
